# The Natural Product Domain Seeker version 2 (NaPDoS2) webtool relates ketosynthase phylogeny to biosynthetic function

**DOI:** 10.1016/j.jbc.2022.102480

**Published:** 2022-09-12

**Authors:** Leesa J. Klau, Sheila Podell, Kaitlin E. Creamer, Alyssa M. Demko, Hans W. Singh, Eric E. Allen, Bradley S. Moore, Nadine Ziemert, Anne Catrin Letzel, Paul R. Jensen

**Affiliations:** 1 Center for Marine Biotechnology and Biomedicine, Scripps Institution of Oceanography, University of California San Diego, La Jolla, California, USA; 2 Department of Biotechnology and Food Science, Norwegian University of Science and Technology (NTNU), Trondheim, Norway; 3 Molecular Biology Section, Division of Biological Sciences, University of California San Diego, La Jolla, California, USA; 4 Skaggs School of Pharmacy and Pharmaceutical Sciences, University of California San Diego, La Jolla, California, USA

**Keywords:** ACP, acyl carrier protein, BGC, biosynthetic gene cluster, C, condensation, ECH, enoyl-CoA hydratase, ER, enoyl reductase, FAS, fatty acid synthase, HR, highly reducing, KS, ketosynthase, NaPDoS, Natural Product Domain Seeker, NRPS, nonribosomal peptide synthetase, NR, nonreducing, PKS, polyketide synthase, PUFA, polyunsaturated fatty acid, PTM, polycyclic tetramate macrolactam, *trans*-AT, *trans*-acyl transferase

## Abstract

The Natural Product Domain Seeker (NaPDoS) webtool detects and classifies ketosynthase (KS) and condensation domains from genomic, metagenomic, and amplicon sequence data. Unlike other tools, a phylogeny-based classification scheme is used to make broader predictions about the polyketide synthase (PKS) and nonribosomal peptide synthetase (NRPS) genes in which these domains are found. NaPDoS is particularly useful for the analysis of incomplete biosynthetic genes or gene clusters, as are often observed in poorly assembled genomes and metagenomes, or when loci are not clustered, as in eukaryotic genomes. To help support the growing interest in sequence-based analyses of natural product biosynthetic diversity, here we introduce version 2 of the webtool, NaPDoS2, available at http://napdos.ucsd.edu/napdos2. This update includes the addition of 1417 KS sequences, representing a major expansion of the taxonomic and functional diversity represented in the webtool database. The phylogeny-based KS classification scheme now recognizes 41 class and subclass assignments, including new type II PKS subclasses. Workflow modifications accelerate run times, allowing larger datasets to be analyzed. In addition, default parameters were established using statistical validation tests to maximize KS detection and classification accuracy while minimizing false positives. We further demonstrate the applications of NaPDoS2 to assess PKS biosynthetic potential using genomic, metagenomic, and PCR amplicon datasets. These examples illustrate how NaPDoS2 can be used to predict biosynthetic potential and detect genes involved in the biosynthesis of specific structure classes or new biosynthetic mechanisms.

Increased access to DNA sequence data coupled with a better understanding of the molecular genetics of natural product biosynthesis are driving major advances in natural products research. These advances have been facilitated by webtools such as antiSMASH 6.0 (1) and PRISM 4 (2) that identify natural product biosynthetic gene clusters (BGCs) from assembled sequence data and provide insight into the types of small molecules produced (3). These tools have proven instrumental for genome mining research (4), while others have been developed to address more specific topics such as resistance-guided antibiotic discovery (5), biosynthetic gene biogeography (6), and *trans*-acyl transferase (*trans*-AT) substrate specificity (7). The Natural Product Domain Seeker (NaPDoS) is a specialized webtool used to assess biosynthetic diversity based on short sequence tags and thus does not require BGC assembly (8). It targets ketosynthase (KS) and condensation (C) domains to make broader predictions about the polyketide synthase (PKS) and non-ribosomal peptide synthetase (NRPS) genes, respectively, in which they reside. NaPDoS detects these domains and classifies them using a phylogeny-based scheme that reflects well-supported biosynthetic knowledge and established PKS and NRPS function.

Here, we introduce version 2 of the NaPDoS webtool (NaPDoS2), which includes an updated KS database and classification scheme that better reflects the expanded taxonomic distributions and functional diversity of PKSs. These enzymes produce structurally diverse polyketides ranging from lipids to macrolides, which represent an important source of compounds for pharmaceutical and other biotechnological applications (9). Polyketides also serve important ecological functions ranging from antioxidants (10) to allelochemicals (11) and thus can provide insight into how organisms interact with each other and the environment. PKSs share much in common with fatty acid synthases (FASs), generating compounds *via* the successive decarboxylative condensation and processing of acyl-CoA precursors (12). PKSs have been broadly divided into three types based on their organization and function (13) of which NaPDoS2 detects and classifies KSs associated with types I and II. While canonical type I PKSs have a modular organization and function in an assembly line fashion, some function iteratively while others (*e.g.*, *trans*-AT) lack a cognate acyl-transferase domain (9, 14). Similarly, canonical type II PKSs function iteratively and were originally best known to produce aromatic polyketides. Yet some type II PKSs function noniteratively (13), while others produce linear specialized metabolites (15). A central feature of PKSs is the KS domain, which in most cases catalyzes a Claisen condensation between the extender unit and the growing, thioester-linked polyketide chain. In recent years, our knowledge of KS functional diversity has expanded significantly to reveal new enzymology and diverse product outcomes across biology. The broad distributions and functional specificities among type I and II PKSs can, in many cases, be resolved phylogenetically (16, 17, 18), with these evolutionary relationships forming the basis of the NaPDoS2 classification scheme.

The NaPDoS2 website, available at http://napdos.ucsd.edu/napdos2/, includes many updated features that improve the usability of the tool for natural product discovery. Here, we report database and pipeline modifications that provide broader taxonomic coverage, better resolution among functionally characterized PKSs, a new subclassification scheme for type II PKSs, an increased capacity for processing large datasets, and the enhanced detection of eukaryotic KSs. Statistical validation tests have been used to select parameters for optimizing sensitivity and specificity, including both detection and classification accuracy based on query sequence length. The upgraded webtool was used to demonstrate the utility of NaPDoS2 for predicting biosynthetic potential in genomic, metagenomic, and amplicon datasets.

## Results and discussion

### Pipeline efficiency and interface upgrades

As in the original release (8), NaPDoS2 detects and classifies KS and C domains from nucleotide or amino acid sequence data. While both versions follow the same general workflow, substitution of the more computationally efficient program DIAMOND (19) for NCBI BLAST (20), eliminates the need for an extra Hidden Markov Model prefiltering step (Fig. S1). Speed improvements using DIAMOND are relatively modest for small jobs but can reach orders of magnitude for large datasets, especially those consisting of short query sequences (19). The NaPDoS2 pipeline executes most rapidly on amino acid sequences, which do not need to be translated. Although processing times increase with total nucleotide sequence length and the number of database matches, results for microbial genomes, assembled metagenomes, and PCR KS amplicons containing thousands of hits can typically be obtained within seconds to minutes (Table S1). These improvements enable users to perform large-scale analyses that were not feasible with the original NaPDoS release.

User interface upgrades include the addition of a “Domain Classification Summary” page that lists the total number of domains detected in the query data as grouped by their NaPDoS2 classification (Fig. S2*A*). This page provides the option to select classes of specific interest for more detailed investigation (Fig. S2*B*), which is particularly useful when large numbers of domains are detected. Each BGC represented in the database is linked to a summary page that includes a representative structure and the classification of each KS or C domain within that BGC (Fig. S2*C*). The independent classification of each domain is particularly useful given that a single gene or BGC can contain multiple domain types (21). New webtool features also include quick start instructions outlining the NaPDoS2 workflow (Fig. S3*A*) and a downloadable documentation and tutorial file.

### Database expansion

The primary goals for NaPDoS2 were to expand the KS database and classification scheme to include biosynthetic functions that were not represented in the original release, to supplement poorly populated classes, and to provide greater taxonomic coverage. One thousand four hundred seventeen new KS sequences were added, raising the database total to 1877 (average length 418 ± 49 amino acids), an increase of 308% (Fig. S4). Most of the additional sequences were derived from the MIBiG repository of experimentally verified BGCs (22), including new type II PKSs, type I fungal PKSs, and type I FASs from both bacterial and fungal sources. A few uncharacterized protist and metazoan sequences were included due to the scarcity of experimental verification among these groups. Although 84 C domain sequences were added, their classification scheme has not been updated and remains an important goal for future releases. The taxonomic breakdown of the current NaPDoS2 KS database sequences is 93.8% bacteria, 4.7% fungi, and 1.5% other eukaryotes (Table S2), reflecting the taxonomic skew of available experimental data. To improve result interpretation, database (match) IDs now display the BGC name, domain number, class, and subclass identifiers for each domain.

### Phylogeny-based KS classification

The ability to predict PKS and NRPS biosynthetic potential based on KS or C domains distinguishes NaPDoS2 from other bioinformatic tools. The domain classification scheme is derived from KS sequence phylogenies and their relationships to established biochemical functions, gene architectures, and structural features of the compounds produced. While not all classes and subclasses are monophyletic, functionally coherent clades form the basis of the classification scheme. Phylogenies generated from the updated KS database allowed us to establish 41 class and subclass assignments associated with type I and II PKSs and FASs (Table S2 and Fig. S5). This represents an increase of 410% over the original release. To verify the sufficiency of using KS domains to indicate broader PKS context, we assessed the genomic neighborhoods of KSs detected in a variety of genomic and metagenomic datasets. In all cases, the NaPDoS2 KS classification agreed with the antiSMASH 6.0 (1) classification (Fig. S6). In some cases, NaPDoS2 provided more detailed information including the identification of type II subclasses, omega-3 polyunsaturated fatty acid (PUFA) KSs, and highly, partially, and nonreducing fungal KSs. NaPDoS2 can also distinguish among different KS classes within a single gene or BGC (Fig. S6).

**Figure 1:**
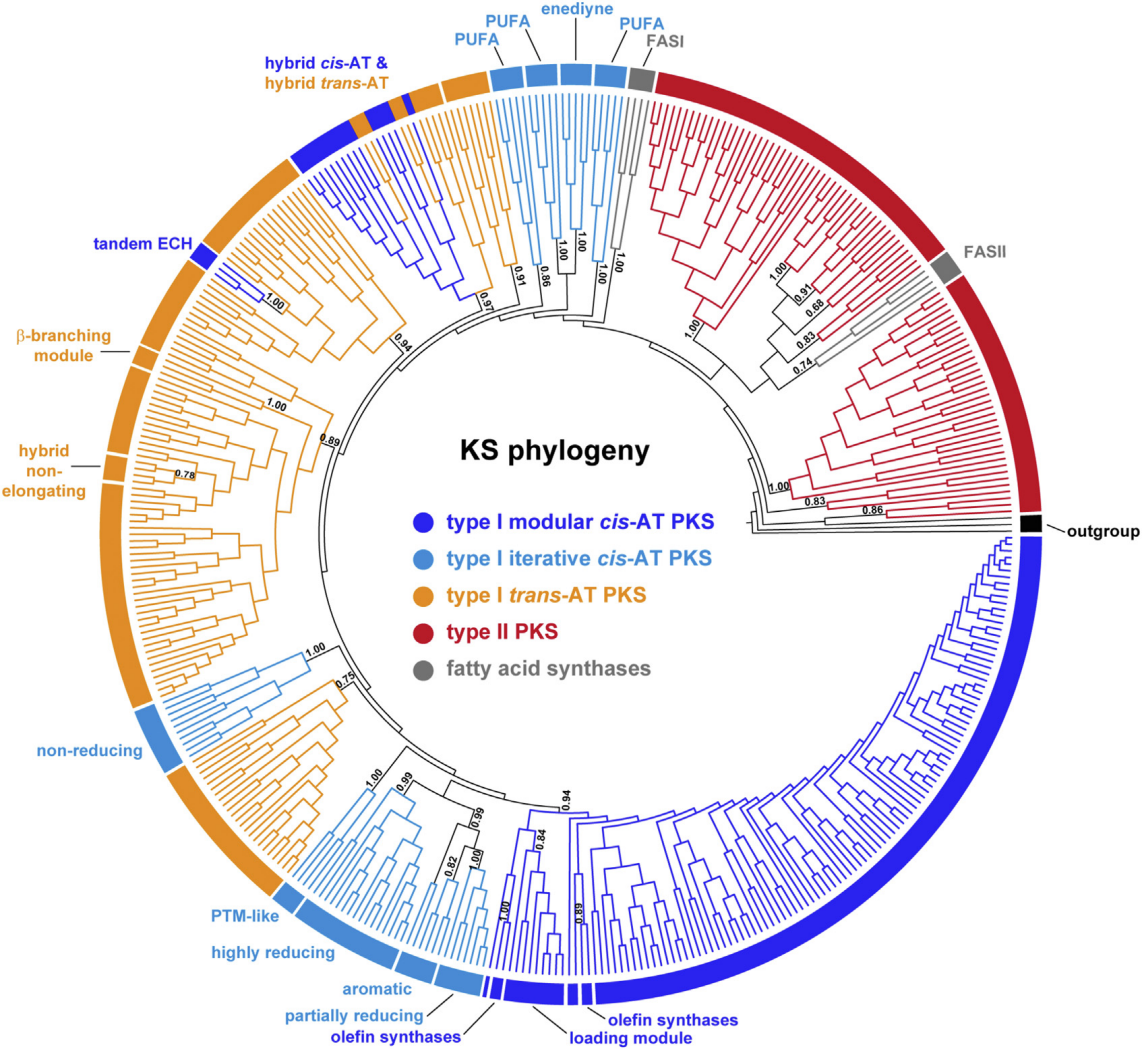
**KS phylogeny-based classification**. Maximum likelihood phylogeny generated from 414 KS sequences. Clades are color-coded and labeled according to their NaPDoS2 classification. Transfer bootstrap expectation (TBE) support was estimated using Booster (72). The full name of each sequence can be viewed in Fig. S7, which can be used to link a query match to a specific location in the tree. Thiolases from *Escherichia coli*, *Zoogloea ramigera*, and *Streptomyces avermitilis* were used as outgroups. An expanded phylogeny of the type II KSs is presented in Figure 3. FAS, fatty acid synthase; KS, ketosynthase; NaPDoS2, Natural Product Domain Seeker version 2; PKS, polyketide synthase; PUFA, polyunsaturated fatty acid.

**Figure 2:**
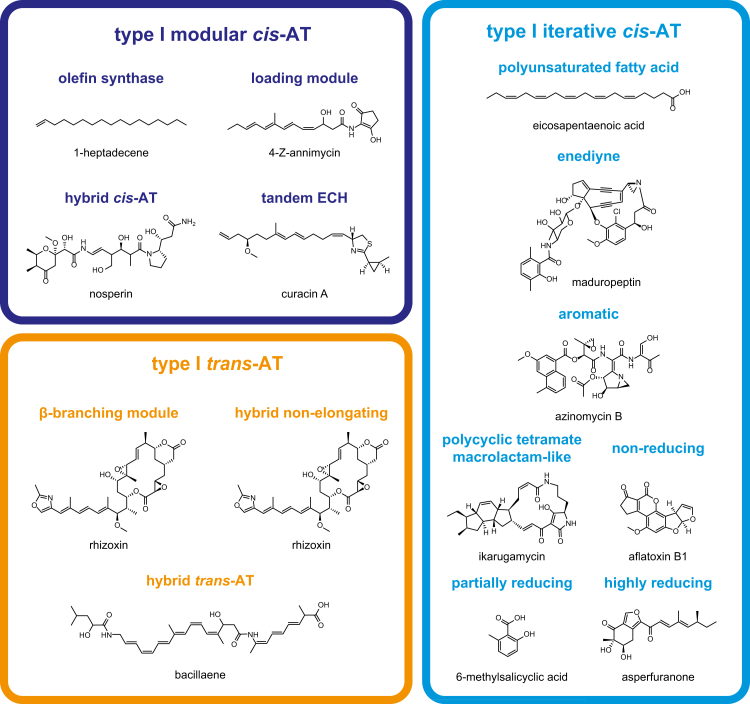
**Example structures for the type I KS classes and subclasses recognized by NaPDoS2**. Descriptions of each KS type, associated metadata, and references can be found in Fig. S5 and Table S3. Information on each structure can also be accessed from the BGC tab on the website (note: 1-heptadecene = *Moorea producens* olefin synthase, 4-Z-annimycin = annimycin, and eicosapentaenoic acid = *Schizochytrium* polyunsaturated fatty acid on the website). Colors correspond to Figure 1. KS, ketosynthase; NaPDoS2, Natural Product Domain Seeker version 2.

With the classification scheme established, a simplified reference tree was generated using 414 sequences representing all class and subclass assignments (Fig. 1). Broadly, this reference phylogeny delineates the well-established relationships between FASs and type I and II PKSs (17). The enhanced classification of type I FAS KSs from bacteria/fungi, protists, and metazoa allows users to better distinguish between KSs associated with specialized metabolism and fatty acid biosynthesis across diverse taxa. The expanded eukaryotic type I coverage includes KSs from fungal iterative *cis*-AT PKSs and several protist (Phyla Amoebozoa and Apicomplexa) and metazoan (Phyla Chordata, Echinodermata, and Nematoda) PKSs, including those linked to characterized natural products from *Caenorhabditis elegans* (23) and *Dictyostelium discoideum* (24). The seven type I PKS classes described in the original NaPDoS release have been reorganized into three classes (modular *cis*-AT, iterative *cis*-AT, and *trans*-AT) and 14 subclasses in the NaPDoS2 release. Example structures associated with each KS type are shown in Figure 2 with associated metadata (Table S3) and descriptions of each KS type (Fig. S5 and website documentation) provided to help connect KSs with structures and relevant references.

### Modular cis-AT KSs

The modular *cis*-AT class comprises KSs associated with the canonical assembly line PKSs (*e.g.*, erythromycin biosynthesis) and now includes four subclasses of which two [olefin synthase and tandem enoyl-CoA hydratase (ECH)] are new to NaPDoS2 (Fig. 1). The olefin synthase subclass is best known from cyanobacteria and is associated with the formation of a terminal olefin on a fatty acyl precursor (25). These KS sequences form two clades in the reference tree, which reflects the sporadic distribution of the OLS pathway among cyanobacteria (25). The tandem ECH subclass is associated with gene cassettes that introduce a branch at the β-keto position. In these PKSs, the KS domain is located immediately downstream of tandem ECH and enoyl reductase (ER) domains, which catalyze decarboxylation and reduction reactions, respectively, as seen in cylindrocyclophane biosynthesis (26) and reviewed elsewhere in detail (27). Most of the modular *cis*-AT KSs in the database are not associated with a specialized function and thus are not assigned to a subclass.

### Iterative *cis*-AT KSs (bacteria)

The iterative *cis*-AT class (iPKSs) maintains a modular organization, with multiple enzymatic domains on a single protein, yet instead of functioning as an assembly line these enzymes catalyze an iterative series of elongation steps (28). The expanded NaPDoS2 classification scheme now includes seven iPKS subclasses. The four observed in bacteria include the aromatic and polycyclic tetramate macrolactam (PTM) subclasses (29), both of which are new to NaPDoS2, and the enediyne and PUFA subclasses, which were identified in the original release. PUFA KSs now form three clades in the reference tree, which correlate with the three KS domains typically present in PUFA PKSs (30). Aromatic iPKSs generally produce simple monocyclic or bicyclic aromatic compounds that are distinct from enediynes and PUFAs (29). The PTM-like iPKSs produce complex compounds containing a macrocyclic lactam with an embedded tetramic acid moiety fused with a polycyclic system (*i.e.*, 2–3 rings) derived from polyene chains (29). The ability to quickly recognize PTM biosynthetic potential provides opportunities to expand on the number of compounds discovered in this unusual and often biologically active class (31). New iterative type I PKSs continue to be discovered, including some that are unexpectedly abundant and widespread in the genus *Streptomyces* (31), providing opportunities to expand this class in future updates beyond the seven subclasses currently recognized by NaPDoS2.

### Iterative *cis*-AT KSs (fungi)

Iterative *cis*-AT KSs are also observed in fungi and can be delineated into highly reducing (HR), partially reducing, and NR iPKS subclasses (32). These subclasses differ in the number and type of β-keto processing ketoreductase, dehydratase, and ER domains present. HR iPKS BGCs, which contain all three β-keto processing domains, often contain a C-methylation domain responsible for ɑ-carbon methylation and some are fused with NRPS modules (33). Their products are usually linear or cyclic, nonaromatic compounds. Partially reducing iPKS BGCs lack ER and sometimes also dehydratase domains. Due to their similarity to bacterial aromatic iPKSs, they have been hypothesized to originate from a horizontal gene transfer event between bacteria and fungi (34). NR iPKS BGCs lack all three β-keto processing domains and have specialized domains that facilitate starter unit loading and product folding (33).

### Trans-AT KSs

KS domains from *trans*-AT PKSs form multiple clades in the reference tree (Fig. 1), which may reflect KS substrate-specificity (35). Nonetheless, three functionally distinct subclasses can be recognized by NaPDoS2 of which those associated with β-branching modules and hybrid, nonelongating KSs are new to NaPDoS2. The β-branching module, comprising a KS domain, a cryptic “B” domain, and an acyl carrier protein (ACP) domain, represents one of the mechanisms for the formation of β-branching in *trans*-AT PKSs (36, 37). Hybrid, nonelongating KSs (KS^0^) typically follow an NRPS module, lack the catalytic histidine required for elongation, and are proposed to facilitate the transfer of the growing polyketide to the next module (7, 38). The hybrid *trans*-AT KS subclass clades with the hybrid *cis*-AT subclass and functions similarly in modules that lack AT domains.

### Type II KSs

**Figure 3:**
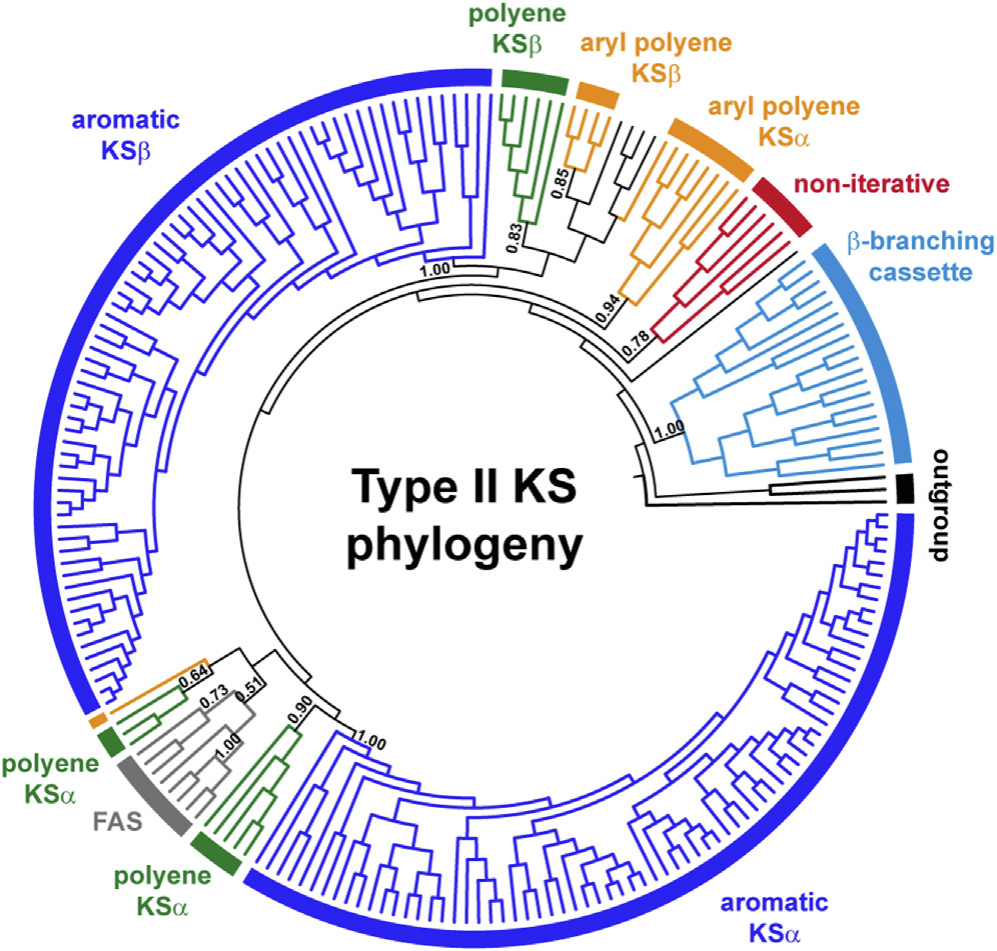
**Type II KS phylogeny-based classification**. Maximum likelihood phylogeny of 212 KS sequences. Clades are color-coded and labeled according to their NaPDoS2 classification. TBE bootstrap support was estimated using Booster (72). See Fig. S8 for sequence annotations. Thiolases from *E. coli*, *Z. ramigera*, and *S. avermitilis* were used as outgroups. Type II KS sequences that lack further annotation have yet to be assigned a functional class. KS, ketosynthase; NaPDoS2, Natural Product Domain Seeker version 2; TBE, transfer bootstrap expectation.

**Figure 4:**
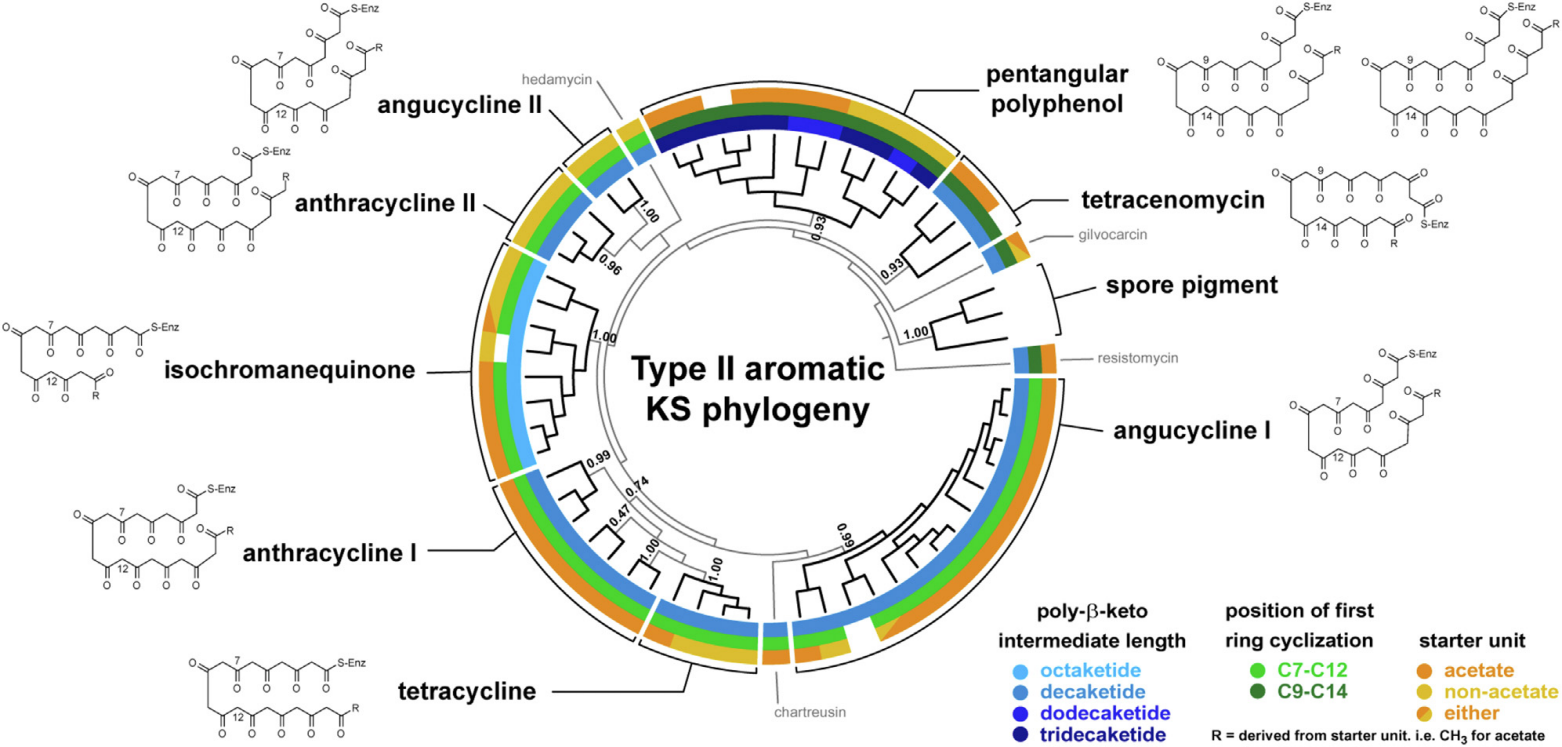
**Type II aromatic KS phylogeny-based classification**. Maximum likelihood phylogeny of concatenated KSα and β subunits from 59 type II BGCs. Clades are annotated based on NaPDoS2 classification. Structural motifs associated with subclasses shown in colored rings (white, not determined). See Fig. S9 for sequence annotations and Table S3 for biosynthetic references. TBE bootstrap support was estimated using Booster (72). BGCs, biosynthetic gene clusters; KS, ketosynthase; NaPDoS2, Natural Product Domain Seeker version 2; TBE, transfer bootstrap expectation.

**Figure 5:**
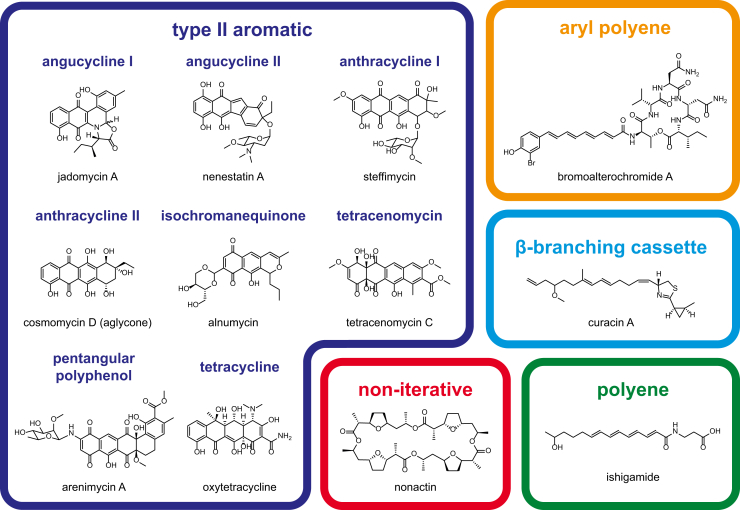
**Example structures for the type II KS classes and subclasses recognized by NaPDoS2**. Descriptions of each KS type, associated metadata, and references can be found in Fig. S5 and Table S3. Information on each structure can also be accessed from the BGC tab on the website (note: bromoalterochromide A = alterochromide on the website). Colors correspond to Figure 3. BGC, biosynthetic gene cluster; KS, ketosynthase; NaPDoS2, Natural Product Domain Seeker version 2.

A major aim for the NaPDoS2 upgrade was to expand the classification of type II KS domains based on established functions and evolutionary relationships (39). A phylogeny of the type II KS domains in the updated database (Fig. 3) provided sufficient resolution to delineate five functionally defined classes and four sequences that have yet to be assigned a functional class (annotated as type II unclassified). This represents a major improvement over the original NaPDoS release, which simply identified a sequence as being associated with a type II PKS. The most highly populated of the five type II KS classes recognized by NaPDoS2 are associated with PKSs that function iteratively to produce reactive poly-β-ketoacyl intermediates that cyclize to polycyclic aromatic compounds. The type II aromatic class could be further delineated into nine functionally defined subclasses based on a concatenated alignment of the two KS subunits, which generated a better resolved phylogeny (Fig. 4). Example structures (Fig. 5) along with the associated metadata (Table S3) help connect each KS type with structures and relevant references.

Type II KSs function as a heterodimer, with the KSα subunit responsible for the iterative elongation of a nascent poly-β-keto chain, the KSβ subunit determining the chain length (also referred to as the chain-length factor), and both subunits mediating the first ring cyclization reaction (38, 40). The KSα and KSβ subunits are implemented as individual sequences in the NaPDoS2 workflow thus allowing them to be distinguished. To define these subclasses, the tree topology was compared with poly-β-keto chain length, carbon-carbon position of the first ring cyclization, type of starter unit, and the core and intermediate structures of the products (Fig. 4). These subclasses are consistent with previous phylogenies (16, 41) and chemotypes identified in PKMiner (42) and antiSMASH version 5.0 (43) based on full-length BGC analyses. KSs from several unusual type II aromatic PKSs that are poorly represented in the database (*e.g.*, resistomycin biosynthesis) were not assigned subclasses and are instead annotated as “type II aromatic unclassified” in NaPDoS2.

The four remaining type II KS classes are more unusual and include one that is responsible for introducing β-branching in *trans*-AT PKSs. These KSs are associated with HMGS or HCS (3-hydroxy-3-methylglutaryl coenzyme A synthase) gene cassettes that encode several monodomain proteins including a decarboxylating KS that can be resolved in the type II KS phylogeny (Fig. 3) (27). Another unusual type II KS class recognized in the phylogeny functions noniteratively. The KSs associated with these enzymes facilitate the incorporation of acetate, propionate, and succinate building blocks leading to the production of macrocyclic compounds such as the nonactin antibiotics, in which case they catalyze C-O bond formation (44). Noniterative type II PKSs contain multiple type II and III KSs in which each domain is responsible for a single condensation step (45, 46). The remaining type II classes represent KSs associated with the production of highly reduced polyenes or aryl polyenes (Fig. 3), with the latter representing the most prominent biosynthetic family observed across a wide taxonomic distribution of bacterial genomes (47). Similar to type II aromatic PKSs, polyene and aryl polyene PKS also contain KSα and KSβ subunits with biosynthesis proceeding through a series of alternating elongation and reduction steps (15, 48, 49). Aryl polyene PKSs also possess homodimeric KSs that function to complement the heterodimer (15, 49). These are most similar to KSs observed in type II FASs (not shown in the tree) and are classified as such in the NaPDoS2 database.

### Performance evaluation

Recognizing that computational prediction algorithms are seldom perfect, leave-one-out cross validation (50) and receiver operating characteristic (ROC) curves (51) are powerful statistical tools for quantifying sensitivity and specificity but require calibration using known positive and negative control datasets that maximize discriminatory power. A set of positive controls (213 sequences) for evaluating NaPDoS2 performance was obtained by clustering all full-length, experimentally verified KS domains in the NaPDoS2 database at 50% amino acid identity. These sequences belong to five conserved domain families within the condensing enzyme superfamily of the NCBI Conserved Domain Database (Fig. S10, *A* and *B*), which encompasses enzymes that catalyze decarboxylating or nondecarboxylating Claisen-like condensation reactions for the synthesis and degradation of fatty acids and polyketides from all kingdoms of life (23% eukaryota, 70% bacteria, and 7% archaea). Negative controls (308 sequences) were selected from condensing enzyme families falling immediately outside the NaPDoS2 clades and similarly clustered at 50% amino acid identity (Fig. S10*A*). These negative controls, which include beta-ketoacyl-ACP synthases, ketoacyl-ACP synthases III, type III chalcone and stilbene synthases, thiolases, and sterol carrier protein-associated thiolases, were augmented with additional sequences (52) (Fig. S10*C*) and KSs from type III PKSs retrieved from MIBiG 2.0 (Fig. S10*D*).

**Figure 6:**
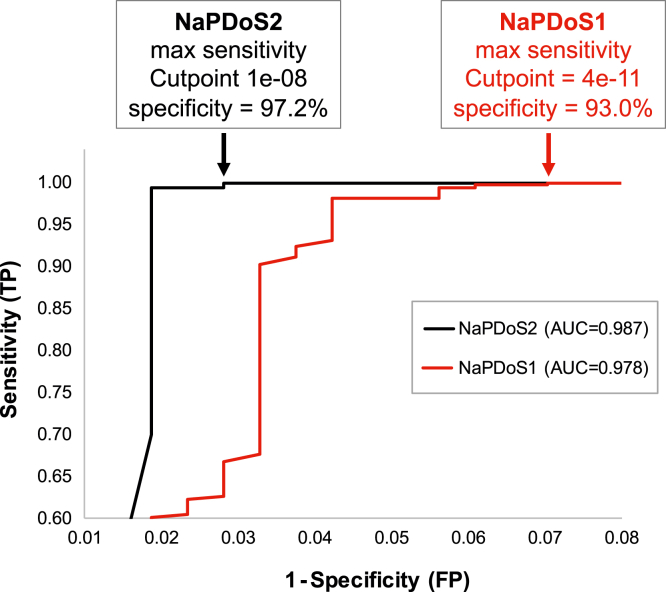
**Receiver operating characteristic curves for NaPDoS and NaPDoS2**. Nonredundant sets of 213 positive and 308 negative control KS domains were compared based on the BLASTP e-values of their closest non-self-match in each NaPDoS database. Optimal cutoff values were selected to maximize sensitivity. FP, false positives; KS, ketosynthase; NaPDoS2, Natural Product Domain Seeker version 2; TP, true positives.

Leave-one-out cross-validation scores were generated for positive and negative control sequences based on the e-values of their closest nonself match using both the original NaPDoS and NaPDoS2 KS reference databases. ROC curves were calculated from these scores to generate area under the curve (AUC) values (Fig. 6*A*), demonstrating the superior performance of NaPDoS2 (AUC = 0.987) *versus* the original release (AUC = 0.978). ROC curves were further used to establish e-value cutoff points that maximize sensitivity and minimize false positives, identifying optimum values as 1e-8 for NaPDoS2 and 4e-11 for the original NaPDoS release. Although these cutoff values provided equivalent sensitivity (detection of true positives) for their respective algorithms, the highest achievable specificity obtainable for NaPDoS was 93.0% (7% false positive rate) *versus* 97.2% for NaPDoS2 (2.8% false positive rate). This improvement is most likely explained by the expanded database underlying the NaPDoS2 pipeline. Consequences of these statistically identified differences in real-world use cases are presented in the “NaPDoS2 applications” section below.

**Figure 7:**
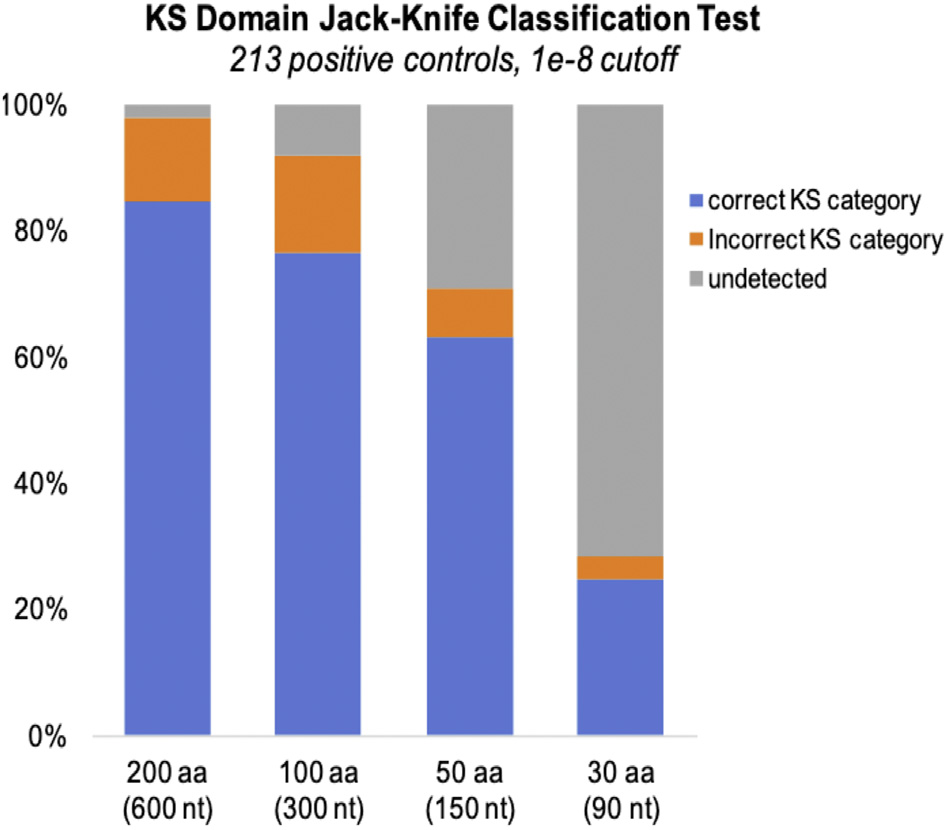
**Effect of query size on KS detection and classification accuracy**. Classifications were based on a 1e-8 BLASTP e-value cutoff score for the closest non-self-database match. Test sequences of varying lengths were obtained as overlapping sliding window subsequences covering the full length of 213 nonredundant, positive control KS domains. KS, ketosynthase.

The effects of partial KS sequences on detection and classification accuracy were evaluated using both full-length database sequences (typically 425 amino acids) and shorter overlapping subsets of 30, 50, 100, and 200 amino acids (aa) covering the entire length of these sequences. These subsets were designed to mimic domain fragments encountered in draft genomes, metagenomic assemblies, or KS amplicon sequences. Using the previously established 1e-8 cutoff for maximum sensitivity, NaPDoS2 detected 99% of the 200aa length subsequences as KS domains, of which 85% were correctly classified (Fig. 7). Detection and classification accuracy declined with shorter sequences, falling dramatically for sequences <50 amino acids. Length-dependent performance degradation was also observed using 1e-5 and 1e-10 e-values as cutoff scores (Fig. S11). These results illustrate the difficulty of analyzing unassembled, next-generation sequencing reads and short contigs covering partial domain fragments.

Based on performance evaluation results, default NaPDoS2 parameters were set at an e-value of 1e-8 and a minimum alignment length of 200 amino acids; however, users may choose to adjust these settings for their individual datasets. Sensitivity declines for partial KS domains can be partially offset by decreasing BLAST stringency, at the cost of increasing false positives and misclassifications. PCR-generated amplicons can provide better sensitivity than similarly sized random sequence fragments but may still be too short to obtain accurate classifications. Confident assignments to poorly populated classes or subclasses are particularly challenging given the sequence diversity observed within highly populated classes and subclasses (Fig. S12). Some ambiguities may be resolved by using NaPDoS2 sequence alignments to generate detailed phylogenetic trees, but others will remain until additional functional studies are reported.

### NaPDoS2 applications

Large-scale performance evaluations targeting genomic, metagenomic, and amplicon sequence data from a variety of bacterial, fungal, metazoa, and environmental sources were conducted to demonstrate the utility of NaPDoS2 in identifying polyketide biosynthetic potential (Table 1; accession numbers in Table S3). These examples include the integration of NaPDoS2 output with other webtools (Fig. S3*B*).

**Table 1:** NaPDoS2 applications

Table #	Application	Data type	Biological source	Dataset	Ref.
S4, S5	Bacterial type II KS	Genome	Bacteria	118 *Salinispora* strains	(53)
S6	Fungal KS & FAS	Genome	Fungi	27 Fungal spp.	(55)
S7	Fungal KS & FAS	Genome	Fungi	159 Fungal MIBiG 2.0 PKS BGCs	(22)
S7	Environmental type II KS	Amplicon	Environmental DNA	147 KS clones from soil	(61)
S8	Eukaryotic KS & FAS	Genome	Metazoa	*Elysia chlorotica*	(56, 57)
S9	Environmental type I & II KS, FAS	Metagenome	Environmental DNA	20 Marine sediment samples	(60)
S10	Environmental type I KS	Amplicon	Environmental DNA	eSNaPD v2.0 KS sequences from soil	(62)
S11	Environmental type II KS	Amplicon	Environmental DNA	Type II KS sequences from 12 soil samples	(63)
S12	Environmental and cultured type I & type II KS	Amplicon	Bacteria, environmental DNA	Type I and II KS sequences from lake sediment and enrichment cultures	(64)

The utility of NaPDoS2 was demonstrated across a variety of data types and biological sources. Details for each analysis can be found in Tables S4–S12 as summarized below. Table S3 lists accession numbers for all analyses.

#### Genomes

While the original NaPDoS release simply identified KSs as type II, the new release provides more sensitive detection and more detailed classifications. We analyzed 118 *Salinispora* genomes (53) with NaPDoS2 and detected a total of 662 type II KS domains in contrast to 363 using the original release (Table S4). The type II KSs detected by NaPDoS2 were delineated into seven functionally defined classes and subclasses; those that were unassigned may represent new functional diversity. A broader summary of all KSs detected in these genomes provided the first evidence that *S. arenicola* has the potential to produce PTMs (Table S5), a class of structurally complex natural products that exhibits diverse biological activities (54). These results provide new insight into the biosynthetic potential of this marine actinomycete genus.

Another important NaPDoS2 improvement is the ability to detect and classify eukaryotic KS sequences. This is illustrated by the analysis of 27 taxonomically diverse fungal genomes (55), where KSs ranged from one in *Malassezia globosa* to 50 in *Aspergillus niger* (Table S6) and the majority could be assigned to the HR subclass. We next analyzed all 159 fungal PKS BGCs in the MIBiG 2.0 repository (22) and identified 182 KS domains, all of which matched MIBiG 2.0 descriptions and literature reports (Table S7). In contrast, the original NaPDoS release only identified 14 KS domains from these same BGCs. Although relatively few metazoan PKSs have been experimentally characterized, NaPDoS2 recovered the recently described FAS and FAS-like KSs from the *Elysia chlorotica* sacoglossan genome (Table S8) (56, 57) and correctly classified them as metazoan type I FASs. A phylogenetic tree generated using NaPDoS2 confirmed their divergence from previously characterized animal FASs (data not shown). In an exploratory search for KSs in other eukaryotic genomes, NaPDoS2 detected 37 modular *cis*-AT domains, 17 type I FAS domains, 2 type II FAS domains, and 1 protist-type KS domain from the dinoflagellate *Symbodium minimus* (58), and six type II FAS domains and one type II KSα aromatic anthracycline domain from the diatom *Nitzschia inconpicua* (59), thus further validating its utility for analyzing eukaryotic sequences. While these data show that NaPDoS2 can detect and classify KS domains from complex metazoan datasets, it is best suited for predicted proteins, translated coding sequences, or transcriptomes, since it cannot excise introns associated with eukaryotic sequence data.

#### Metagenomes

A single NaPDoS2 analysis can provide a simultaneous overview of both bacterial and eukaryotic polyketide and fatty acid biosynthetic potential in large metagenomic datasets. While it is not reliant on fully assembled BGCs, assembled contigs are recommended to avoid the reduced classification accuracies associated with short sequence reads (Figs. 7 and S11). To illustrate this application, we assessed 20 assembled marine sediment metagenomes deposited in the Paired Omics Data Platform (60). We observed a wide range in the numbers and types of KSs detected, which can provide important insight when selecting samples for further study (Table S9). These results show how NaPDoS2 can be used to identify samples with the potential to produce compounds in rare but biologically active classes, such as enediynes and PTMs, to expand on poorly understood metazoan PKS diversity and, in cases with low sequence similarity to database or BLAST matches, detect new functional diversity. Trimmed domains can be used as search queries to assess broader genomic context (Fig. S6), compare with previously reported BGCs (22), and potentially identify the host organism when phylogenetic markers are encountered in the KS-containing contig.

#### Amplicons

NaPDoS2 is particularly useful for the analysis of KS/C domain PCR amplicon datasets, where it can be used not only to classify sequences into specific functional categories and assign a top BGC product match but also to assess primer specificity and remove nontarget sequences prior to downstream analysis. We illustrate the applications of NaPDoS2 using four KS amplicon datasets, starting with 147 KS sequences cloned from soil eDNA using type II–specific KS primers (61). Both versions of NaPDoS identified all 147 KSs as type II, while NaPDoS2 further delineated them into three type II aromatic subclasses, which agrees with the original report (Table S7). We next compared the NaPDoS2 output with that from eSNaPD v 2.0, which relates KS amplicon sequences to a database of characterized BGCs (6, 62). NaPDoS2 detected all 381 KS sequences in the eSNaPD v 2.0 New Mexico desert soil library “NM_KS_ARRY_LIB01” dataset (62) and classified the vast majority as *cis*-AT modular (Table S10). Additionally, NaPDoS2 classified virtually all KS sequences within what eSNaPD2 v 2.0 listed as novel clusters, providing new information about the biosynthetic diversity within this library (6, 62) (Table S10).

Larger KS amplicon datasets generated from soil eDNA and lake sediment enrichment cultures using type I and II specific primers were also evaluated (63, 64). While the original analyses estimated biosynthetic potential based solely on the closest MIBiG repository match, NaPDoS2 further delineated the sequences into specific type I and type II classes and subclasses. Analysis of the type II soil amplicons (63) revealed that many were not identified as KSs and appear to represent off-target sequences. Of those identified as KSs, a number were classified by NaPDos2 as type II FASs and a few as type I PKSs (Table S11). This illustrates the application of NaPDoS2 to assess primer specificity and identify potential nontarget KS sequences prior to downstream analyses.

Finally, we addressed the question of whether lowering the minimum alignment length for amplicons might increase the number of false positives, as previously observed for random domain fragments (Fig. 7). To do this, we analyzed 40,000 randomly selected sequences from longer amplicon (602 bp) type I and II KS datasets from lake sediment enrichment cultures (64) using a range of minimum amino acid alignment lengths (5000 of these sequences shown in Table S12). We placed both hit and nonhit sequences in a phylogenetic context within the condensing enzyme superfamily tree (52, 64) and mapped the conserved domains with TREND (65) (Fig. S13). Sequences identified by NaPDoS2 as KSs, regardless of alignment length (30–200aa), fall within the two clades associated with the NaPDoS2 database positive control sequences. Conversely, the sequences that NaPDoS2 did not identify as KSs fell outside of these clades and were associated with off-target, nonketosynthase domains such as AMP-binding domains and multiple phage-related domains. These results confirm that shorter NaPDoS2 alignment length settings can be used to assess polyketide biosynthetic potential from amplicon datasets and highlight the value of verifying detection and classification accuracy using phylogenetic approaches and conserved domain architectures.

## Conclusions

While several tools can detect and classify the gene clusters associated with natural product biosynthesis (1, 6), NaPDoS2 employs KS and C domains as sequence tags to predict biosynthetic potential. This approach makes it well-suited for nonclustered or incomplete BGCs, amplicons, and eukaryotic genomes where other tools are less effective. The NaPDoS2 update features an expanded KS database and classification scheme that better reflects the broader taxonomic and functional diversity now recognized among type I and II PKSs. It provides a single workflow to detect and classify KS domains from diverse biological origins including bacteria, fungi, and other eukaryotes and to distinguish among those involved in fatty acid and specialized metabolite production. Updates to workflow efficiency can now accommodate the larger genomic, metagenomic, and amplicon datasets achievable with next-generation sequencing. NaPDoS2 provides a rapid method to identify microorganisms or environments with the potential to yield rare classes of compounds, such as those produced by noniterative type II PKSs (13). It can be used to prioritize samples for cultivation and to identify potentially new biosynthetic mechanisms when sequences are phylogenetically distinct from those previously characterized, as recently demonstrated for the standalone KS (*salC*) that functions as an aldolase/β-lactone synthase in salinosporamide A biosynthesis (66). The NaPDoS2 upgrades expand on the PKS diversity that can be detected with this tool and provide a method to quickly assess biosynthetic potential in a manner that facilitates more targeted approaches to natural product discovery.

## Experimental procedures

### Sequence database expansion

Experimentally validated PKS BGCs were selected from MIBiG (https://mibig.secondarymetabolites.org/) and published reports. Sequences were extracted from GenBank (https://www.ncbi.nlm.nih.gov/genbank/) and KS domains identified using annotations from both published literature and MIBiG. *In silico* predictions were made using the NRPS/PKS prediction tool (67). KS domains were further annotated (*e.g.*, type II noniterative) according to experimentally verified functions and information derived from the associated gene or BGC. Metadata including BGC name, BGC type (*e.g.*, PKS, NRPS), MIBiG accession number, PubMed reference, source organism, and example product name and structure were recorded for each BGC. The complete NaPDoS2 amino acid sequence database (KS and C domains in FASTA format), BGC table, and domain metadata can be downloaded from the NaPDoS2 website.

### KS sequence alignment, phylogenetic analysis, and classification

Phylogenies generated from the 1877 KS database sequences (1417 new and 460 existing) were used to establish class and subclass assignments based on correspondence between functional annotation and tree topology. Sequences were aligned using MAFFT (v 7.017) (68) as implemented in Geneious (v 6.1.8) using the FFT-NS-i × 1000 alignment algorithm, BLOSUM62 scoring matrix, and defaults for both gap open penalty and offset value (1.53 and 0.123, respectively). The alignments were trimmed, exported to PHYLIP, and phylogenies generated using the PhyML online tool (http://www.atgc-montpellier.fr/phyml/) with smart model selection enabled (69).

The type II aromatic PKS subclasses were established using a concatenated alignment of the KSα and KSβ subunits and phylogenies generated using the methods described above. Subclasses were delineated based on phylogenetic groupings and features of the core polyketide structure including the length of the poly-β-ketoacyl intermediate, the carbon position of the first ring cyclization, and the type of starter unit. The accuracy of select NaPDoS2 KS classifications from the use case analyses were assessed by analyzing the associated contig with antiSMASH 6.0 (1), the NRPS/PKS prediction tool (67), and transATor (7) (Fig. S6).

### KS reference trees

A subset of 414 KS sequences representative of all class and subclass assignments were chosen to generate a KS reference tree (Figs. 1 and S7). Sequences were aligned using MAFFT (68) (v 7.407, FFT-NS-i × 1000 alignment algorithm, BLOSUM62 scoring matrix, default gap open penalty = 1.53, and offset value = 0.123) and trimmed using trimAI (70) (1.4.1 with automatic configuration). The trimmed alignment was used to estimate a maximum likelihood tree using FastTree (71) (v 2.1.11, LG+G model of evolution) with 1000 bootstraps. Booster (72) (v 0.3.1) was used to estimate support as transfer bootstrap expectation values. These programs were implemented in ngphylogeny.fr (71) and run locally as a docker image. Two type II KS phylogenies were generated following the same steps. One comprising all 201 type II KS sequences in the database, eight FAS sequences, and three thiolase outgroup sequences (Figs. 3 and S8). The second used concatenated KSα and KSβ subunits from 59 type II aromatic BGCs (Figs. 4 and S9). Trees were visualized using TreeViewer (https://treeviewer.org/) and annotations added using Adobe Illustrator.

### The NaPDoS2 workflow

Database sequences and associated metadata are stored in a back-end MySQL database linked to the NaPDoS2 web portal through CGI-scripting as previously described (8). The transeq tool from the EMBOSS package (v 6.6) is used for 6-frame translations of nucleic acid queries (73). BLAST queries of amino acid sequences are performed using DIAMOND v 0.9.29 (19). Multiple sequence alignments are obtained with MUSCLE v 3.8 (74) using the profile alignment feature to merge query sequences with previously aligned database sequences. Phylogenetic trees are constructed from trimmed amino acid sequences using FastTree v 2.2.1 (75). Graphical tree depictions are generated using Newick Utilities v 1.5.0.

### Performance testing

The 1877 full-length amino acid sequences in the NaPDoS2 KS database were clustered at 50% identity using CD-HIT v 4.7 (76), yielding 213 nonredundant positive controls. Using the CD-Search (77) function of the curated NCBI Conserved Domain Database (78), negative controls were selected from subfamilies within the condensing enzyme superfamily (cl09938) that are functionally related to type I and II KS domains but phylogentically distinct from the positive control sequences. An additional 49 sequences (52) and 14 KSs from type III PKSs in the MIBiG 2.0 repository (22) were added for a total of 697 sequences (Table S3), which were also clustered at 50% identity using CD-HIT to obtain 308 nonredundant negative controls.

### Cross-validation and receiver operating characteristic curves

Domain detection sensitivity and specificity were determined using BLASTP searches of full-length positive and negative control sequences against the NaPDoS and NaPDoS2 databases, excluding self-matches, to generate leave-one-out cross-validation values. Receiver operating characteristic (ROC) curves were constructed and AUC values calculated from these results using easyROC v 1.3 (79). EasyROC data output tables were used to identify potential cutoff points based on the most restrictive cross-validation e-value at which 100% of true positives were detected, thus maximizing sensitivity with the minimum possible number of false positives.

### KS detection and classification accuracy

A custom perl script (sequence_subdivider.pl, available at https://github.com/spodell/NaPDoS2_website) was used to subdivide the 213 positive control KS sequences into test sets containing overlapping subsequences of 30, 50, 100, or 200 amino acids, each offset by a 10 amino acid sliding window start site. These size-selected test sets, which contained 8555, 8129, 7064, and 4934 subsequences, respectively, were analyzed using the NaPDoS2 workflow to assess the effects of query size on classification accuracy. Accuracy evaluations for each test set were based on the percentage of subsequences whose best nonself, BLASTP match had the same NaPDoS2 classification as the original, full-length sequence from which it was derived.

### Application use cases

Accession information for all sequences and datasets analyzed is provided in Table S3. All analyses used the following default settings unless noted otherwise: NaPDoS version 1: domain detection: Hidden Markov Model 1e-5, 200aa minimum alignment length, pathway assignment: e-value cutoff of 1e-5. NaPDoS2: e-value cutoff 1e-8 and 200aa minimum alignment length. Sequence files containing >500,000 sequences or larger than 500 MB were split into smaller subunits using a custom perl script (serialize_seqs.pl, available at https://github.com/spodell/NaPDoS2_website).

### Genomes and metagenomes

*Salinispora* genome protein sequences (53) downloaded from NCBI and JGI IMG/MER (80) were concatenated into a single FASTA file for NaPDoS2 analysis. Fungal genome protein sequences were downloaded from NCBI; fungal PKS BGCs were extracted from the MIBiG 2.0 repository using the query “Kingdom: Fungi AND BGC type: pks”. Coding sequences and predicted proteins for *E. chlorotica* were downloaded from NCBI (56). Trimmed *E. chlorotica* KS sequences generated by NaPDoS2 were aligned with previously published EcPKS1, EcPKS2, and EcFAS sequences (57) and used to construct a phylogenetic tree with the closest NaPDoS2 database hits. Marine sediment metagenomes were selected from the Paired Omics Data Platform (60) and downloaded from NCBI SRA.

### Amplicons

KS amplicon sequences were analyzed using minimum alignment lengths of 50aa in NaPDoS2 unless otherwise noted. Sequence accession and dataset references are listed in Table S3. Random subsets of query sequences were obtained using custom perl scripts (get_seq_info.pl, randomize_lines.pl, serialize_large_list.pl, and getseq_multiple.pl, available at https://github.com/spodell/NaPDoS2_website).

## Data availability

Relevant alignment files, phylogenetic tree files, webtool documentation file, example tutorials, sequence files, and other supporting information can be found on the corresponding OSF project page: https://osf.io/uzhcp/. The code used to construct version two of the Natural Product Domain Seeker website can be found on the corresponding Github repository: https://github.com/spodell/NaPDoS2_website. All accession information and dataset references can be found in Table S3 (separate Microsoft Excel file).

## Supporting information

Additional figures and tables referred to in the text (1, 7, 8, 22, 52, 53, 56, 57, 60, 61, 62, 63, 64, 65, 67, 74, 75, 78, 81, 82, 83, 84, 85).

Figs. S1–S13: NaPDoS2 workflow and website screenshots, updated database statistics and classification validation, positive/negative control sequence selection, classification category accuracy validation, phylogenetic context of amplicon dataset analysis, and expanded KS phylogenetic trees.

Tables S1–S12: NaPDoS2 dataset analysis speed comparisons, classification category taxa, application use case analyses of genome, metagenome, and amplicon datasets, and accession information for all analyzed sequences and datasets. Table S3 (Microsoft Excel file): all dataset and sequence accession information.

## Conflict of interest

The authors declare that they have no conflicts of interest with the contents of this article.
