# Supplementary material for: The Natural Product Domain Seeker version 2 (NaPDoS2) webtool relates ketosynthase phylogeny to biosynthetic function
Source: J Biol Chem. 2022 Sep 12;298(10):102480. doi: 10.1016/j.jbc.2022.102480 (PMC9582728; doi:10.1016/j.jbc.2022.102480)
Supplement: Supporting Information [file mmc1.pdf]

***Supporting Information for:***

**The Natural Product Domain Seeker version 2 (NaPDoS2) webtool relates ketosynthase phylogeny to biosynthetic function**

Leesa J Klau[1,2,§], Sheila Podell[1,§], Kaitlin E Creamer[1,§], Alyssa M Demko[1,5], Hans W Singh[1], Eric E Allen[1,3], Bradley S Moore[1,4], Nadine Ziemert[1,6], Anne Catrin Letzel[1], Paul R Jensen[1,*]

[1] Center for Marine Biotechnology and Biomedicine, Scripps Institution of Oceanography, University of California San Diego, La Jolla, CA 92037, United States

[2] Department of Biotechnology and Food Science, Norwegian University of Science and Technology (NTNU), Trondheim, Norway

[3] Marine Biology Research Division, Scripps Institution of Oceanography, University of California San Diego, La Jolla, CA 92037, United States

[4] Skaggs School of Pharmacy and Pharmaceutical Sciences, University of California San Diego, La Jolla, CA 92037, United States

* Email: pjensen@ucsd.edu

[§] L.J.K., S.P., and K.E.C. contributed equally to this paper.

Present addresses:

[5] A.M.D.: Smithsonian Marine Station, Fort Pierce, FL 34949, United States

[6] N.Z.: Interfaculty Institute of Microbiology and Infection Medicine, Institute for Bioinformatics and Medical Informatics (IBMI), University of Tübingen, Auf der Morgenstelle 28, 72076 Tübingen, Germany. German Centre for Infection Research (DZIF), Partner Site Tübingen, Germany

***Supporting Information Table of Contents:***

**Figure S1.** NaPDoS2 bioinformatic pipeline……..………...………………….…………pg.S-3

**Figure S2.** Featured webtool updates….……………………………………......…..pg.S-4

**Figure S3.** NaPDoS2 workflow and analysis roadmap..…………………….......…..pg.S-6

**Figure S4.** Comparison of the expanded NaPDoS2 database…………………...….pg.S-8

**Figure S5.** NaPDoS2 classification overview……………………..............………........pg.S-9

**Figure S6.** Verification of NaPDoS2 KS domain classifications.............………........pg.S-12

**Figure S7.** Maximum likelihood KS phylogeny..……………..…………………......….pg.S-14

**Figure S8.** Maximum likelihood type II KS phylogeny………...………….............….pg.S-16

**Figure S9.** Maximum likelihood type II aromatic KSα and KSβ phylogeny …........pg.S-18

**Figure S10.** Negative control KS sequence selection…………………………..……..pg.S-20

**Figure S11.** Effect of query size on KS detection and accuracy……........……….pg.S-22

**Figure S12.** KS domain sequence diversity....……………………….…………pg.S-24
**Figure S13.** Amplicon detection accuracy……..…………….……………...……pg.S-26

**Table S1.** Processing times for NaPDoS release (V1) versus NaPDoS2 (V2)…..pg.S-28
**Table S2.** NaPDoS2 database summary………………………………..……..pg.S-29
**Table S3.** Accession numbers and dataset references (Excel file)………………..pg.S-31
**Table S4.** *Salinispora* spp. type II KS domains………...…………………………...pg.S-32
**Table S5.** Complete list of *Salinispora* spp. KS domains identified by NaPDoS2.pg.S-34
**Table S6.** KS domains identified in 27 fungal genomes by NaPDoS2..…….….…pg.S-37
**Table S7.** KS detection using NaPDoS versions 1 and 2…………………………..pg.S-39
**Table S8.** NaPDoS2 analysis of the *Elysia chlorotica* genome.…………….………pg.S-41
**Table S9.** Moorea sediment metagenomes analyzed with NaPDoS2………..…..pg.S-43
**Table S10.** NaPDoS2 analysis of an eSNaPD v2.0 dataset. ………………….…..pg.S-45
**Table S11.** NaPDoS2 analysis of amplicon datasets from Borsetto *et al.* 2019....pg.S-47
**Table S12.** NaPDoS2 analysis of amplicon sequences from Elfeki *et al.* 2018….pg.S-49

**References……………………………………………………………………...pg.S-51**

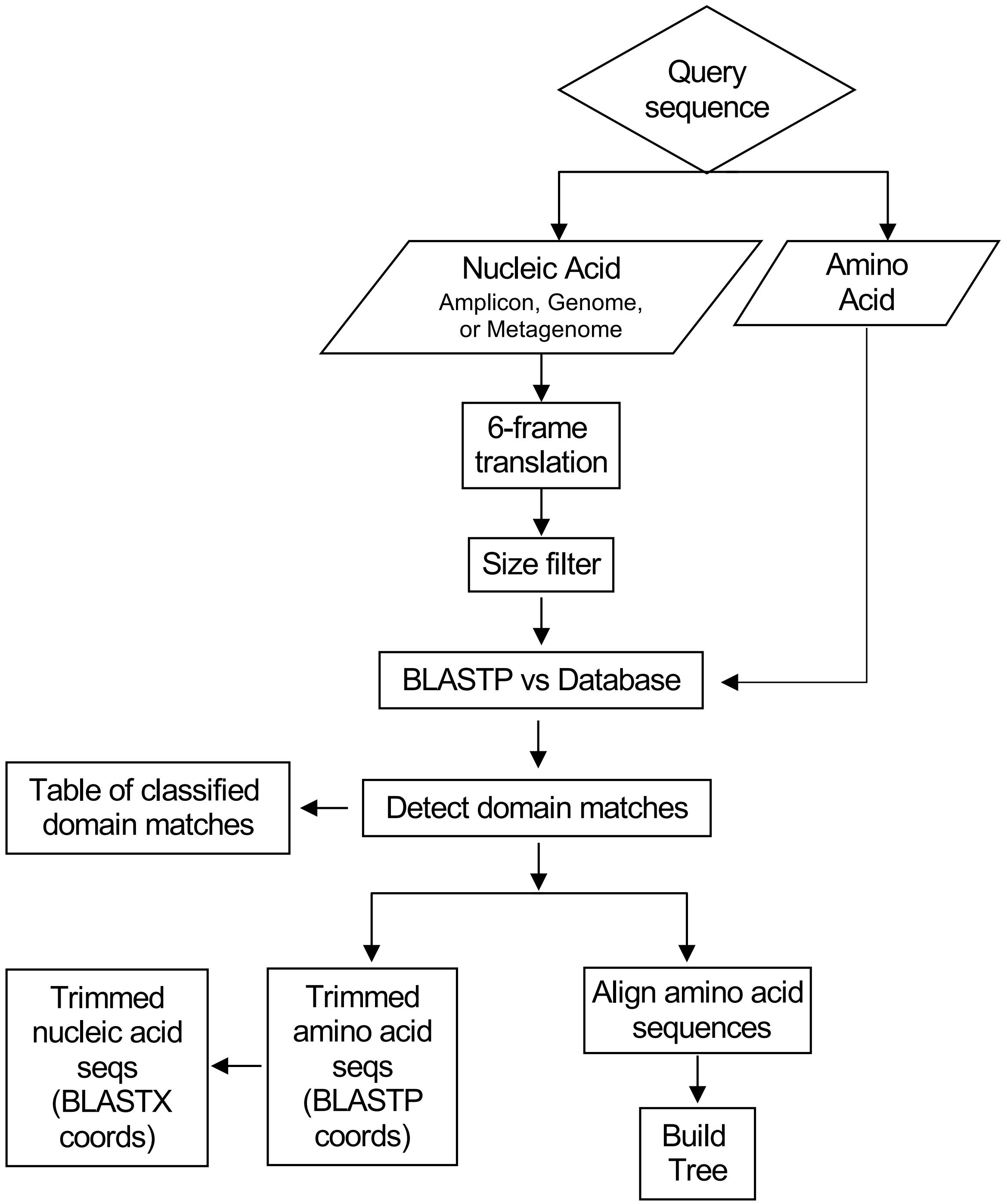


**Figure S1. NaPDoS2 bioinformatic pipeline.** Translated nucleotide and protein query sequences are compared to an internal database of KS and C domains using BLASTP. Detailed descriptions of individual steps can be found in the GitHub repository site: https://github.com/spodell/NaPDoS2_website.

**Figure S2.**

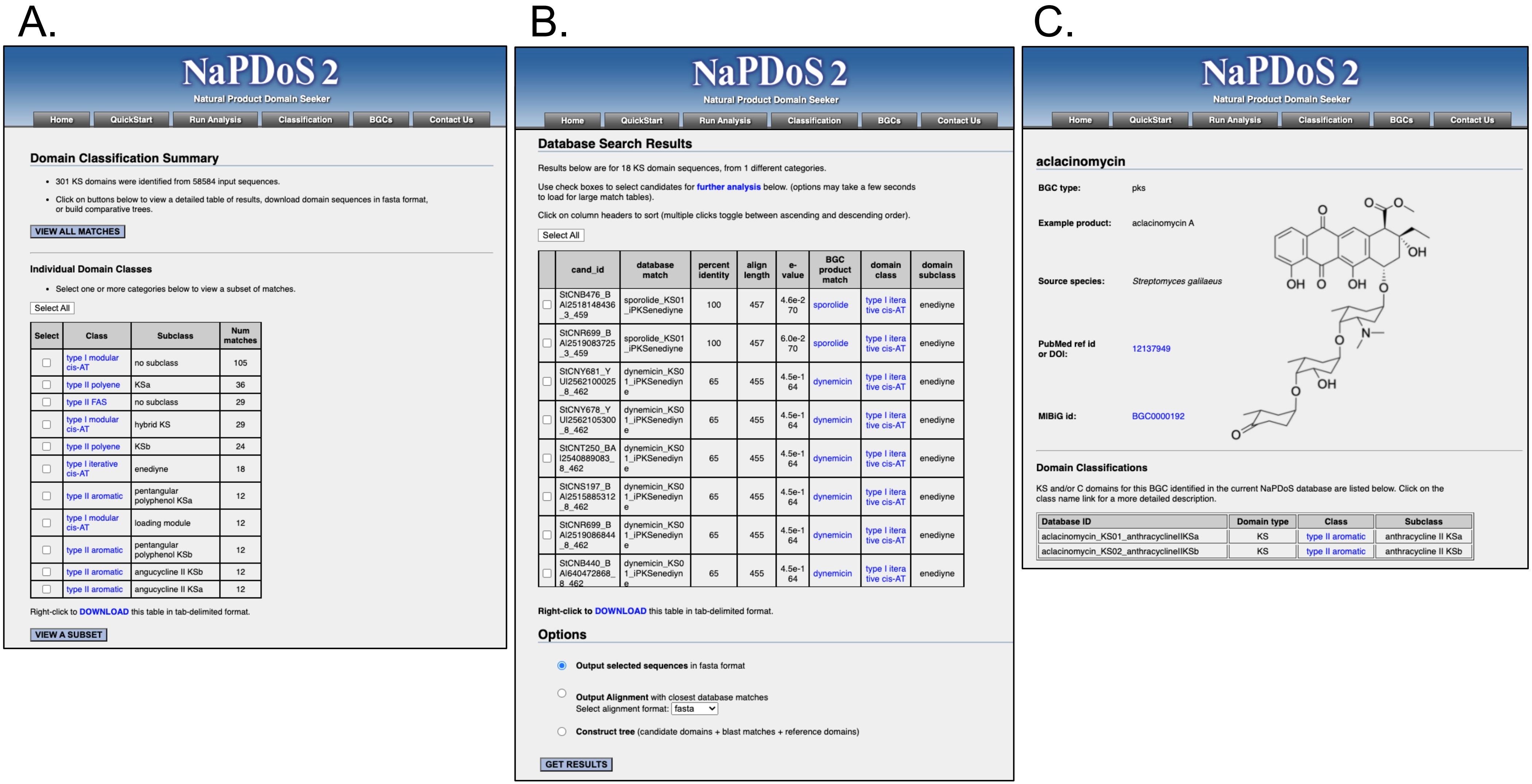
A.
NaPDoS 2
Natural Product Domain Seeker
Home
QuickStart
Run Analysis
Classification
BGCs
Contact Us
Domain Classification Summary
301 KS domains were identified from 58584 input sequences.
Click on buttons below to view a detailed table of results, download domain sequences in fasta format, or build comparative trees.
VIEW ALL MATCHES
Individual Domain Classes
Select one or more categories below to view a subset of matches.
Select All
Select | Class | Subclass | Num matches
type I modular cis-AT | no subclass | 105
type II polyene | KSa | 36
type II FAS | no subclass | 29
type I modular cis-AT | hybrid KS | 29
type II polyene | KSb | 24
type I iterative cis-AT | enediyne | 18
type II aromatic | pentangular polyphenol KSa | 12
type I modular cis-AT | loading module | 12
type II aromatic | pentangular polyphenol KSb | 12
type II aromatic | angucycline II KSb | 12
type II aromatic | angucycline II KSa | 12
Right-click to DOWNLOAD this table in tab-delimited format.
VIEW A SUBSET
B.
NaPDoS 2
Natural Product Domain Seeker
Home
QuickStart
Run Analysis
Classification
BGCs
Contact Us
Database Search Results
Results below are for 18 KS domain sequences, from 1 different categories.
Use check boxes to select candidates for further analysis below. (options may take a few seconds to load for large match tables).
Click on column headers to sort (multiple clicks toggle between ascending and descending order).
Select All
cand_id | database match | percent identity | align length | e-value | BGC product match | domain class | domain subclass
StCNB476_BAI2518148436_3_459 | sporolide_KS01_iPKSenediyne | 100 | 457 | 4.6e-270 | sporolide | type I iterative cis-AT | enediyne
StCNR699_BAI2519083725_3_459 | sporolide_KS01_iPKSenediyne | 100 | 457 | 6.0e-270 | sporolide | type I iterative cis-AT | enediyne
StCNY681_YUI2562100025_8_462 | dynemicin_KS01_iPKSenediyne | 65 | 455 | 4.5e-164 | dynemicin | type I iterative cis-AT | enediyne
StCNY678_YUI2562105300_8_462 | dynemicin_KS01_iPKSenediyne | 65 | 455 | 4.5e-164 | dynemicin | type I iterative cis-AT | enediyne
StCNT250_BAI2540889083_8_462 | dynemicin_KS01_iPKSenediyne | 65 | 455 | 4.5e-164 | dynemicin | type I iterative cis-AT | enediyne
StCNS197_BAI2515885312_8_462 | dynemicin_KS01_iPKSenediyne | 65 | 455 | 4.5e-164 | dynemicin | type I iterative cis-AT | enediyne
StCNR699_BAI2519086844_8_462 | dynemicin_KS01_iPKSenediyne | 65 | 455 | 4.5e-164 | dynemicin | type I iterative cis-AT | enediyne
StCNB440_BAI640472868_8_462 | dynemicin_KS01_iPKSenediyne | 65 | 455 | 4.5e-164 | dynemicin | type I iterative cis-AT | enediyne
Right-click to DOWNLOAD this table in tab-delimited format.
Options
Output selected sequences in fasta format
Output Alignment with closest database matches
Select alignment format: fasta
Construct tree (candidate domains + blast matches + reference domains)
GET RESULTS
C.
NaPDoS 2
Natural Product Domain Seeker
Home
QuickStart
Run Analysis
Classification
BGCs
Contact Us
aclacinomycin
BGC type: pks
Example product: aclacinomycin A
Source species: Streptomyces galilaeus
PubMed ref id or DOI: 12137949
MIBiG id: BGC0000192
Domain Classifications
KS and/or C domains for this BGC identified in the current NaPDoS database are listed below. Click on the class name link for a more detailed description.
Database ID | Domain type | Class | Subclass
aclacinomycin_KS01_anthracyclineIIKSa | KS | type II aromatic | anthracycline II KSa
aclacinomycin_KS02_anthracyclineIIKSb | KS | type II aromatic | anthracycline II KSb

**Figure S2**. **Featured webtool updates**. A). Domain classification summary page. In this example, 301 KS domains were detected in twelve bacterial genomes (58,584 protein sequences). The number of domains detected in each class and subclass is indicated. B). Database search results page. Expanded view of the 18 type I iterative *cis*-AT enediyne domains from the search shown in (A).  C). BGC page. Clicking on the BGC product match hyperlink from the Database Search Results in panel (B) provides the compound structure and details about the associated BGC and all corresponding KS classifications (C domains to be updated in a later release).

**Figure S3.**

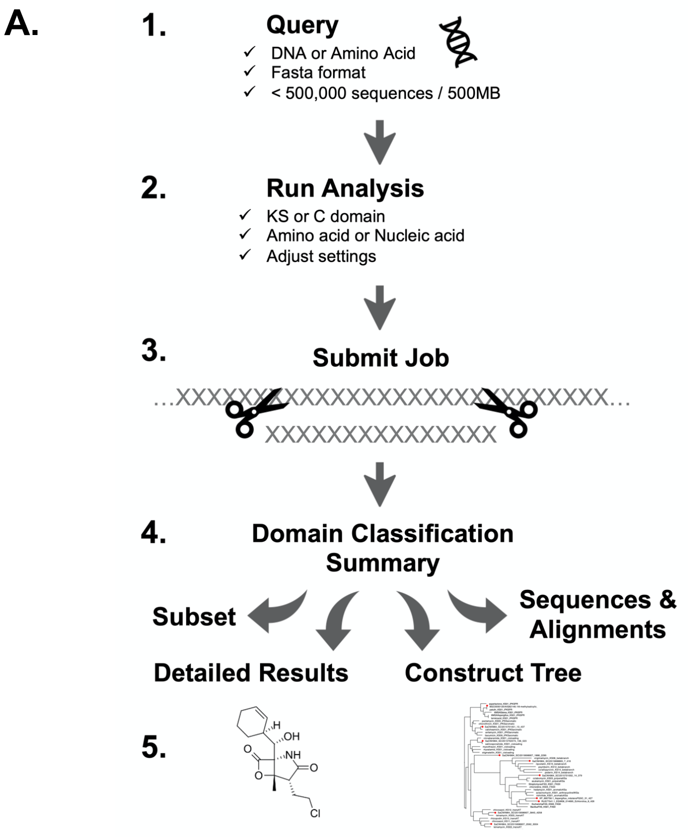

A.
1.
Query
DNA or Amino Acid
Fasta format
< 500,000 sequences / 500MB
2.
Run Analysis
KS or C domain
Amino acid or Nucleic acid
Adjust settings
3.
Submit Job
…XXXXXXXXXXXXXXXXXXXXXXXXXXXXXXXXXX…
XXXXXXXXXXXXXXX
4.
Domain Classification Summary
Subset
Sequences & Alignments
Detailed Results
Construct Tree
5.


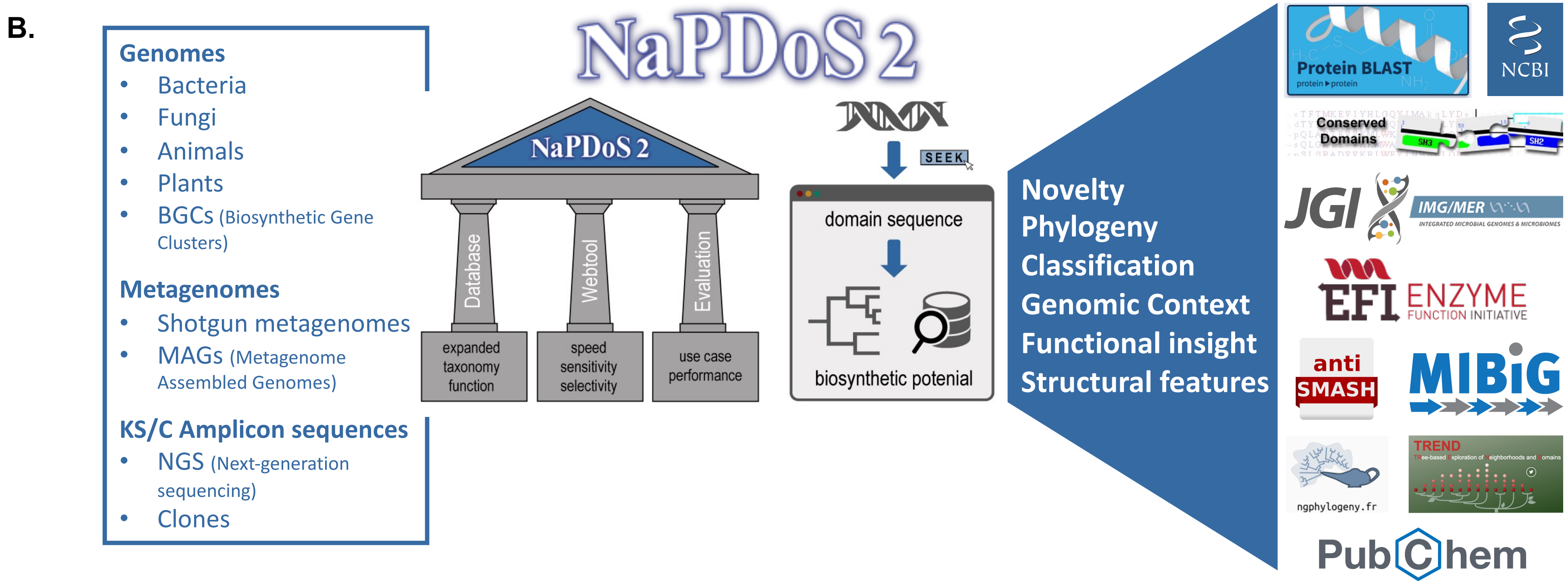

B.
Genomes
Bacteria
Fungi
Animals
Plants
BGCs (Biosynthetic Gene Clusters)
Metagenomes
Shotgun metagenomes
MAGs (Metagenome Assembled Genomes)
KS/C Amplicon sequences
NGS (Next-generation sequencing)
Clones
NaPDoS 2
NaPDoS 2
Database
Webtool
Evaluation
expanded taxonomy function
speed sensitivity selectivity
use case performance
SEEK
domain sequence
biosynthetic potenial
Novelty
Phylogeny
Classification
Genomic Context
Functional insight
Structural features
Protein BLAST
protein ▸ protein
NCBI
Conserved Domains
JGI
IMG/MER
EFI ENZYME FUNCTION INITIATIVE
anti SMASH
MIBiG
ngphylogeny.fr
TREND
PubChem

**Figure S3. NaPDoS2 workflow and analysis roadmap.** A) The NaPDoS2 user workflow consists of submitting a query sequence, selecting the type of analysis to run, submitting the job, and deciding what output to view or analyses to complete. B) A roadmap for the use of NaPDos2 starts with genomic, metagenomic, or KS/C domain amplicon sequences derived from a variety of sources. The expanded database and webtool improvements provide important analytical upgrades while extensive use testing demonstrates the applications of NaPDoS2 to assess biosynthetic potential by detecting and classifying KS and C domain sequences. NaPDoS2 output can be further analyzed using a variety of tools to assess novelty, phylogeny, classification, genomic context, function, and small molecule structural features. Detailed, step-by-step tutorial examples can be found in the downloadable "Documentation" PDF linked on the NaPDoS2 webpage.

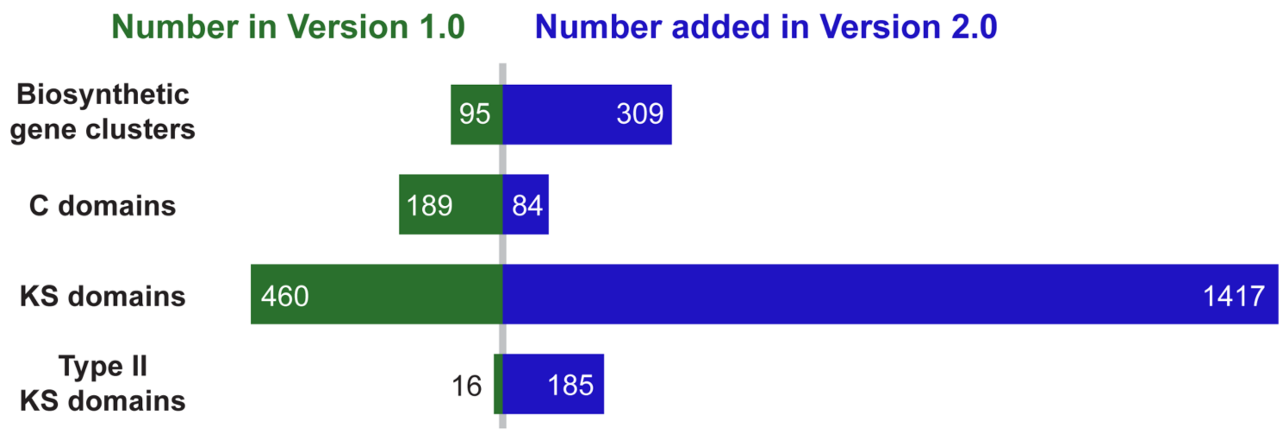


**Figure S4. Comparison of the expanded NaPDoS2 database with the original NaPDoS version 1 release[1].** The NaPDoS2 reference database contains 273 C domains and 1,877 KS domains from a total of 404 BGCs.

**Figure S5.**

# NaPDoS 2

Natural Product Domain Seeker

Home | QuickStart | Run Analysis | Classification | BGCs | Contact Us

## Classification Overview

The NaPDoS2 classification scheme is summarized below. For more detailed descriptions of the KS and C domain categories and relevant literature see the downloadable DOCUMENTATION file.

Database sequences have been given class and subclass designations based on their phylogenetic clade topology and established functions in their respective PKS or NRPS genes. Note that individual domains in the same gene may be classified differently. These classifications can provide insight into:

- product structural features (e.g., PUFAs, enediynes, aromatic polyketides)
- gene architecture (e.g., cis- or trans-AT)
- taxonomic groups in which the sequence resides (e.g., bacteria, fungi, protist, metazoa).

Query sequences are assigned classifications according to their the top NaPDoS2 BLAST match, providing insight into the biosynthetic potential of the sample. The abbreviations used in the database, reference tree, and NaPDoS2 output are given in parentheses below.

### KS Domains

Delineated into three primary groups: FAS, type I PKS, and type II PKS.

#### 1. Fatty Acid Synthase (FAS)

**Class: Type I FAS**
Large multifunctional proteins responsible for fatty acid biosynthesis.

- **Subclass: Bacteria and fungi (bfFASI)**
  Observed in bacteria and fungi.
- **Subclass: Metazoa (MetazoaFASI)**
  Observed in the phyla Chordata and Nematoda
- **Subclass: Protist (ProtistFASI)**
  Observed in the phylum Apicomplexa (Alveolata)

**Class: Type II FAS (FASII)**
Discrete, monofunctional proteins responsible for fatty acid biosynthesis.

#### 2. Type I

Canonical type I PKSs containing KS, AT, and ACP domains and functioning in an assembly-line fashion.

**Class: Modular cis-AT (cisAT)**
Canonical type I PKSs containing KS, AT, and ACP domains and functioning in an assembly-line fashion.

- **Subclass: Olefin synthase (cisOLS)**
  Associated with the biosynthesis of a terminal olefin.
- **Subclass: Loading module (cisloading)**
  KS in the first module of cis-AT modular PKS in which the catalytic cysteine has been replaced with glutamine (sometimes called KSQ).
- **Subclass: Hybrid (cisHybridKS)**
  Downstream of a peptidyl carrier protein (PCP) domain. Catalyzes the condensation of an acyl group on a PCP-tethered intermediate.
- **Subclass: Tandem ECH (cistandemECH)**
  Found in modules immediately downstream of beta-branching cassettes (gene cassettes involved in the introduction of a beta-branch). Contain a *cis*-acting ECH domain that performs the final decarboxylation to produce an unsaturated beta-branch.

**Class: Iterative cis-AT (iPKS)**
Cis-AT type I PKSs that function iteratively, observed in bacteria and fungi.

- **Subclass: Polyunsaturated fatty acid (iPKSPUFA)**
  Produce long chain fatty acids that contain multiple cis double bonds.
- **Subclass: Enediyne (iPKSenediyne)**
  Produce nine- or ten-membered rings that contain a conjugated alkyne-alkene-alkyne moiety.
- **Subclass: Aromatic (iPKSaromatic)**
  Produce simple aromatic compounds that usually consist of mono- or bicyclic rings.
- **Subclass: Polycyclic tetramate macrolactam-like (iPKSPTM)**
  Produce compounds usually consisting of a tetramic acid moiety and 2-3 rings fused to a macrolactam.
- **Subclass: Non-reducing (iPKSNR)**
  Associated with PKSs that lack all KR, DH, and ER domains. Produce mono- or polycyclic aromatic polyketides from poly-beta-keto chains. Observed in fungi.
- **Subclass: Partially reducing (iPKSPR)**
  Associated with PKSs that lack some KR, DH, and ER domains. Produce simple mono- or bicyclic aromatic compounds similar to the products of bacterial aromatic iPKSs. Observed in fungi.
- **Subclass: Highly reducing (iPKSHR)**
  Associated with PKSs that possess all KR, DH, and ER domains. Produce linear and cyclic non-aromatic compounds. Observed in fungi.

**Class: trans-AT (transAT)**
Modular, assembly line PKS in which the AT domain(s) are freestanding as opposed to occuring in the module.

- **Subclass: B domain (transBdomain)**
  KS domains that occur together with a branching (B) domain and facilitate the formation of a beta branch.
- **Subclass: Hybrid (transHybridKS)**
  Similar to cisHybridKSs (see above) except the AT domain occurs in trans.
- **Subclass: Hybrid non-elongating KS (transHybridKS0)**
  Non-elongating KS domains (KS0) in trans-AT modules that follow an NRPS module.

**Class: Metazoa (MetazoaPKS)**
Type I KS domains detected in metazoa.

**Class: Protist (ProtistPKS)**
Type I KS domains detected in protists.

**Figure S5.**

**3. Type II**

Discrete, monofunctional proteins.

**Class: Aromatic (aromaticKSa or aromaticKSb)**
Heterodimers that consist of alpha and beta subunits and produce polycyclic aromatic compounds through the iterative decarboxylative condensation of malonyl-CoA extender units onto an acyl starting unit.

- **Subclass: angucycline-derived I (angucyclineIKSa or angucyclineIKSb)**
  Compounds contain or were derived from an angular tetracyclic structure comprising a benzanthracene moiety. Most frequently initiated with acetyl-CoA starting unit.
- **Subclass: angucycline-derived II (angucyclineIIKSa or angucyclineIIKSb)**
  Distinguished from angucycline-derived I by initiation with a methylmalonyl-CoA starting unit.
- **Subclass: anthracycline-derived I (anthracyclineIKSa or anthracyclineIKSb)**
  Compounds possess a linear tetracyclic core derived from 7,8,9,10-tetrahydro-5,12-naphtacenoquinones. Initiated with acetyl-CoA starting unit.
- **Subclass: anthracycline-derived II (anthracyclineIIKSa or anthracyclineIIKSb)**
  Distinguished from anthracycline-derived I by initiation with methylmalonyl-CoA starting unit.
- **Subclass: isochromanequinone-derived (isochromanequinoneKSa or isochromanequinoneKSb).**
  Compounds with a linear tricyclic core structure containing isochromane and quinone moieties often forming dimers.
- **Subclass: pentangular polyphenol-derived (pentangularpolyphenolKSa or pentangularpolyphenolKSb)**
  Produce long-chain polyphenols that form angular polycyclic core structures.
- **Subclass: tetracenomycin-derived (tetracenomycinKSa or tetracenomycinKSb)**
  Produce linear tetracyclic decaketide core structures resulting from nine elongations of an acetyl-CoA starting unit.
- **Subclass: tetracycline-derived (tetracyclineKSa) or (tetracyclineKSb)**
  Produce compounds with a tetracyclic ring structure characterized by a carboxamido moiety resulting from a malonamyl-CoA starting unit.
- **Subclass: spore pigment (sporepigmentKSa or sporepigmentKSb)**
  Associated with the biosynthesis of streptomycete spore pigments (e.g. whiE in S. coelicolor) although compounds are not well characterized.

**Class: Beta-branching cassettes (betabranch)**
KS domains associated with HMGS cassettes that introduce a beta-branch to a beta-keto group. These stand alone KSs lack the active site cysteine required for condensation and function to decarboxylate ACP-bound malonyl as an early step in beta branch formation

**Class: Polyenes (polyeneKSa or polyeneKSb)**
Iteratively acting KSs that produce reduced, linear polyenes rather than polycyclic aromatic compounds.

**Class: Aryl polyenes (arylpolyeneKSa or arylpolyeneKSb)**
Iteratively acting PKSs that produce polyene chains with an aryl moiety that is often substituted.

**Class: Non-iterative (noniterative)**
Discrete monofunctional proteins that function as an assembly line to produce compounds such as pamamycin and nonactin.

## C Domains

**Class: Starter (starter)**
Typically, the first module of a NRPS usually does not contain a C domain. But, when present, these starter C domains acylate the first amino acid with a fatty acid, polyketide, or other molecule.

**Class: LCL (LCL)**
Catalyzes the formation of a peptide bond between two L-amino acids.

**Class: DCL (DCL)**
Catalyzes the formation of a peptide bond between an L-amino acid and a growing peptide ending with a D-amino acid.

**Class: Cyclization (cyclization)**
Catalyzes both peptide bond formation and the subsequent cyclization of cysteine, serine or threonine residues.

**Class: Epimerization (epimerization)**
Changes the chirality of the last amino acid in the chain from L to D.

**Class: Dual (dual)**
Catalyzes both condensation and epimerization reactions.

**Class: Modified amino acid (modifiedAA)**
Modifies the incorporated amino acid: for example the dehydration of serine to dehydroalanine.

**Class: Hybrid (hybridC)**
Occur in PKS-NRPS BGCs. The condensation domain that occurs immediately downstream of a PKS module; condenses an amino acid to a growing polyketide.

**Class: Condensation (condensation)**
Condensation domains with no known specialized functionality.

Copyright © 2021 Regents of the University of California. All rights reserved.

**Figure S5. NaPDoS2 classification overview**. Class and subclass descriptions as described on the NaPDoS2 website. Additional details and relevant references can be found in the downloadable "Documentation" PDF linked at the top of the webpage.

Figure S6.

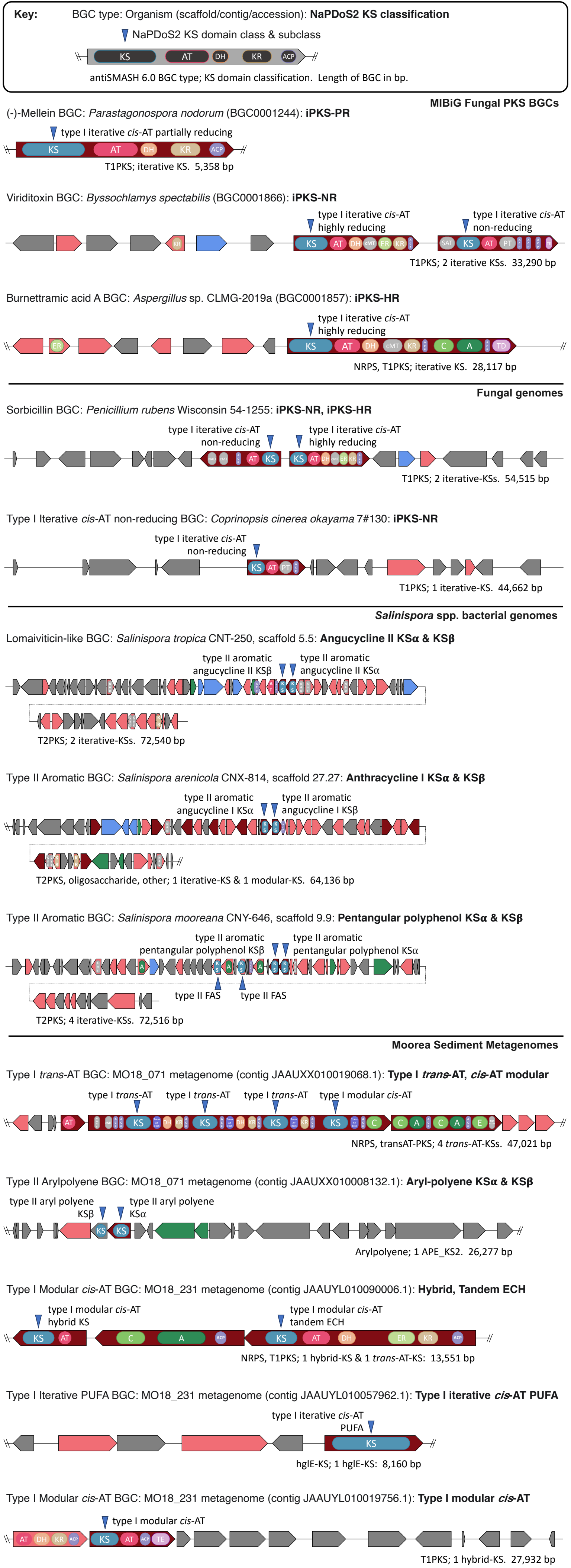
Key:
BGC type: Organism (scaffold/contig/accession): NaPDoS2 KS classification
NaPDoS2 KS domain class & subclass
KS
AT
DH
KR
ACP
antiSMASH 6.0 BGC type; KS domain classification. Length of BGC in bp.
MIBiG Fungal PKS BGCs
(-)-Mellein BGC: Parastagonospora nodorum (BGC0001244): iPKS-PR
type I iterative cis-AT partially reducing
T1PKS; iterative KS. 5,358 bp
Viriditoxin BGC: Byssochlamys spectabilis (BGC0001866): iPKS-NR
type I iterative cis-AT highly reducing
type I iterative cis-AT non-reducing
T1PKS; 2 iterative KSs. 33,290 bp
Burnettramic acid A BGC: Aspergillus sp. CLMG-2019a (BGC0001857): iPKS-HR
type I iterative cis-AT highly reducing
NRPS, T1PKS; iterative KS. 28,117 bp
Fungal genomes
Sorbicillin BGC: Penicillium rubens Wisconsin 54-1255: iPKS-NR, iPKS-HR
type I iterative cis-AT non-reducing
type I iterative cis-AT highly reducing
T1PKS; 2 iterative-KSs. 54,515 bp
Type I Iterative cis-AT non-reducing BGC: Coprinopsis cinerea okayama 7#130: iPKS-NR
type I iterative cis-AT non-reducing
T1PKS; 1 iterative-KS. 44,662 bp
Salinispora spp. bacterial genomes
Lomaiviticin-like BGC: Salinispora tropica CNT-250, scaffold 5.5: Angucycline II KSα & KSβ
type II aromatic angucycline II KSβ
type II aromatic angucycline II KSα
T2PKS; 2 iterative-KSs. 72,540 bp
Type II Aromatic BGC: Salinispora arenicola CNX-814, scaffold 27.27: Anthracycline I KSα & KSβ
type II aromatic angucycline I KSα
type II aromatic angucycline I KSβ
T2PKS, oligosaccharide, other; 1 iterative-KS & 1 modular-KS. 64,136 bp
Type II Aromatic BGC: Salinispora mooreana CNY-646, scaffold 9.9: Pentangular polyphenol KSα & KSβ
type II aromatic pentangular polyphenol KSβ
type II aromatic pentangular polyphenol KSα
type II FAS
type II FAS
T2PKS; 4 iterative-KSs. 72,516 bp
Moorea Sediment Metagenomes
Type I trans-AT BGC: MO18_071 metagenome (contig JAAUXX010019068.1): Type I trans-AT, cis-AT modular
type I trans-AT
type I trans-AT
type I trans-AT
type I modular cis-AT
NRPS, transAT-PKS; 4 trans-AT-KSs. 47,021 bp
Type II Arylpolyene BGC: MO18_071 metagenome (contig JAAUXX010008132.1): Aryl-polyene KSα & KSβ
type II aryl polyene KSβ
type II aryl polyene KSα
Arylpolyene; 1 APE_KS2. 26,277 bp
Type I Modular cis-AT BGC: MO18_231 metagenome (contig JAAUYL010090006.1): Hybrid, Tandem ECH
type I modular cis-AT hybrid KS
type I modular cis-AT tandem ECH
NRPS, T1PKS; 1 hybrid-KS & 1 trans-AT-KS: 13,551 bp
Type I Iterative PUFA BGC: MO18_231 metagenome (contig JAAUYL010057962.1): Type I iterative cis-AT PUFA
type I iterative cis-AT PUFA
hglE-KS; 1 hglE-KS: 8,160 bp
Type I Modular cis-AT BGC: MO18_231 metagenome (contig JAAUYL010019756.1): Type I modular cis-AT
type I modular cis-AT
T1PKS; 1 hybrid-KS. 27,932 bp

**Figure S6**. **Verification of NaPDoS2 KS domain classifications.** Biosynthetic gene cluster context of select KSs detected in the application use case analyses. For each KS, the associated scaffold or contig was extracted and run through antiSMASH 6.0[2] (https://antismash.secondarymetabolites.org/), transATor[3] (https://transator.ethz.ch/), and the "PKS/NRPS Analysis Web-site"[4] (http://nrps.igs.umaryland.edu/). The BGCs were drawn and colored as determined by antiSMASH 6.0 (maroon, core biosynthetic gene; pink, additional biosynthetic gene; blue, transport-related genes; green, regulatory genes, grey, other genes). Domain position and function were drawn and colored according to antiSMASH 6.0[2], transATor[3], and "PKS/NRPS Analysis Web-site"[4] (blue, KS ketosynthase; pink, AT acyl transferase; sand, KR ketoreductase; pale purple ACP Phosphopantetheine acyl carrier protein, ACPS holo-ACP synthase; orange, DH dehydratase; light grey, cMT carbon methyltransferase, FkbH domain, NAD Male sterility protein, AmT aminotransferase, Cyc cyclase, GNAT GNAT domain, TIGR01720 NRPS domain of unknown function; light pink, TE thioesterase, TD Terminal reductase domain; pale green ER enoyl reductase; dark blue, *trans*-AT docking *trans*-acyltransferase docking domain; light green, C condensation domain of NRPS, E epimerization domain; dark green, A adenylation domain). Blue arrows point to KS hits that NaPDoS2 detected and classified in the BGC context; arrows are labeled with the NaPDoS2 KS domain classification. The antiSMASH 6.0[2] BGC type and KS domain classification, followed by the length of the entire BGC (in base pairs) is listed below each BGC, as indicated in the key.

We strategically chose diverse use case analyses for ground-truthing the contextual genomic evidence for KS domain-based classification. NaPDoS2 correctly classified KSs associated with partially reducing ((-)-Mellein), non-reducing (viriditoxin), and highly reducing (burnettramic acid A) fungal BGCs from MIBiG 2.0[5], as confirmed by literature reports and antiSMASH 6.0[2] output. Next, NaPDoS2 correctly classified the KSs in the sorbicillin BGC from *Penicillium rubens* fungal genome, which contains both non-reducing and highly reducing KS domains. NaPDoS2 also identified a type I iterative *cis*-AT non-reducing KS in an orphan BGC in the *Coprinopsis cinerea okayama* basidiomycete genome. Next, NaPDoS2 also correctly identified the KS domains in the lomaiviticin BGC as type II aromatic angucycline II, and detected the KSs associated with two *Salinispora* orphan BGCs as type II aromatic anthracycline I and pentangular polyphenol. Finally, NaPDoS2 correctly classified *trans*-AT, *cis*-AT, aryl-polyene, hybrid, trandem ECH, modular, and PUFA KS domains from metagenomic assemblies based on their respective BGC context. In many cases, the NaPDoS2 classification was more specific than antiSMASH 6.0[2] BGC domain predictions.

# Figure S7.

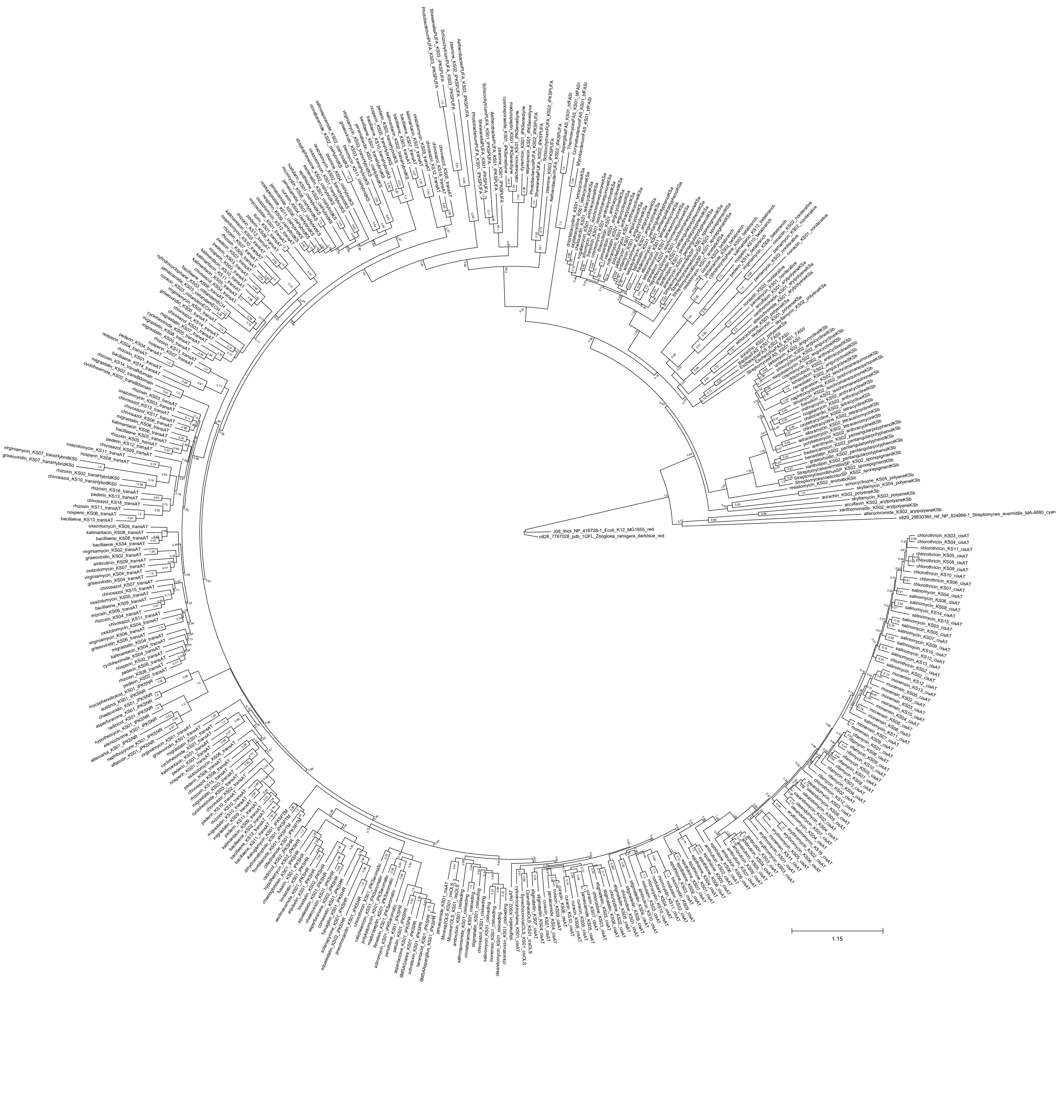

**Figure S7**. **Maximum likelihood KS phylogeny.** Tree includes 414 KS sequences and 3 thiolase sequences as outgroups. The full name of each sequence is listed on the branch tips, which can help link a query match to a specific location in the tree. Bootstrap support is listed for each node. Thiolases from *Escherichia coli* (NP_416728.1) and *Zoogloea ramigera* (1QFL_A) and a SCP-x thiolase from *Streptomyces avermitilis* (NP_824999.1) were used as outgroups.

# Figure S8.

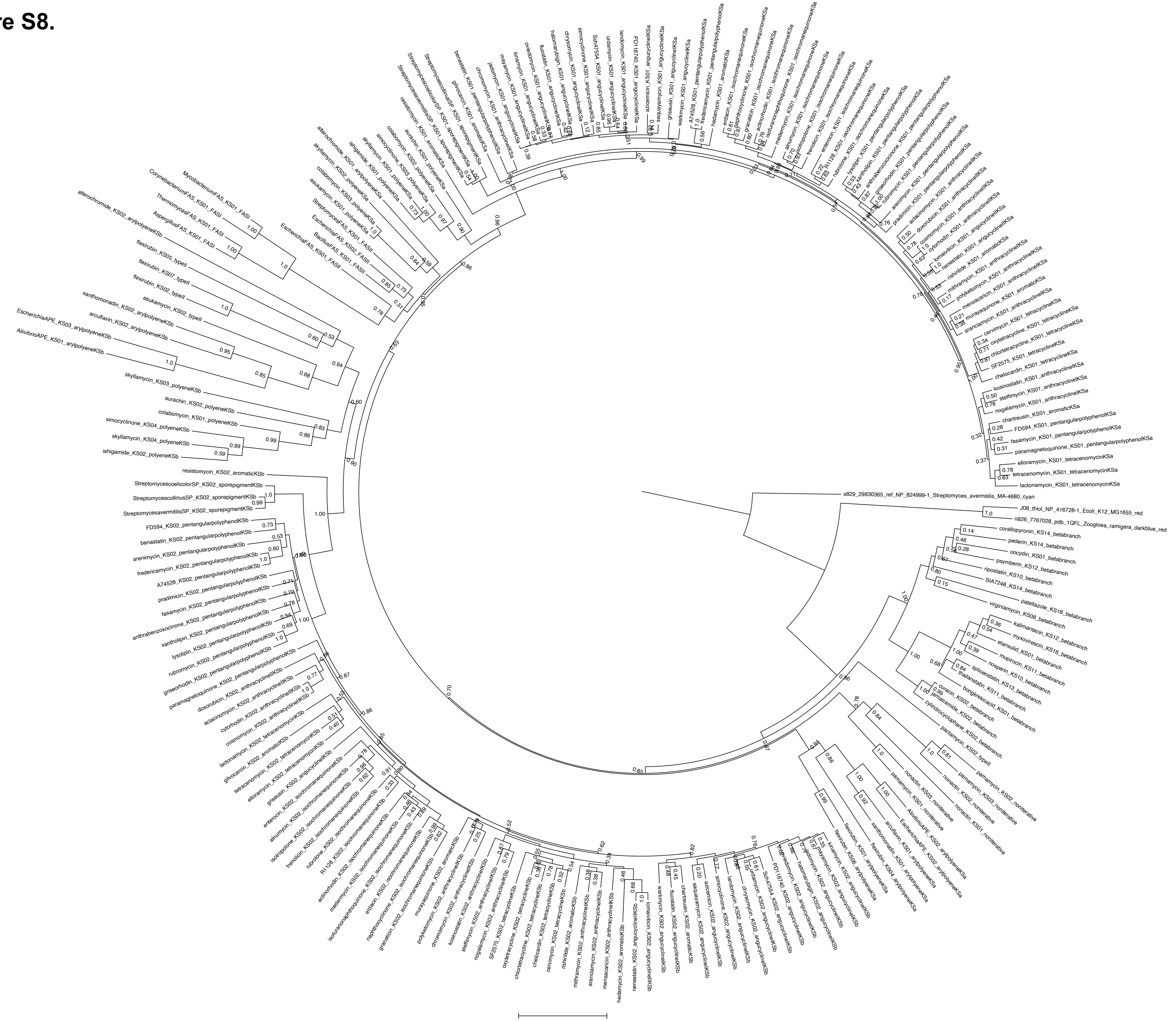

**Figure S8. Maximum likelihood type II KS phylogeny.** Tree includes 201 type II KS sequences, 8 FAS sequences and 3 thiolase sequences as outgroups. The full name of each sequence is listed on the branch tips, which can help link a query match to a specific location in the tree. Bootstrap support is listed for each node. Thiolases from *Escherichia coli* (NP_416728.1) and *Zoogloea ramigera* (1QFL_A) and a SCP-x thiolase from *Streptomyces avermitilis* (NP_824999.1) were used as outgroups.

## Figure S9.

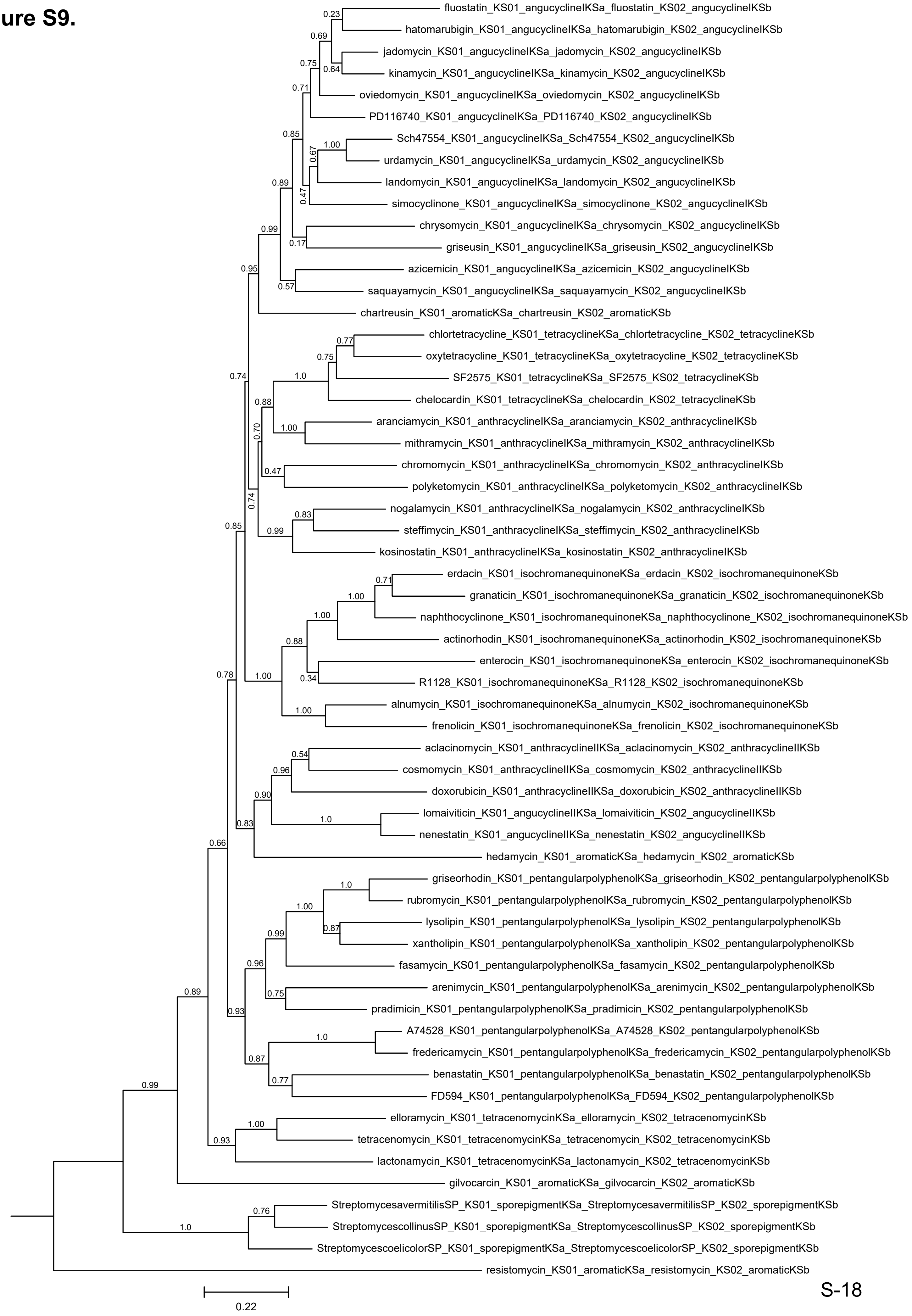

fluostatin_KS01_angucyclineIKSa_fluostatin_KS02_angucyclineIKSb
hatomarubigin_KS01_angucyclineIKSa_hatomarubigin_KS02_angucyclineIKSb
jadomycin_KS01_angucyclineIKSa_jadomycin_KS02_angucyclineIKSb
kinamycin_KS01_angucyclineIKSa_kinamycin_KS02_angucyclineIKSb
oviedomycin_KS01_angucyclineIKSa_oviedomycin_KS02_angucyclineIKSb
PD116740_KS01_angucyclineIKSa_PD116740_KS02_angucyclineIKSb
Sch47554_KS01_angucyclineIKSa_Sch47554_KS02_angucyclineIKSb
urdamycin_KS01_angucyclineIKSa_urdamycin_KS02_angucyclineIKSb
landomycin_KS01_angucyclineIKSa_landomycin_KS02_angucyclineIKSb
simocyclinone_KS01_angucyclineIKSa_simocyclinone_KS02_angucyclineIKSb
chrysomycin_KS01_angucyclineIKSa_chrysomycin_KS02_angucyclineIKSb
griseusin_KS01_angucyclineIKSa_griseusin_KS02_angucyclineIKSb
azicemicin_KS01_angucyclineIKSa_azicemicin_KS02_angucyclineIKSb
saquayamycin_KS01_angucyclineIKSa_saquayamycin_KS02_angucyclineIKSb
chartreusin_KS01_aromaticKSa_chartreusin_KS02_aromaticKSb
chlortetracycline_KS01_tetracyclineKSa_chlortetracycline_KS02_tetracyclineKSb
oxytetracycline_KS01_tetracyclineKSa_oxytetracycline_KS02_tetracyclineKSb
SF2575_KS01_tetracyclineKSa_SF2575_KS02_tetracyclineKSb
chelocardin_KS01_tetracyclineKSa_chelocardin_KS02_tetracyclineKSb
aranciamycin_KS01_anthracyclineIKSa_aranciamycin_KS02_anthracyclineIKSb
mithramycin_KS01_anthracyclineIKSa_mithramycin_KS02_anthracyclineIKSb
chromomycin_KS01_anthracyclineIKSa_chromomycin_KS02_anthracyclineIKSb
polyketomycin_KS01_anthracyclineIKSa_polyketomycin_KS02_anthracyclineIKSb
nogalamycin_KS01_anthracyclineIKSa_nogalamycin_KS02_anthracyclineIKSb
steffimycin_KS01_anthracyclineIKSa_steffimycin_KS02_anthracyclineIKSb
kosinostatin_KS01_anthracyclineIKSa_kosinostatin_KS02_anthracyclineIKSb
erdacin_KS01_isochromanequinoneKSa_erdacin_KS02_isochromanequinoneKSb
granaticin_KS01_isochromanequinoneKSa_granaticin_KS02_isochromanequinoneKSb
naphthocyclinone_KS01_isochromanequinoneKSa_naphthocyclinone_KS02_isochromanequinoneKSb
actinorhodin_KS01_isochromanequinoneKSa_actinorhodin_KS02_isochromanequinoneKSb
enterocin_KS01_isochromanequinoneKSa_enterocin_KS02_isochromanequinoneKSb
R1128_KS01_isochromanequinoneKSa_R1128_KS02_isochromanequinoneKSb
alnumycin_KS01_isochromanequinoneKSa_alnumycin_KS02_isochromanequinoneKSb
frenolicin_KS01_isochromanequinoneKSa_frenolicin_KS02_isochromanequinoneKSb
aclacinomycin_KS01_anthracyclineIIKSa_aclacinomycin_KS02_anthracyclineIIKSb
cosmomycin_KS01_anthracyclineIIKSa_cosmomycin_KS02_anthracyclineIIKSb
doxorubicin_KS01_anthracyclineIIKSa_doxorubicin_KS02_anthracyclineIIKSb
lomaiviticin_KS01_angucyclineIIKSa_lomaiviticin_KS02_angucyclineIIKSb
nenestatin_KS01_angucyclineIIKSa_nenestatin_KS02_angucyclineIIKSb
hedamycin_KS01_aromaticKSa_hedamycin_KS02_aromaticKSb
griseorhodin_KS01_pentangularpolyphenolKSa_griseorhodin_KS02_pentangularpolyphenolKSb
rubromycin_KS01_pentangularpolyphenolKSa_rubromycin_KS02_pentangularpolyphenolKSb
lysolipin_KS01_pentangularpolyphenolKSa_lysolipin_KS02_pentangularpolyphenolKSb
xantholipin_KS01_pentangularpolyphenolKSa_xantholipin_KS02_pentangularpolyphenolKSb
fasamycin_KS01_pentangularpolyphenolKSa_fasamycin_KS02_pentangularpolyphenolKSb
arenimycin_KS01_pentangularpolyphenolKSa_arenimycin_KS02_pentangularpolyphenolKSb
pradimicin_KS01_pentangularpolyphenolKSa_pradimicin_KS02_pentangularpolyphenolKSb
A74528_KS01_pentangularpolyphenolKSa_A74528_KS02_pentangularpolyphenolKSb
fredericamycin_KS01_pentangularpolyphenolKSa_fredericamycin_KS02_pentangularpolyphenolKSb
benastatin_KS01_pentangularpolyphenolKSa_benastatin_KS02_pentangularpolyphenolKSb
FD594_KS01_pentangularpolyphenolKSa_FD594_KS02_pentangularpolyphenolKSb
elloramycin_KS01_tetracenomycinKSa_elloramycin_KS02_tetracenomycinKSb
tetracenomycin_KS01_tetracenomycinKSa_tetracenomycin_KS02_tetracenomycinKSb
lactonamycin_KS01_tetracenomycinKSa_lactonamycin_KS02_tetracenomycinKSb
gilvocarcin_KS01_aromaticKSa_gilvocarcin_KS02_aromaticKSb
StreptomycesavermitilisSP_KS01_sporepigmentKSa_StreptomycesavermitilisSP_KS02_sporepigmentKSb
StreptomycescollinusSP_KS01_sporepigmentKSa_StreptomycescollinusSP_KS02_sporepigmentKSb
StreptomycescoelicolorSP_KS01_sporepigmentKSa_StreptomycescoelicolorSP_KS02_sporepigmentKSb
resistomycin_KS01_aromaticKSa_resistomycin_KS02_aromaticKSb
0.22

**Figure S9. Maximum likelihood type II aromatic KS phylogeny of concatenated KSα and KSβ sequences from 59 biosynthetic gene clusters.** The full name of each sequence is listed on the branch tips, which can help link a query match to a specific location in the tree. Bootstrap support is listed for each node.

**Figure S10.**

## A. CDD **Condensing Enzyme Superfamily**: **634**

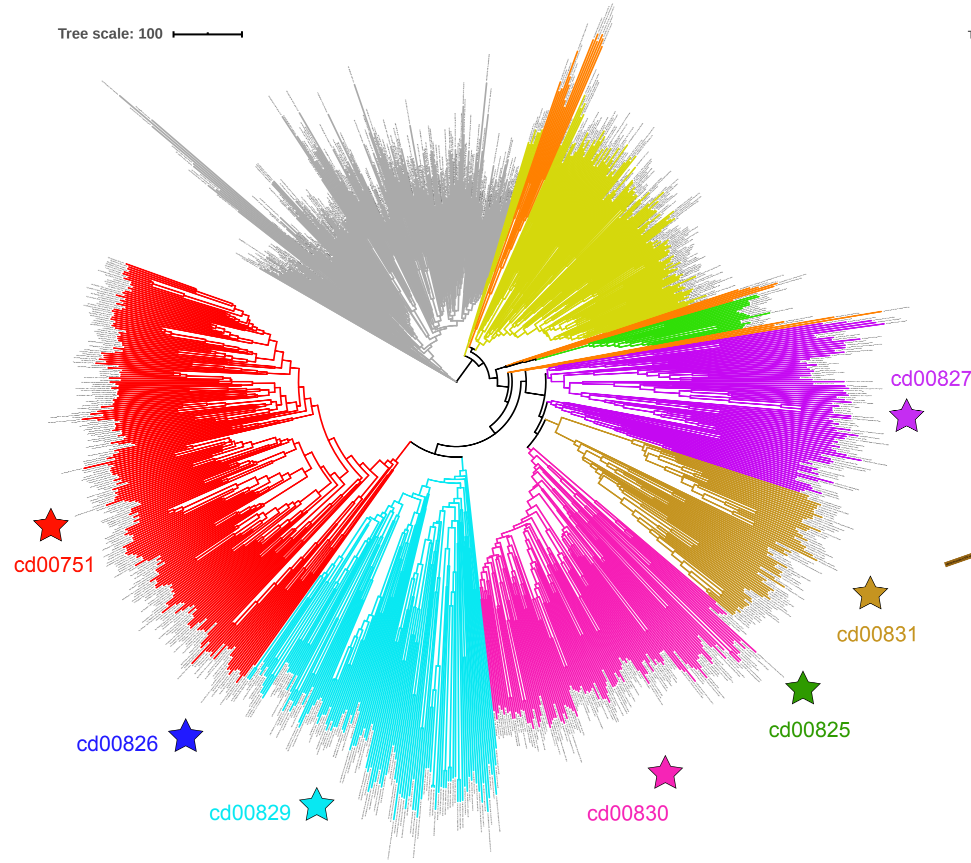


## B. **NaPDoS2** database sequences: **1877**

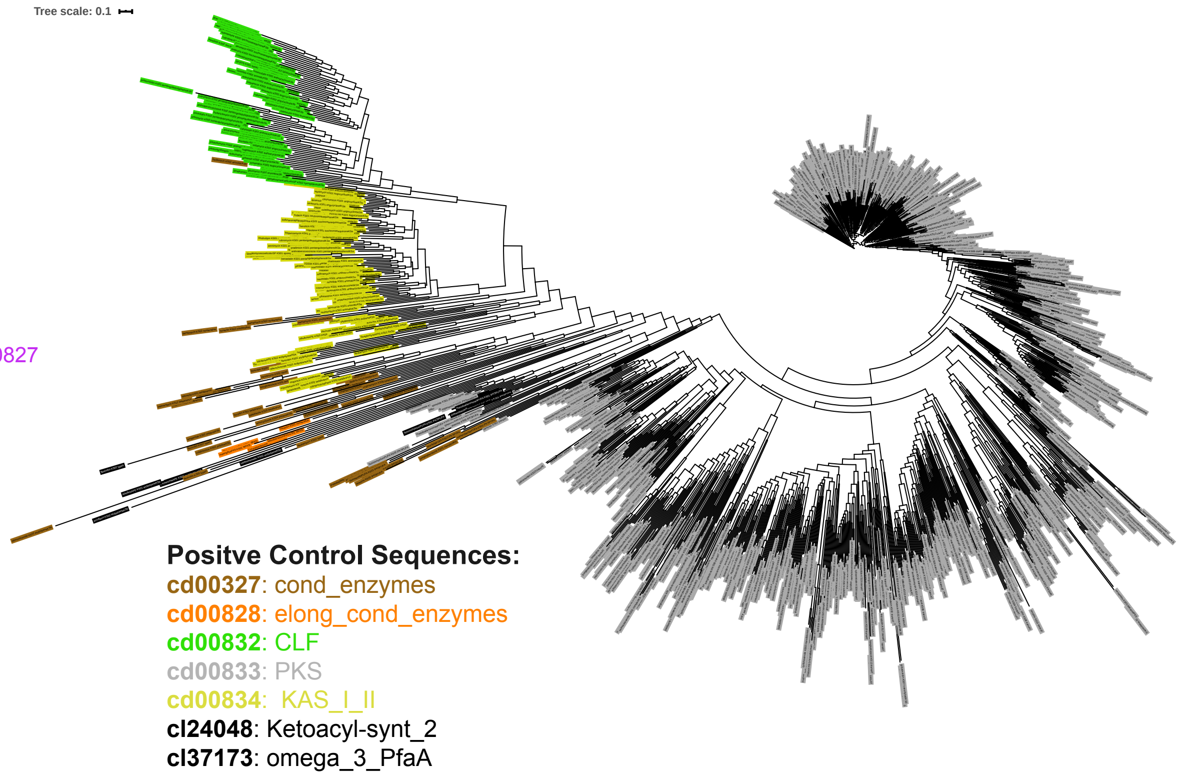


## C. **Jiang et al. 2008** tree: **49**

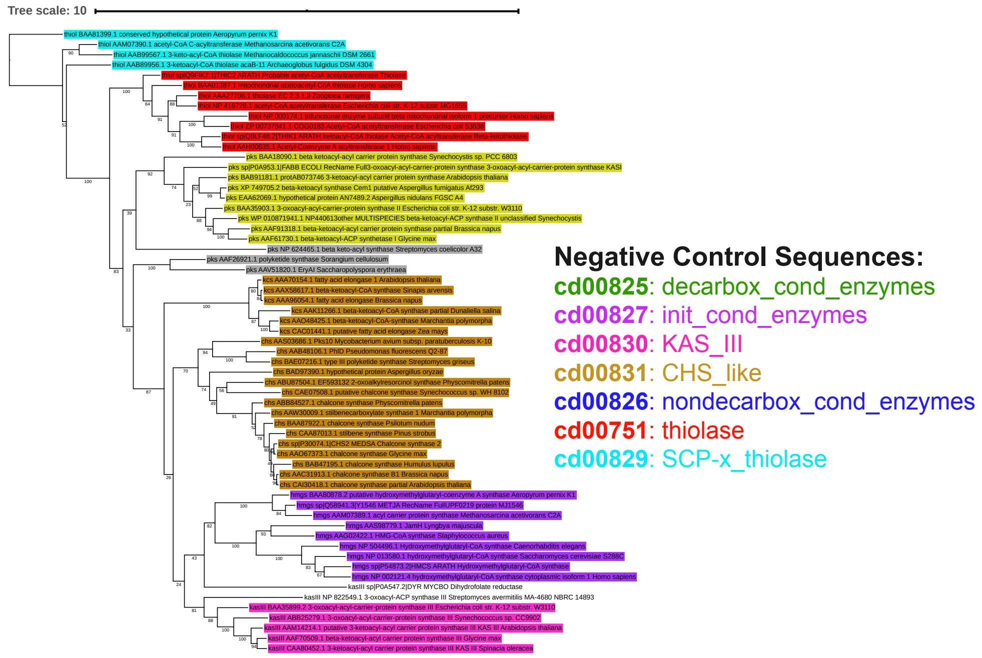


## D. **Type III PKS** MIBiG 2.0: **14**

| Accession | Main Product | Biosynthetic Class | Organism | KS Sequence |
|---|---|---|---|---|
| BGC0000189 | 3-(2'-hydroxy-3'-oxo-4'-methylpentyl)-indole<br>3-(2'-hydroxy-3'-oxo-4'-methylhexyl)-indole<br>3-(2'-acetoxy-3'-oxo-4'-methylpentyl)-indole<br>3-(2'-acetoxy-3'-oxo-4'-methylhexyl)-indole | Type III Polyketide | *Xenorhabdus bovienii* SS-2004 | XBJ1_3898<br>XBJ1_3900 |
| BGC0000285 | flaviolin rhamnoside<br>3,3'-diflaviolin<br>flaviolin | Type III Polyketide<br>Oligosaccharide | *Saccharopolyspora erythraea* | rppA |
| BGC0001064 | cylindrocyclophane D<br>cylindrocyclophane E<br>cylindrocyclophane F | Modular Type I Polyketide<br>Type III Polyketide | *Cylindrospermum licheniforme* UTEX B 2014 | AFV96140<br>AFV96143 |
| BGC0001079 | napyradiomycin A80915C | Terpene<br>Type III Polyketide | *Streptomyces aculeolatus* | napT1<br>napB1 |
| BGC0001083 | merochlorin A, merochlorin B<br>deschloro-merochlorin A, deschloro-merochlorin B<br>isochloro-merochlorin B, dichloro-merochlorin B<br>merochlorin D, merochlorin C | Terpene<br>Type III Polyketide | *Streptomyces sp.* CNH189 | mcl3<br>mcl4<br>mcl17 |
| BGC0001150 | pamamycin-607 | Type II Polyketide<br>Type III Polyketide | *Streptomyces alboniger* | pamG<br>pamD<br>pamE<br>pamK |

**Figure S10**. **Negative control KS sequence selection.** All sequence accession information is listed in Table S3.

A) Distance matrix tree of 1,072 condensing enzyme superfamily (cl09938) sequences from the NCBI Conserved Domain Database[6] (CDD) tool CDtree[7], colored by conserved domain (CD) family in iTOL[8] (see panel B and C keys). Stars mark CD families not represented in the NaPDoS2 database, which were selected as negative controls (634 total). The negative control CD families comprise: initiating condensing enzymes (init_cond_enzymes, cd00827; n=84), chalcone and stilbene synthases (CHS_like, cd00831; n=67), decarboxylating condensing enzymes (decarbox_cond_enzymes, cd00825; n=3), ketoacyl-acyl carrier protein synthase III enzymes (KAS_III, cd00830; n=130), sterol carrier protein (SCP)-x isoform-associated thiolase domains (SCP-x_thiolase, cd00829; n=125), non-decarboxylating condensing enzymes (nondecarbox_cond_enzymes, cd00826; n=2), and thiolase enzymes (thiolase, cd00751; n=223).

B) Phylogenetic tree of the 1,877 NaPDoS2 KS sequences colored by CD family as determined by NCBI CD-Search[6] (see key). These sequences, which are associated with experimentally verified PKS and FAS biosynthetic gene clusters (BGCs), were selected as positive controls. The 1,877 NaPDoS2 KS sequences were aligned using MUSCLE[9]; the phylogenetic tree was calculated using FastTreeMP[10] on the CIPRES Science Gateway[11] and visualized in iTOL[8]. The NaPDoS2 database positive control CD families comprise: the condensing enzymes subfamily (cond_enzymes, cd00327; n=39), the elongating condensing enzyme subfamily (elong_cond_enzymes, cd00828; n=5), the chain-length factor subfamily (CLF, cd00832; n=70), the polyketide synthase PKS subfamily (PKS, cd00833; 1,649), the beta-ketoacyl-acyl carrier protein synthase (KAS) type I and II subfamily (KAS_I_II, cd00834; n=102), the N-terminal domain beta-ketoacyl synthase pfam13723 superfamily (cl24048; n=5), and the polyketide-type polyunsaturated fatty acid synthase omega-3 PfaA TIGR02813 superfamily (cl37173; n=7).

C) Phylogenetic tree of 61 condensing enzyme superfamily sequences from Jiang *et al* 2008[12], colored by conserved domain family as determined by NCBI CD-Search[6] (see panel B and C keys). Of these, 49 sequences (all but the 12 sequences colored yellow and grey) were added to the pool of negative control sequences. The 61 sequences were aligned using MUSCLE[9]; ProtTest 3.4.2[13] was used to define a model; the phylogeny was calculated with RAxML[14] WAG+G with 200 bootstraps; and the resulting tree visualized in iTOL[8].

D) Fourteen KS domains from the six experimentally characterized type III PKS BGCs in the MIBiG 2.0[5] repository were also added to the pool of negative control sequences. Sequence names are colored by their CD family as determined by NCBI CD-Search[6].

Figure S11.

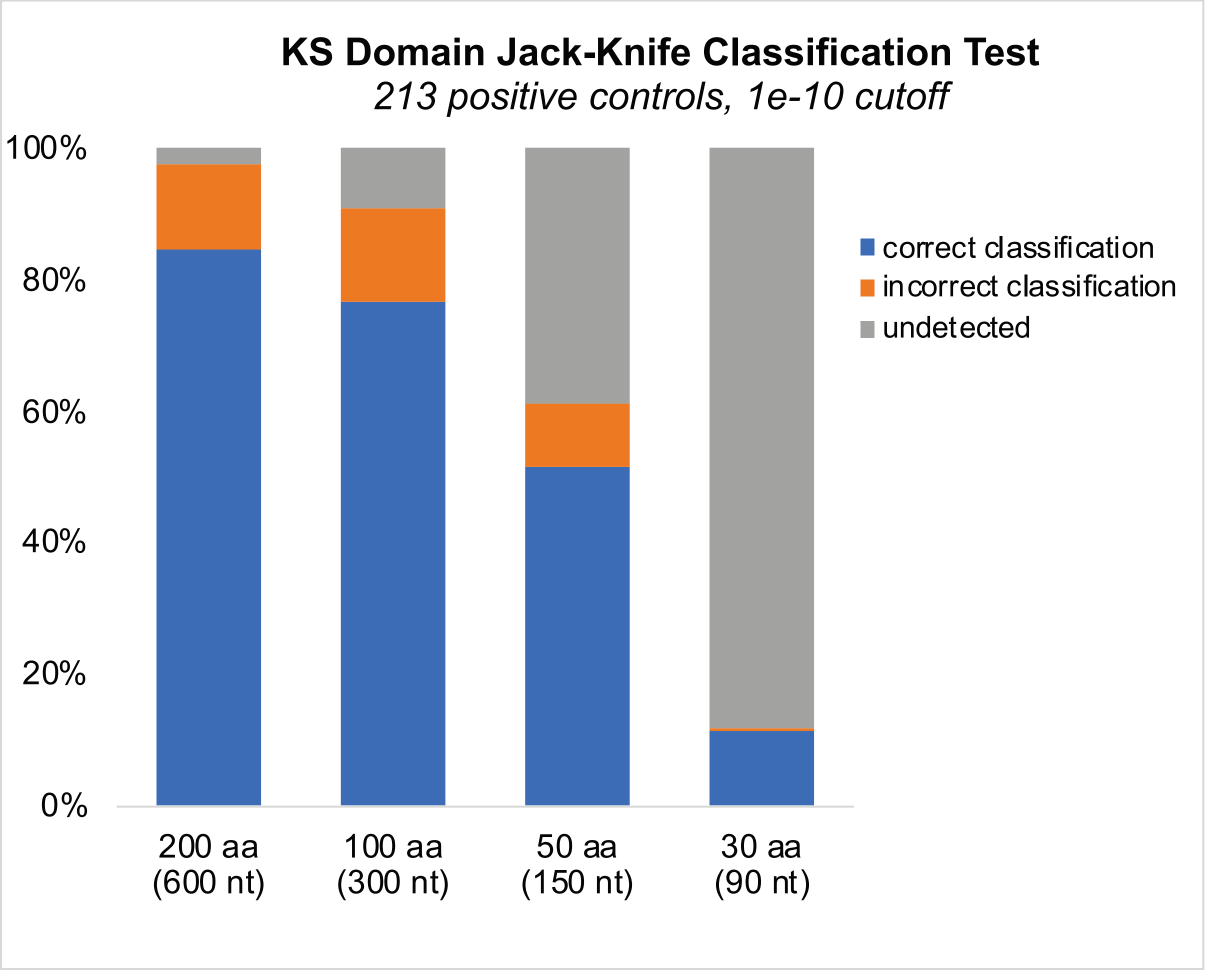
KS Domain Jack-Knife Classification Test
213 positive controls, 1e-10 cutoff
100%
80%
60%
40%
20%
0%
200 aa
(600 nt)
100 aa
(300 nt)
50 aa
(150 nt)
30 aa
(90 nt)
correct classification
incorrect classification
undetected

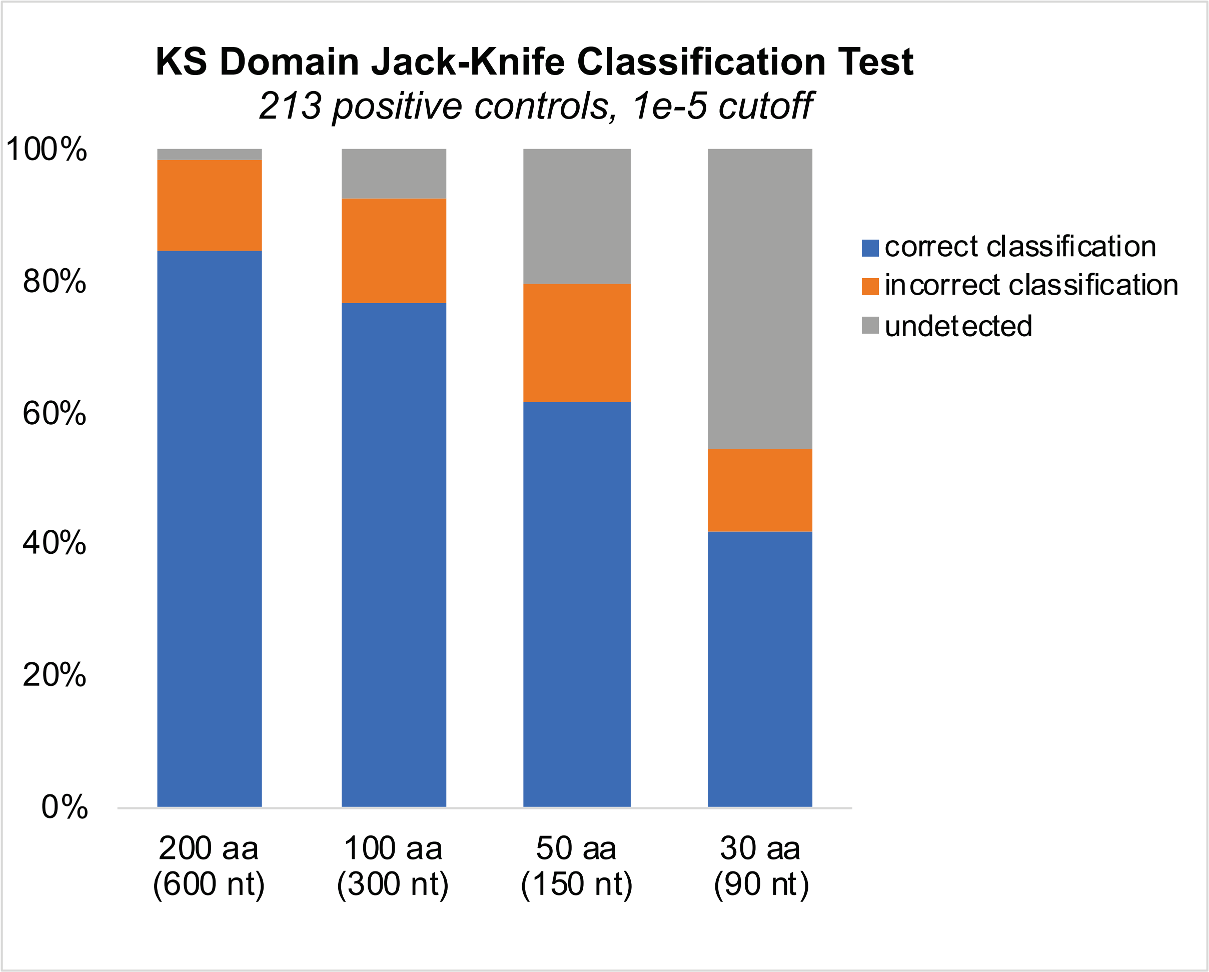
KS Domain Jack-Knife Classification Test
213 positive controls, 1e-5 cutoff
100%
80%
60%
40%
20%
0%
200 aa
(600 nt)
100 aa
(300 nt)
50 aa
(150 nt)
30 aa
(90 nt)
correct classification
incorrect classification
undetected

**Figure S11**. **Effect of query size on KS detection and classification accuracy at various E-values.** Classifications were based on varying BLASTP e-value cutoff scores as indicated for the closest non-self database match. Test sequences of varying lengths were obtained as overlapping sliding window subsequences covering the full length of 213 non-redundant, positive control KS domains.

**Figure S12.**

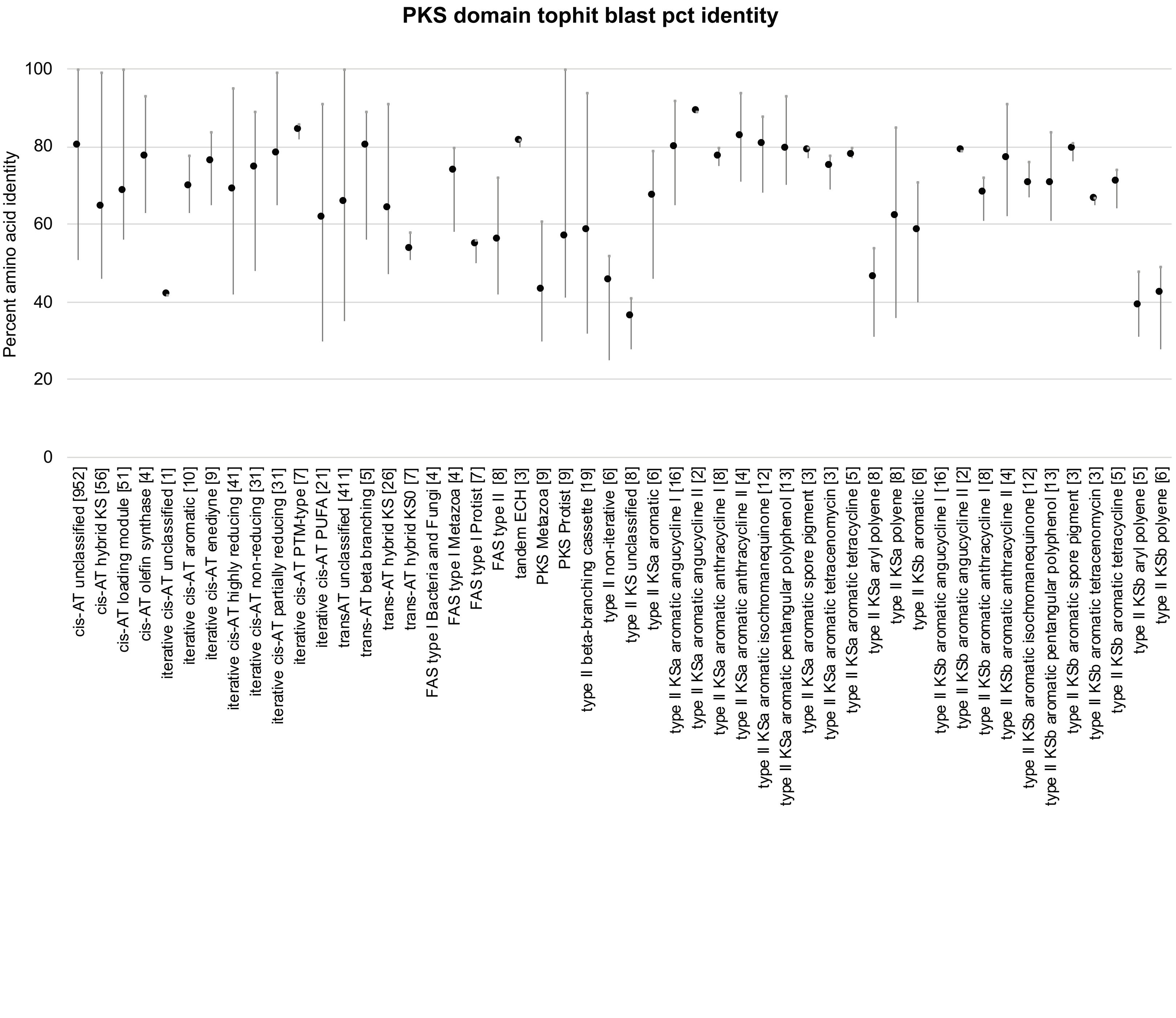
PKS domain tophit blast pct identity
Percent amino acid identity
100
80
60
40
20
0
cis-AT unclassified [952]
cis-AT hybrid KS [56]
cis-AT loading module [51]
cis-AT olefin synthase [4]
iterative cis-AT unclassified [1]
iterative cis-AT aromatic [10]
iterative cis-AT enediyne [9]
iterative cis-AT highly reducing [41]
iterative cis-AT non-reducing [31]
iterative cis-AT partially reducing [31]
iterative cis-AT PTM-type [7]
iterative cis-AT PUFA [2]
transAT unclassified [41]
trans-AT beta branching [5]
trans-AT hybrid KS [26]
trans-AT hybrid KS0 [7]
FAS type I Bacteria and Fungi [4]
FAS type I Metazoa [4]
FAS type I Protist [7]
FAS type II [8]
tandem ECH [3]
PKS Metazoa [9]
PKS Protist [9]
type II beta-branching cassette [19]
type II non-iterative [6]
type II KS unclassified [8]
type II KSa aromatic [6]
type II KSa aromatic angucycline I [16]
type II KSa aromatic angucycline II [2]
type II KSa aromatic anthracycline I [8]
type II KSa aromatic anthracycline II [4]
type II KSa aromatic isochromanequinone [12]
type II KSa aromatic pentangular polyphenol [13]
type II KSa aromatic spore pigment [3]
type II KSa aromatic tetracenomycin [3]
type II KSa aromatic tetracycline [5]
type II KSa aryl polyene [8]
type II KSa polyene [8]
type II KSb aromatic [6]
type II KSb aromatic angucycline I [16]
type II KSb aromatic angucycline II [2]
type II KSb aromatic anthracycline I [8]
type II KSb aromatic anthracycline II [4]
type II KSb aromatic isochromanequinone [12]
type II KSb aromatic pentangular polyphenol [13]
type II KSb aromatic spore pigment [3]
type II KSb aromatic tetracenomycin [3]
type II KSb aromatic tetracycline [5]
type II KSb aryl polyene [5]
type II KSb polyene [6]

**Figure S12. KS domain sequence diversity.** Percent sequence identities were determined for each KS class in the NaPDoS2 database using an all-against-all BLASTP comparison. Mean values (points) and ranges (vertical lines) are shown for top non-self matches within each NaPDoS2 class (x-axis). Bracketed values indicate the total number of database sequences in that class.

**Figure S13.**

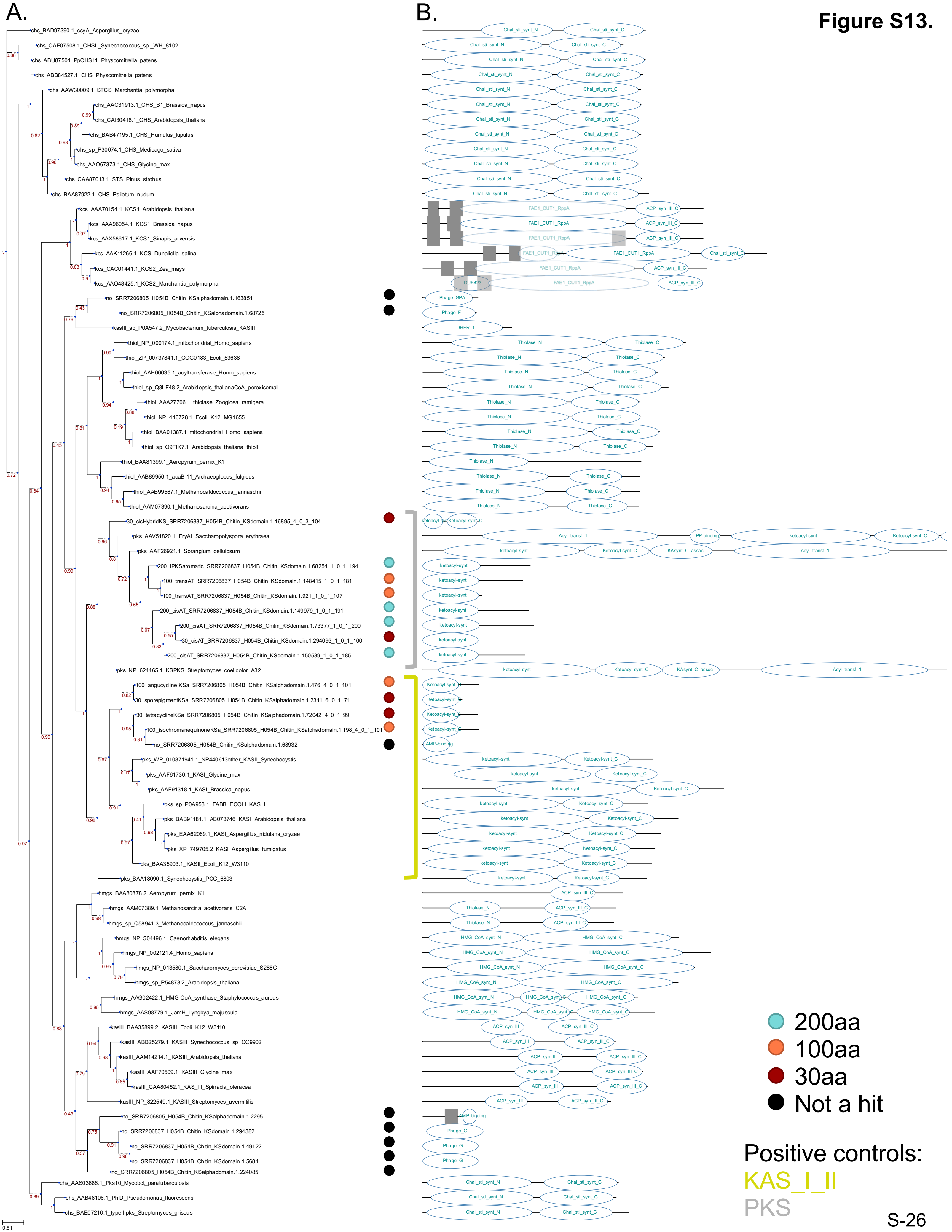
A.
B.
chs_BAD97390.1_csyA_Aspergillus_oryzae
chs_CAE07508.1_CHSL_Synechococcus_sp._WH_8102
chs_ABU87504_PpCHS11_Physcomitrella_patens
chs_ABB84527.1_CHS_Physcomitrella_patens
chs_AAW30009.1_STCS_Marchantia_polymorpha
chs_AAC31913.1_CHS_B1_Brassica_napus
chs_CAI30418.1_CHS_Arabidopsis_thaliana
chs_BAB47195.1_CHS_Humulus_lupulus
chs_sp_P30074.1_CHS_Medicago_sativa
chs_AAO67373.1_CHS_Glycine_max
chs_CAA87013.1_STS_Pinus_strobus
chs_BAA87922.1_CHS_Psilotum_nudum
kcs_AAA70154.1_KCS1_Arabidopsis_thaliana
kcs_AAA96054.1_KCS1_Brassica_napus
kcs_AAX58617.1_KCS1_Sinapis_arvensis
kcs_AAK11266.1_KCS_Dunaliella_salina
kcs_CAC01441.1_KCS2_Zea_mays
kcs_AAO48425.1_KCS2_Marchantia_polymorpha
no_SRR7206805_H054B_Chitin_KSalphadomain.1.163851
no_SRR7206805_H054B_Chitin_KSalphadomain.1.68725
kasIII_sp_P0A547.2_Mycobacterium_tuberculosis_KASIII
thiol_NP_000174.1_mitochondrial_Homo_sapiens
thiol_ZP_00737841.1_COG0183_Ecoli_53638
thiol_AAH00635.1_acyltransferase_Homo_sapiens
thiol_sp_Q8LF48.2_Arabidopsis_thalianaCoA_peroxisomal
thiol_AAA27706.1_thiolase_Zoogloea_ramigera
thiol_NP_416728.1_Ecoli_K12_MG1655
thiol_BAA01387.1_mitochondrial_Homo_sapiens
thiol_sp_Q9FIK7.1_Arabidopsis_thaliana_thiolI
thiol_BAA81399.1_Aeropyrum_pernix_K1
thiol_AAB89956.1_acaB-11_Archaeoglobus_fulgidus
thiol_AAB99567.1_Methanocaldococcus_jannaschii
thiol_AAM07390.1_Methanosarcina_acetivorans
30_cisHybridKS_SRR7206837_H054B_Chitin_KSdomain.1.16895_4_0_3_104
pks_AAV51820.1_EryAI_Saccharopolyspora_erythraea
pks_AAF26921.1_Sorangium_cellulosum
200_iPKSaromatic_SRR7206837_H054B_Chitin_KSdomain.1.68254_1_0_1_194
100_transAT_SRR7206837_H054B_Chitin_KSdomain.1.148415_1_0_1_181
100_transAT_SRR7206837_H054B_Chitin_KSdomain.1.921_1_0_1_107
200_cisAT_SRR7206837_H054B_Chitin_KSdomain.1.149979_1_0_1_191
200_cisAT_SRR7206837_H054B_Chitin_KSdomain.1.73377_1_0_1_200
30_cisAT_SRR7206837_H054B_Chitin_KSdomain.1.294093_1_0_1_100
200_cisAT_SRR7206837_H054B_Chitin_KSdomain.1.150539_1_0_1_185
pks_NP_624465.1_KSPKS_Streptomyces_coelicolor_A32
100_angucyclineKSa_SRR7206805_H054B_Chitin_KSalphadomain.1.476_4_0_1_101
30_sporepigmentKSa_SRR7206805_H054B_Chitin_KSalphadomain.1.2311_6_0_1_71
30_tetracyclineKSa_SRR7206805_H054B_Chitin_KSalphadomain.1.72042_4_0_1_99
100_isochromanequinoneKSa_SRR7206805_H054B_Chitin_KSalphadomain.1.198_4_0_1_101
no_SRR7206805_H054B_Chitin_KSalphadomain.1.68932
pks_WP_010871941.1_NP440613other_KASII_Synechocystis
pks_AAF61730.1_KASI_Glycine_max
pks_AAF91318.1_KASI_Brassica_napus
pks_sp_P0A953.1_FABB_ECOLI_KAS_I
pks_BAB91181.1_AB073746_KASI_Arabidopsis_thaliana
pks_EAA62069.1_KASI_Aspergillus_nidulans_oryzae
pks_XP_749705.2_KASI_Aspergillus_fumigatus
pks_BAA35903.1_KASII_Ecoli_K12_W3110
pks_BAA18090.1_Synechocystis_PCC_6803
hmgs_BAA80878.2_Aeropyrum_pernix_K1
hmgs_AAM07389.1_Methanosarcina_acetivorans_C2A
hmgs_sp_Q58941.3_Methanocaldococcus_jannaschii
hmgs_NP_504496.1_Caenorhabditis_elegans
hmgs_NP_002121.4_Homo_sapiens
hmgs_NP_013580.1_Saccharomyces_cerevisiae_S288C
hmgs_sp_P54873.2_Arabidopsis_thaliana
hmgs_AAG02422.1_HMG-CoA_synthase_Staphylococcus_aureus
hmgs_AAS98779.1_JamH_Lyngbya_majuscula
kasIII_BAA35899.2_KASIII_Ecoli_K12_W3110
kasIII_ABB25279.1_KASIII_Synechococcus_sp_CC9902
kasIII_AAM14214.1_KASIII_Arabidopsis_thaliana
kasIII_AAF70509.1_KASIII_Glycine_max
kasIII_CAA80452.1_KAS_III_Spinacia_oleracea
kasIII_NP_822549.1_KASIII_Streptomyces_avermitilis
no_SRR7206805_H054B_Chitin_KSalphadomain.1.2295
no_SRR7206837_H054B_Chitin_KSdomain.1.294382
no_SRR7206837_H054B_Chitin_KSdomain.1.49122
no_SRR7206837_H054B_Chitin_KSdomain.1.5684
no_SRR7206805_H054B_Chitin_KSalphadomain.1.224085
chs_AAS03686.1_Pks10_Mycobct_paratuberculosis
chs_AAB48106.1_PhlD_Pseudomonas_fluorescens
chs_BAE07216.1_typeIIIpks_Streptomyces_griseus
0.81
200aa
100aa
30aa
Not a hit
Positive controls:
KAS_I_II
PKS

**Figure S13**. **Amplicon detection accuracy.** A) Phylogenetic tree of 20 amplicons from Elfeki *et al.* 2018[15] (bolded) and 61 condensing enzyme superfamily sequences from Jiang *et al.* 2008[12]. The 20 amplicons from Elfeki *et al.* 2018[15] (bolded) were detected by NaPDoS2 at 30aa, 100aa, or 200aa minimum alignment lengths (red, orange, or teal circle, respectively) or not detected (not a "hit") at any alignment length (black circle). Grey and yellow brackets include positive control KS sequences as described in Figure S10. B) The conserved domains of all sequences as defined by TREND[16] (http://trend.zhulinlab.org/) from the NCBI conserved domain database are illustrated (gray squares are predicted transmembrane regions). The example "KS" amplicons not detected by NaPDoS2 are off-target amplifications with conserved domains relating to phage proteins. The KS amplicons detected by NaPDoS2 cluster within the PKS and KAS_I_II conserved domain positive control sequences from type I and II PKSs, respectively.

Abbreviations in (A) taxa branches from Jiang *et al.* 2008[12] are as listed in Figure S10; sequences from Elfeki *et al.* 2018[15] are annotated with the minimum amino acid alignment length setting/or "no" for "not a hit", NaPDoS2 classification, SRA dataset, sample information, and NaPDoS2 domain range.
Abbreviations in (B) conserved domains as defined by TREND[16].

**Table S1.**

**Table S1. Typical NaPDoS pipeline processing times for genomic, PCR amplicon, and metagenomic data sets for the original NaPDoS release (V1) and NaPDoS2 (V2).** Initial data upload times are not included, as they vary widely according to internet connection speed. Matches were analyzed using a minimum alignment length of 200 amino acids for all data sets except the 243 nt amplicons, which were screened using a 50 amino acid cutoff value. Dashes indicate missing values for queries that could not be analyzed in NaPDoS version 1 because they exceeded maximum size limitations (50,000 sequences or 30 MB file size). Limits in NaPDoS Version 2 have been increased to 500,000 sequences or 500 MB file size.

| Data set | Type | Acession numbers | File size (MB) | Input seqs | V1 sec | V2 sec | Num. domain matches |
|---|---|---|---|---|---|---|---|
| *Salinispora arenicola* CNS-205, complete genome | nucleic acid | GCF_000018265.1 | 5.9 | 1 | 106 | 10 | 33 |
| Aplysina aerophoba assembed metagenome - random subsample | nucleic acid (assembled contigs) | IMG 3300002222 | 32.9 | 1,250 | 192 | 13 | 32 |
| Aplysina aerophoba assembed metagenome - random subsample | nucleic acid (assembled contigs) | IMG 3300002222 | 46.1 | 2,500 | 276 | 19 | 38 |
| Aplysina aerophoba assembed metagenome - random subsample | nucleic acid (assembled contigs) | IMG 3300002222 | 82.5 | 10,000 | - | 33 | 62 |
| Aplysina aerophoba assembed metagenome - random subsample | nucleic acid (assembled contigs) | IMG 3300002222 | 106.4 | 20,000 | - | 42 | 72 |
| Aplysina aerophoba assembed metagenome - random subsample | nucleic acid (assembled contigs) | IMG 3300002222 | 141 | 49,000 | - | 57 | 87 |
| Aplysina aerophoba assembed metagenome | nucleic acid (assembled contigs) | IMG 3300002222 | 211.4 | 219,427 | - | 91 | 94 |
| | | | | | | | |
| *Salinispora arenicola* CNS-205, complete genome | predicted protein | GCF_000018265.1 | 2.0 | 4,820 | 22 | 6 | 33 |
| 23 draft bacterial genomes (MAGs) - random subsample | predicted protein | PRJNA320446 | 9.5 | 25,000 | 79 | 4 | 16 |
| 23 draft bacterial genomes (MAGs) - random subsample | predicted protein | PRJNA320446 | 13.4 | 35,000 | 107 | 6 | 20 |
| 23 draft bacterial genomes (MAGs) - random subsample | predicted protein | PRJNA320446 | 19.1 | 50,000 | 149 | 7 | 31 |
| 23 draft bacterial genomes (MAGs) | predicted protein | PRJNA320446 | 28.1 | 73,530 | 216 | 9 | 49 |
| Aplysina aerophoba assembed metagenome | predicted protein | IMG 3300002222 | 70.9 | 365,131 | - | 41 | 94 |
| | | | | | | | |
| S31 Antarctic soil, KS amplicons - random subsample | nucleic acid amplicon (243 nt) | ERR1527879 | 1.5 | 5,000 | 50 | 8 | 3,194 |
| S31 Antarctic soil, KS amplicons | nucleic acid amplicon (243 nt) | ERR1527879 | 6.5 | 19,909 | 198 | 36 | 14,024 |

**Table S2. NaPDoS2 database summary.** Table lists the number of KS domains and their phylum level assignments for each class and subclass.

# Table S2.

| Class | Subclass | Bacteria | Fungi | Other Eukaryota | Total (across all taxa) |
|---|---|---|---|---|---|
| **Polyketide Synthases** | | | | | |
| type I modular *cis*-AT | olefin synthase | 4 | | | 4 |
| | loading module | 51 | | | 51 |
| | hybrid KS | 56 | | | 56 |
| | tandem ECH | 3 | | | 3 |
| | no subclass | 952 | | | 952 |
| | **Total** | **1066** | **0** | **0** | **1066** |
| type I iterative *cis*-AT | PUFA (polyunsaturated fatty acids) | 18 | | 3 | 21 |
| | enediynes | 9 | | | 9 |
| | aromatic | 10 | | | 10 |
| | PTM-type (polycyclic tetramate macrolactam) | 7 | | | 7 |
| | non-reducing | | 31 | | 31 |
| | partially reducing | | 7 | | 7 |
| | highly reducing | | 41 | | 41 |
| | no subclass | 1 | | | 1 |
| | **Total** | **45** | **79** | **3** | **127** |
| type I *trans*-AT | beta-branching module | 5 | | | 5 |
| | hybrid KS | 19 | | | 19 |
| | hybrid KS0 (non-elongating KS) | 7 | | | 7 |
| | no subclass | 411 | | | 411 |
| | **Total** | **442** | **0** | **0** | **442** |
| type I Metazoa-type PKS | no subclass | | | 9 | 9 |
| type I Protist-type PKS | no subclass | | | 9 | 9 |
| | **Total** | **0** | **0** | **18** | **18** |
| | **Total** | **1553** | **79** | **21** | **1653** |
| type II aromatic | angucycline-derived I | 32 | | | 32 |
| | angucycline-derived II | 4 | | | 4 |
| | anthracycline-derived I | 16 | | | 16 |
| | anthracycline-derived II | 8 | | | 8 |
| | isochromanequinone-derived | 24 | | | 24 |
| | pentangular polyphenol-derived | 26 | | | 26 |
| | tetracenomycin-derived | 6 | | | 6 |
| | tetracycline-derived | 10 | | | 10 |
| | spore pigment | 6 | | | 6 |
| | unclassified | 12 | | | 12 |
| | **Total** | **144** | **0** | **0** | **144** |
| type II beta-branching cassettes | no subclass | 19 | | | 19 |
| type II polyenes | KSa, KSb | 14 | | | 14 |
| type II aryl polyenes | KSa, KSb | 13 | | | 13 |
| type II non-iterative | no subclass | 6 | | | 6 |
| type II unclassified | no subclass | 5 | | | 5 |
| | **Total** | **57** | **0** | **0** | **57** |
| | **Total** | **201** | **0** | **0** | **201** |
| **Fatty acid synthases** | | | | | |
| type I FAS | Bacterial-Fungal-type | 2 | 2 | | 4 |
| | Metazoan-type | | | 4 | 4 |
| | Protist-type | | | 7 | 7 |
| | **Total** | **2** | **2** | **11** | **15** |
| type II FAS | no subclass | 8 | | | 8 |
| | **Total** | **8** | **0** | **0** | **8** |
| | **Total** | **10** | **2** | **11** | **23** |
| | **Grand Total** | **1764** | **81** | **32** | **1877** |

**Table S3. Accession numbers for negative controls, application use cases, type II aromatic KS sequences, example structures, and associated references.**

The first tab "Negative Control Sequences" lists the accession numbers for the 697 negative control sequences listed by source: NCBI Conserved Domain family outside, Jiang *et al.* 2008[12] tree, and MIBiG 2.0[5] type III PKS BGCs.

The second tab "Application Use Case Accessions" lists the sequence/dataset accession numbers for the application use cases (listed by biological source type, data type, and relevant reference).

The third tab "TypeIIAromaticKS_ AccessNum_Refs" lists the GenBank protein ID for alpha and beta sequences, together with literature references used for the biosynthetic annotations in Figure 4 (i.e. poly-beta-keto chain length, starting unit, C-C cyclisation position).

The fourth tab "Example Structures Metadata" lists the PubMed ID/doi for the structures shown in Figures 2 and 5 along with the KS class/subclass associated with its biosynthesis, the chemical name, the BGC product name as reported in the NaPDoS2 BGCs tab, and the source species.

(provided as a Microsoft Excel file).

**Table S4**. ***Salinispora* spp. type II KS domains identified by NaPDoS version 1 (NaPDoS1)[1] and NaPDoS2.** 118 *Salinispora* genomes[17] were analyzed using the following default settings: NaPDoS1: HMM 1e-5 cutoff, 200aa minimum alignment length, pathway assignment BLASTP e-value 1e-5 cutoff; NaPDoS2: e-value cutoff 1e-8 and 200aa minimum alignment length. The NaPDoS1 output is limited to the total number of type II KSs detected. The NaPDoS2 output includes the total number of type II KSs ("Total NP2") and their subclassification. KSα and KSβ sequences are grouped together.

Abbreviations: Betabranch= type II beta-branching cassettes; Angl-I= type II aromatic angucycline-derived I; Angl-II= type II aromatic angucycline-derived II; Anth-I= type II aromatic anthracycline-derived I; Isochrom= type II aromatic isochromanequinone-derived; Polyphen= type II aromatic pentangular polyphenol-derived; Unclass= type II aromatic unclassified; Type II-uc= type II unclassified no subclass.

**Table S4.**

| | | NaPDoS1 | NaPDoS2 | | | | | | | | | |
|---|---|---|---|---|---|---|---|---|---|---|---|---|
| | | | | | | Aromatic | | | | | | |
| **Species** | **# genomes** | Type II | *Total NP2* | Betabranch | Polyene | Angl-I | Angl-II | Anth-I | Isochrom | Polyphen | Unclass | Type II-uc |
| *S. tropica* | 12 | 48 | 108 | - | 60 | - | 24 | - | - | 24 | - | - |
| *S. fenicalii* | 2 | 12 | 24 | - | 12 | 4 | 4 | - | - | 4 | - | - |
| *S. cortesiana* | 1 | 4 | 5 | - | 1 | - | 2 | - | - | 2 | - | - |
| *S. mooreana* | 3 | 8 | 15 | - | 7 | - | - | - | 2 | 6 | - | - |
| *S. oceanensis* | 12 | 44 | 44 | - | - | 8 | - | 6 | 4 | 26 | - | - |
| *S. goodfellowii* | 1 | 4 | 5 | - | 1 | - | 2 | - | - | 2 | - | - |
| *S. vitiensis* | 3 | 12 | 28 | - | 14 | - | 6 | - | - | 6 | - | 2 |
| *S. pacifica* | 23 | 97 | 126 | - | 27 | - | 46 | - | 4 | 46 | - | 3 |
| *S. arenicola* | 61 | 134 | 307 | 2 | 173 | - | - | 4 | - | 126 | 2 | - |

**Table S5. Complete list of *Salinispora* spp. KS domains identified by NaPDoS2.** 118 *Salinispora* genomes[17] were analyzed using default settings (e-value cutoff 1e-8 and 200aa minimum alignment length). Table lists the number of KSs detected and their classification by strain. The summed total number of KSs found in each strain is listed as "NP2 Total". KSα and KSβ sequences are grouped together.

Abbreviations: Betabranch= type II beta-branching cassettes; Angl-I= type II aromatic angucycline-derived I; Angl-II= type II aromatic angucycline-derived II; Anth-I= type II aromatic anthracycline-derived I; Isochrom= type II aromatic isochromanequinone-derived; Polyphen= type II aromatic pentangular polyphenol-derived; Unclass= type II aromatic unclassified; Type II-uc= type II unclassified no subclass; *cis*-AT= type I modular *cis*-AT; cisloading= type I modular *cis*-AT loading module; cisHybridKS= type I modular *cis*-AT hybrid KS; Ened= type I iterative *cis*-AT enediynes; iPKSaromatic= type I iterative *cis*-AT aromatic; PTM= type I iterative *cis*-AT PTM-type (polycyclic tetramate macrolactam); *trans*-AT= type I *trans*-AT no subclass; *trans*-hybridKS0= type I *trans*-AT hybrid KS0 (non-elongating KS).

# Table S5.

NaPDoS2: KS classes

| | | | FAS | Type II PKS | | | | | | | | | Type I PKS | | | | | | | |
|---|---|---|---|---|---|---|---|---|---|---|---|---|---|---|---|---|---|---|---|---|
| | | *NP2* | | | | Aromatic | | | | | | | | *cis*-AT modular | | *cis*-AT iterative | | | *trans*-AT | |
| *Species* | *Strain* | *Total* | FAS II | Betabranch | Polyene | Angl-I | Angl-II | Anth-I | Isochrom | Polyphen | Unclass | TypeII-uc | *cis*-AT | cisloading | cisHybridKS | Ened | iPKSaromatic | PTM | *trans*-AT | *trans*-hybridKS0 |
| *S. tropica* | CNB440 | 28 | 2 | | 5 | | 2 | | | 2 | | | 12 | 1 | 2 | 2 | | | | |
| *S. tropica* | CNB476 | 22 | 2 | | 5 | | 2 | | | 2 | | | 6 | 1 | 3 | 1 | | | | |
| *S. tropica* | CNB536 | 29 | 3 | | 5 | | 2 | | | 2 | | | 12 | 1 | 3 | 1 | | | | |
| *S. tropica* | CNH898 | 27 | 4 | | 5 | | 2 | | | 2 | | | 10 | 1 | 2 | 1 | | | | |
| *S. tropica* | CNR699 | 24 | 2 | | 5 | | 2 | | | 2 | | | 8 | 1 | 2 | 2 | | | | |
| *S. tropica* | CNS197 | 24 | 2 | | 5 | | 2 | | | 2 | | | 8 | 1 | 2 | 2 | | | | |
| *S. tropica* | CNS416 | 24 | 2 | | 5 | | 2 | | | 2 | | | 9 | 1 | 2 | 1 | | | | |
| *S. tropica* | CNT250 | 24 | 2 | | 5 | | 2 | | | 2 | | | 8 | 1 | 2 | 2 | | | | |
| *S. tropica* | CNT261 | 27 | 3 | | 5 | | 2 | | | 2 | | | 8 | 1 | 5 | 1 | | | | |
| *S. tropica* | CNY012 | 24 | 3 | | 5 | | 2 | | | 2 | | | 8 | 1 | 2 | 1 | | | | |
| *S. tropica* | CNY678 | 24 | 2 | | 5 | | 2 | | | 2 | | | 8 | 1 | 2 | 2 | | | | |
| *S. tropica* | CNY681 | 24 | 2 | | 5 | | 2 | | | 2 | | | 8 | 1 | 2 | 2 | | | | |
| *S. fenicalii* | CNT569 | 30 | 2 | | 6 | 2 | 2 | | | 2 | | | 2 | 1 | 2 | 2 | | | 9 | |
| *S. fenicalii* | CNR942 | 29 | 2 | | 6 | 2 | 2 | | | 2 | | | 2 | 1 | 2 | 1 | | | 9 | |
| *S. cortesiana* | CNY202 | 13 | 4 | | 1 | | 2 | | | 2 | | | 2 | | 1 | 1 | | | | |
| *S. mooreana* | CNY646 | 24 | 5 | | 5 | | | | | 2 | | | 7 | 2 | 2 | 1 | | | | |
| *S. mooreana* | CNS237 | 48 | 4 | | 1 | | | | | 2 | | | 34 | 3 | 2 | 2 | | | | |
| *S. mooreana* | CNT150_DSM45549 | 13 | 4 | | 1 | | | | 2 | 2 | | | 2 | | | 2 | | | | |
| *S. oceanensis* | CNT854 | 10 | 4 | | | | | | | 2 | | | 1 | | 1 | 1 | 1 | | | |
| *S. oceanensis* | CNT584 | 13 | 4 | | | 4 | | | | 2 | | | 1 | | 1 | 1 | | | | |
| *S. oceanensis* | CNT124 | 13 | 4 | | | 4 | | | | 2 | | | 1 | | 1 | 1 | | | | |
| *S. oceanensis* | CNT138_DSM45547 | 30 | 4 | | | | | | | 4 | | | 17 | 1 | 2 | 1 | | | 1 | |
| *S. oceanensis* | CNT029 | 9 | 4 | | | | | | | 2 | | | 1 | | 1 | 1 | | | | |
| *S. oceanensis* | CNY703 | 9 | 4 | | | | | | | 2 | | | 1 | | 1 | 1 | | | | |
| *S. oceanensis* | CNY673 | 11 | 4 | | | | | 2 | | 2 | | | 1 | | 1 | 1 | | | | |
| *S. oceanensis* | CNT045 | 21 | 6 | | | | | | | 2 | | | 4 | 4 | 3 | 1 | 1 | | | |
| *S. oceanensis* | CNS996 | 23 | 6 | | | | | 2 | | 2 | | | 4 | 5 | 3 | 1 | | | | |
| *S. oceanensis* | CNT403 | 16 | 5 | | | | | 2 | | 2 | | | 2 | 2 | 2 | 1 | | | | |
| *S. oceanensis* | CNS860 | 15 | 5 | | | | | | 2 | 2 | | | 1 | 2 | 2 | 1 | | | | |
| *S. oceanensis* | CNS863_DSM45543 | 16 | 5 | | | | | | 2 | 2 | | | 2 | 2 | 2 | 1 | | | | |
| *S. goodfellowii* | CNY666 | 50 | 2 | | 1 | | 2 | | | 2 | | | 38 | 1 | 2 | 1 | | | 1 | |
| *S. vitiensis* | CNS055 | 20 | 1 | | 4 | | 2 | | | 2 | | | 9 | | 1 | 1 | | | | |
| *S. vitiensis* | CNT148_DSM45548 | 15 | 3 | | 5 | | 2 | | | 2 | | 1 | 1 | | 1 | | | | | |
| *S. vitiensis* | CNS801 | 15 | 3 | | 5 | | 2 | | | 2 | | 1 | 1 | | 1 | | | | | |
| *S. pacifica* | CNH732 | 20 | 2 | | 1 | | 2 | | | 2 | | | 9 | | 3 | 1 | | | | |
| *S. pacifica* | CNQ768 | 19 | 2 | | 1 | | 2 | | | 2 | | | 8 | | 3 | 1 | | | | |
| *S. pacifica* | CNR114 | 20 | 2 | | 1 | | 2 | | | 2 | | | 10 | 1 | 1 | 1 | | | | |
| *S. pacifica* | CNR510 | 23 | 2 | | 1 | | 2 | | | 2 | | | 11 | 1 | 3 | 1 | | | | |
| *S. pacifica* | CNR894 | 20 | 2 | | 1 | | 2 | | | 2 | | | 9 | | 3 | 1 | | | | |
| *S. pacifica* | CNR909 | 21 | 3 | | 1 | | 2 | | | 2 | | | 10 | | 1 | 1 | 1 | | | |
| *S. pacifica* | CNS103 | 23 | 3 | | 1 | | 2 | | | 2 | | | 11 | 1 | 2 | 1 | | | | |
| *S. pacifica* | CNT001 | 23 | 2 | | 1 | | 2 | | | 2 | | | 11 | 1 | 3 | 1 | | | | |
| *S. pacifica* | CNT003 | 28 | 3 | | 1 | | 2 | | | 2 | | | 16 | 1 | 1 | 1 | | | 1 | |
| *S. pacifica* | CNT084 | 28 | 4 | | 1 | | 2 | | | 2 | | | 14 | 2 | 1 | 1 | 1 | | | |
| *S. pacifica* | CNT131 | 30 | 2 | | 1 | | 2 | | | 2 | | | 17 | 1 | 3 | 1 | | | 1 | |
| *S. pacifica* | CNT133 | 29 | 4 | | 1 | | 2 | | | 2 | | | 15 | 2 | 1 | 1 | 1 | | | |
| *S. pacifica* | CNT603 | 20 | 2 | | 1 | | 2 | | | 2 | | | 9 | | 3 | 1 | | | | |
| *S. pacifica* | CNT609 | 26 | 3 | | 1 | | 2 | | | 2 | | 1 | 13 | 2 | 1 | 1 | | | | |
| *S. pacifica* | CNT796 | 21 | 2 | | 1 | | 2 | | 2 | 2 | | | 10 | | 1 | 1 | | | | |
| *S. pacifica* | CNT851 | 20 | 2 | | 1 | | 2 | | 2 | 2 | | | 9 | | 1 | 1 | | | | |
| *S. pacifica* | CNT855 | 21 | 2 | | 2 | | 2 | | | 2 | | 1 | 10 | | 1 | 1 | | | | |
| *S. pacifica* | CNY239 | 20 | 2 | | 1 | | 2 | | | 2 | | | 8 | | 4 | 1 | | | | |
| *S. pacifica* | CNY330 | 32 | 2 | | 1 | | 2 | | | 2 | | | 23 | | 1 | 1 | | | | |
| *S. pacifica* | CNY331 | 37 | 2 | | 2 | | 2 | | | 2 | | | 26 | 1 | 1 | 1 | | | | |
| *S. pacifica* | CNY363 | 45 | 2 | | 2 | | 2 | | | 2 | | | 33 | 1 | 1 | 1 | | | 1 | |
| *S. pacifica* | CNY498 | 22 | 2 | | 1 | | 2 | | | 2 | | | 10 | | 3 | 1 | | | 1 | |
| *S. pacifica* | CNS960_DSM45544 | 28 | 2 | | 2 | | 2 | | | 2 | | 1 | 14 | | 4 | 1 | | | | |
| *S. arenicola* | CNB458 | 24 | 2 | | 2 | | | | | 2 | | | 13 | | 1 | 2 | 2 | | | |
| *S. arenicola* | CNB527 | 29 | 2 | | 2 | | | | | 4 | | | 16 | | 1 | 2 | 2 | | | |
| *S. arenicola* | CNH643 | 36 | 3 | | 4 | | | | | 2 | | | 21 | 1 | 2 | 2 | 1 | | | |
| *S. arenicola* | CNH646 | 21 | 3 | | 4 | | | | | 2 | | | 7 | | 2 | 2 | 1 | | | |
| *S. arenicola* | CNH713 | 34 | 3 | | 4 | | | | | 2 | | | 19 | 1 | 2 | 2 | 1 | | | |
| *S. arenicola* | CNH718 | 33 | 2 | | 2 | | | | | 2 | | | 20 | 1 | 1 | 2 | 2 | 1 | | |
| *S. arenicola* | CNH877 | 29 | 3 | | 4 | | | | | 2 | | | 15 | | 2 | 2 | 1 | | | |
| *S. arenicola* | CNH905 | 28 | 3 | | 4 | | | | | 2 | | | 15 | | 1 | 2 | 1 | | | |
| *S. arenicola* | CNH941 | 23 | 3 | | 4 | | | | | 2 | | | 7 | 1 | 3 | 2 | 1 | | | |
| *S. arenicola* | CNH962 | 17 | 2 | | 2 | | | | | 2 | | | 8 | | | 2 | 1 | | | |
| *S. arenicola* | CNH963 | 18 | 2 | | 2 | | | | | 2 | | | 9 | | | 2 | 1 | | | |
| *S. arenicola* | CNH964 | 31 | 3 | 1 | 4 | | | | | 2 | | | 7 | 1 | 3 | 2 | 1 | | 6 | 1 |
| *S. arenicola* | CNH996B | 26 | 3 | | 2 | | | | | 2 | | | 13 | 1 | | 2 | 2 | 1 | | |
| *S. arenicola* | CNH996 | 26 | 3 | | 2 | | | | | 2 | | | 13 | 1 | | 2 | 2 | 1 | | |
| *S. arenicola* | CNP105 | 31 | 3 | 1 | 4 | | | | | 2 | | | 7 | 1 | 3 | 2 | 1 | | 6 | 1 |
| *S. arenicola* | CNP193 | 23 | 3 | | 4 | | | | | 2 | | | 7 | 1 | 3 | 2 | 1 | | | |
| *S. arenicola* | CNQ748 | 38 | 2 | | 2 | | | | | 2 | | | 24 | 1 | 2 | 2 | 2 | 1 | | |
| *S. arenicola* | CNQ884 | 50 | 2 | | 2 | | | | | 2 | | | 37 | 2 | 1 | 2 | 2 | | | |
| *S. arenicola* | CNR107 | 22 | 2 | | 3 | | | | | 2 | | | 8 | | 2 | 2 | 2 | 1 | | |
| *S. arenicola* | CNR425 | 33 | 2 | | 2 | | | | | 2 | | | 20 | 1 | 2 | 2 | 2 | | | |
| *S. arenicola* | CNR921 | 15 | 2 | | 2 | | | | | 2 | | | 4 | | 1 | 2 | 2 | | | |
| *S. arenicola* | CNS051 | 16 | 2 | | 2 | | | | | 2 | | | 5 | | 1 | 2 | 2 | | | |
| *S. arenicola* | CNS205 | 33 | 2 | | 2 | | | | | 2 | | | 20 | 1 | 1 | 2 | 2 | 1 | | |

# Table S5.

| | | | NaPDoS2: KS classes | | | | | | | | | | | | | | | | | |
|---|---|---|---|---|---|---|---|---|---|---|---|---|---|---|---|---|---|---|---|---|
| | | | FAS | Type II PKS | | | | | | | | | Type I PKS | | | | | | | |
| | | NP2 | | | | Aromatic | | | | | | | | cis-AT modular | | cis-AT iterative | | | trans-AT | |
| **Species** | **Strain** | **Total** | FAS II | Betabranch | Polyene | Angl-I | Angl-II | Anth-I | Isochrom | Polyphen | Unclass | TypeII-uc | *cis*-AT | cisloading | cisHybridKS | Ened | iPKSaromatic | PTM | *trans*-AT | *trans*-hybridKS0 |
| *S. arenicola* | CNS243 | 50 | 2 | | 2 | | | | | 2 | | | 36 | 2 | 1 | 2 | 2 | 1 | | |
| *S. arenicola* | CNS296 | 39 | 2 | | 2 | | | | | 2 | | | 24 | 2 | 2 | 2 | 2 | 1 | | |
| *S. arenicola* | CNS299 | 35 | 2 | | 2 | | | | | 2 | | | 22 | 2 | 1 | 2 | 2 | | | |
| *S. arenicola* | CNS325 | 35 | 2 | | 2 | | | | | 2 | 2 | | 19 | 2 | 2 | 2 | 2 | | | |
| *S. arenicola* | CNS342 | 32 | 2 | | 2 | | | | | 2 | | | 20 | 1 | 1 | 2 | 2 | | | |
| *S. arenicola* | CNS673 | 28 | 2 | | 2 | | | | | 2 | | | 15 | | 3 | 2 | 2 | | | |
| *S. arenicola* | CNS744 | 27 | 2 | | 2 | | | | | 4 | | | 12 | | 2 | 2 | 2 | 1 | | |
| *S. arenicola* | CNS820 | 34 | 2 | | 2 | | | | | 2 | | | 20 | 1 | 2 | 2 | 2 | 1 | | |
| *S. arenicola* | CNS848 | 38 | 3 | | 4 | | | | | 2 | | | 20 | 3 | 3 | 2 | 1 | | | |
| *S. arenicola* | CNT005 | 28 | 2 | | 2 | | | | | 2 | | | 16 | 1 | 1 | 2 | 2 | | | |
| *S. arenicola* | CNT798 | 28 | 3 | | 4 | | | | | 2 | | | 14 | | 2 | 2 | 1 | | | |
| *S. arenicola* | CNT799 | 34 | 3 | | 4 | | | | | 2 | | | 19 | 1 | 2 | 2 | 1 | | | |
| *S. arenicola* | CNT800 | 30 | 3 | | 4 | | | | | 2 | | | 16 | | 2 | 2 | 1 | | | |
| *S. arenicola* | CNT849 | 29 | 3 | | 4 | | | | | 2 | | | 15 | | 2 | 2 | 1 | | | |
| *S. arenicola* | CNT850 | 27 | 3 | | 4 | | | | | 2 | | | 13 | | 2 | 2 | 1 | | | |
| *S. arenicola* | CNT857 | 30 | 3 | | 4 | | | | | 2 | | | 16 | | 2 | 2 | 1 | | | |
| *S. arenicola* | CNT859 | 29 | 3 | | 4 | | | | | 2 | | | 16 | | 1 | 2 | 1 | | | |
| *S. arenicola* | CNX481 | 20 | 2 | | 2 | | | | | 2 | | | 8 | | 1 | 2 | 2 | 1 | | |
| *S. arenicola* | CNX482 | 19 | 2 | | 2 | | | | | 2 | | | 7 | | 1 | 2 | 2 | 1 | | |
| *S. arenicola* | CNX508 | 28 | 2 | | 2 | | | | | 2 | | | 16 | | 1 | 2 | 2 | 1 | | |
| *S. arenicola* | CNX814 | 21 | 2 | | 2 | | | 2 | | 2 | | | 7 | | 1 | 2 | 2 | 1 | | |
| *S. arenicola* | CNX891 | 20 | 2 | | 2 | | | 2 | | 2 | | | 6 | | 1 | 2 | 2 | 1 | | |
| *S. arenicola* | CNY011 | 30 | 3 | | 4 | | | | | 2 | | | 16 | | 2 | 2 | 1 | | | |
| *S. arenicola* | CNY230 | 40 | 2 | | 6 | | | | | 2 | | | 22 | 2 | 2 | 2 | 2 | | | |
| *S. arenicola* | CNY231 | 30 | 2 | | 2 | | | | | 2 | | | 18 | 1 | 1 | 2 | 2 | | | |
| *S. arenicola* | CNY234 | 25 | 2 | | 2 | | | | | 2 | | | 14 | | 1 | 2 | 2 | | | |
| *S. arenicola* | CNY237 | 25 | 2 | | 2 | | | | | 2 | | | 13 | | 1 | 2 | 2 | 1 | | |
| *S. arenicola* | CNY244 | 32 | 2 | | 2 | | | | | 2 | | | 19 | 1 | 1 | 2 | 2 | 1 | | |
| *S. arenicola* | CNY256 | 23 | 2 | | 2 | | | | | 2 | | | 12 | | 1 | 2 | 2 | | | |
| *S. arenicola* | CNY260 | 27 | 2 | | 2 | | | | | 2 | | | 15 | | 1 | 2 | 2 | 1 | | |
| *S. arenicola* | CNY280 | 40 | 3 | | 8 | | | | | 2 | | | 19 | 2 | 3 | 2 | 1 | | | |
| *S. arenicola* | CNY282 | 23 | 2 | | 2 | | | | | 2 | | | 12 | | 1 | 2 | 2 | | | |
| *S. arenicola* | CNY486 | 24 | 2 | | 2 | | | | | 2 | | | 13 | 1 | | 2 | 2 | | | |
| *S. arenicola* | CNY679 | 36 | 3 | | 4 | | | | | 2 | | | 21 | 1 | 2 | 2 | 1 | | | |
| *S. arenicola* | CNY685 | 31 | 2 | | 2 | | | | | 2 | | | 19 | 1 | 1 | 2 | 2 | | | |
| *S. arenicola* | CNY690 | 30 | 2 | | 2 | | | | | 2 | | | 18 | 1 | 1 | 2 | 2 | | | |
| *S. arenicola* | CNY694 | 30 | 2 | | 2 | | | | | 2 | | | 18 | 1 | 1 | 2 | 2 | | | |
| *S. arenicola* | CNS991_DSM45545 | 40 | 3 | | 4 | | | | | 2 | | | 24 | 2 | 2 | 2 | 1 | | | |
| | *total* | *3103* | *313* | *2* | *295* | *12* | *84* | *10* | *10* | *242* | *2* | *5* | *1490* | *92* | *200* | *186* | *104* | *18* | *36* | *2* |

**Table S6**. **KS domains identified in 27 fungal genomes by NaPDoS2.** Table lists the total number of KSs detected using default settings (e-value cutoff 1e-8 and 200aa minimum alignment length) and their class and subclass distributions. The summed total number of KSs found in each genome is listed as "Total NP2". The number of protein sequences in each analyzed genome file is listed as "# protein seq".

Abbreviations: bfFASI= type I FAS Bacterial-Fungal-type; FASII= type II FAS; *cis*-AT= type I modular *cis*-AT; cisHybridKS= type I modular *cis*-AT hybrid KS; PR= type I iterative *cis*-AT partially reducing; NR= type I iterative *cis*-AT non-reducing; HR= type I iterative *cis*-AT highly reducing.

**Table S6.**

| | | | | | Type I PKS | | | | |
|---|---|---|---|---|---|---|---|---|---|
| | | | | | *cis*-AT modular | | *cis*-AT iterative | | |
| **Fungal genome** | **# protein seq** | *Total NP2* | bfFASI | FASII | *cis*-AT | cisHybridKS | PR | NR | HR |
| *Aspergillus niger ATCC 1015* | 12,885 | 50 | 5 | 1 | 1 | - | 1 | 8 | 34 |
| *Aspergillus sergii CBS 130017* | 13,713 | 39 | 5 | 1 | - | - | 1 | 12 | 20 |
| *Aspergillus nidulans FGSC A4* | 9,556 | 37 | 5 | 1 | 1 | - | - | 14 | 16 |
| *Penicillium rolfsii F1880* | 9,955 | 26 | 2 | 1 | - | - | - | 6 | 17 |
| *Penicillium rubens Wisconsin 54-1255* | 12,791 | 25 | 2 | 1 | - | - | 2 | 3 | 17 |
| *Sclerotinia sclerotiorum 1980 UF-70* | 14,490 | 20 | 1 | 1 | - | - | - | 4 | 14 |
| *Leptosphaeria maculans JN3* | 12,469 | 17 | 2 | 1 | - | - | - | 4 | 10 |
| *Nectria haematococca MPVI isolate 77-13-4* | 15,708 | 15 | 1 | 1 | - | - | - | 2 | 11 |
| *Alternaria alternata SRC1lrK2f* | 13,466 | 14 | 1 | 1 | - | 1 | - | 3 | 8 |
| *Zymoseptoria tritici IPO323* | 10,941 | 14 | 1 | 1 | - | - | - | 2 | 10 |
| *Neurospora crassa OR74A* | 10,812 | 11 | 2 | 1 | - | - | - | 1 | 7 |
| *Arthrobotrys oligospora TWF154* | 13,042 | 9 | 1 | 1 | - | - | 1 | - | 6 |
| *Ustilago maydis 521* | 6,782 | 5 | 2 | - | - | - | - | 3 | - |
| *Allomyces macrogynus ATCC 38327* | 19,447 | 4 | 2 | 2 | - | - | - | - | - |
| *Laccaria bicolor S238N-H82* | 18,215 | 3 | 1 | - | 1 | - | - | 1 | - |
| *Mucor circinelloides 1006PhL* | 12,227 | 3 | 2 | 1 | - | - | - | - | - |
| *Coprinopsis cinerea okayama 7#130 CC3* | 13,356 | 2 | 1 | - | - | - | - | 1 | - |
| *Pyronema omphalodes CBS 100304* | 13,367 | 3 | 1 | 1 | - | - | - | 1 | - |
| *Schizophyllum commune H4-8* | 13,193 | 3 | 2 | - | - | - | - | 1 | - |
| *Batrachochytrium dendrobatidis JAM81* | 8,677 | 2 | 1 | 1 | - | - | - | - | - |
| *Debaryomyces hansenii CBS767* | 6,286 | 2 | 1 | 1 | - | - | - | - | - |
| *Neolecta irregularis DAH-3* | 5,579 | 2 | 1 | 1 | - | - | - | - | - |
| *Puccinia graminis tritici CRL 75-36-700-3* | 15,979 | 2 | 2 | - | - | - | - | - | - |
| *Saccharomyces cerevisiae S288C* | 6,002 | 2 | 1 | 1 | - | - | - | - | - |
| *Saitoella complicata NRRL Y-17804* | 7,023 | 2 | 1 | 1 | - | - | - | - | - |
| *Schizosaccharomyces pombe 972h-* | 5,132 | 2 | 1 | 1 | - | - | - | - | - |
| *Malassezia globosa CBS 7966* | 4,286 | 1 | - | - | 1 | - | - | - | - |

**Table S7. KS detection using NaPDoS versions 1 and 2.**
MIBiG Fungal PKS: NaPDoS2 detected all of the KS domains in 159 MIBiG 2.0[5] fungal PKS BGCs. NaPDoS version 1 and 2 analyses were run with the default settings.
Wawrik 2005 KS clones: While both NaPDoS versions 1 and 2 detected all 147 type II KS amplicons from Wawrik *et al*. 2005[18], NaPDoS2 could further delineate these sequences into three subclasses. NaPDoS version 1 and 2 analyses were run with the following settings: NaPDoS version 1: HMM 1e-5, 200aa minimum alignment length, pathway assignment: e-value cutoff of 1e-5; NaPDoS2: e-value cutoff 1e-8 and 50aa minimum alignment length. The summed total number of KSs found in each genome is listed as “Total NP1” and “Total NP2” for NaPDoS versions 1 and 2, respectively.

Abbreviations: Iter= Iterative type I; FA= fatty acid synthase; bfFASI= type I FAS Bacterial-Fungal-type; *cis*-AT= type I modular *cis*-AT; PR= type I iterative *cis*-AT partially reducing; NR= type I iterative *cis*-AT non-reducing; HR= type I iterative *cis*-AT highly reducing; Aro-KSα= type II aromatic unclassified KSα; Angl-I-KSα= type II aromatic angucycline-derived I KSα; Polyphen-KSα= type II aromatic pentangular polyphenol-derived KSα.

**Table S7.**

| | **NaPDoS1** | | | | **NaPDoS2** | | Type I PKS | | | | Type II PKS | | |
|---|---|---|---|---|---|---|---|---|---|---|---|---|---|
| | | | | | | | *cis*-AT modular | *cis*-AT iterative | | | Aromatic | | |
| **Sequence collection** | *Total NP1* | Type II | Iter | FA | *Total NP2* | bfFASI | *cis*-AT | PR | NR | HR | Aro-KSα | Angl-I-KSα | Polyphen-KSα |
| MIBiG Fungal PKS (159 BGCs) | 14 | - | 7 | 7 | 182 | 10 | 4 | 7 | 71 | 90 | - | - | - |
| Wawrik 2005 KS clones (147 seqs) | 147 | 147 | - | - | 147 | - | - | - | - | - | 1 | 45 | 101 |

**Table S8**. **NaPDoS2 analysis of the *Elysia chlorotica* genome.** NaPDoS2 identified nine KSs from the *Elysia chlorotica* CDSs (fna), protein (faa), and translated CDSs (faa) genomes[19]. Highlighted KSs were identified in Torres *et al*. 2020[20] as being associated with new FAS-like animal PKSs (EcPKS1, EcPKS2) and an FAS (EcFAS). The analyses were run with NaPDoS2 default settings (e-value cutoff 1e-8 and 200aa minimum alignment length).

**Table S8.**

| Match | Query ID | Database match ID | % ID | Align length | E-value | BGC Match | Domain Class | Domain Subclass |
|---|---|---|---|---|---|---|---|---|
| **EcFAS** | RUS90834.1 | OryziasFAS_KS01_MetazoaFASI | 60 | 403 | 5.7e-152 | Oryzias latipes FAS | type I FAS | Metazoan-type |
| **EcPKS1** | RUS71288.1 | HomoFAS_KS01_MetazoaFASI | 52 | 406 | 9.6e-119 | Homo sapiens FAS | type I FAS | Metazoan-type |
| | RUS77019.1 | OryziasFAS_KS01_MetazoaFASI | 49 | 403 | 2.7e-117 | Oryzias latipes FAS | type I FAS | Metazoan-type |
| **EcPKS2** | RUS75294.1 | OryziasFAS_KS01_MetazoaFASI | 49 | 403 | 4.7e-117 | Oryzias latipes FAS | type I FAS | Metazoan-type |
| | RUS92164.1 | MelopsittacusFAS_KS01_MetazoaFASI | 39 | 410 | 2.9e-76 | Melopsittacus undulatus FAS | type I FAS | Metazoan-type |
| | RUS75295.1 | OryziasFAS_KS01_MetazoaFASI | 45 | 308 | 6.3e-73 | Oryzias latipes FAS | type I FAS | Metazoan-type |
| | RUS68442.1 | AliivibrioAPE_KS03_FASII | 60 | 407 | 1.3e-137 | Aliivibrio fischeri aryl polyene | type II FAS | no subclass |
| | RUS69056.1 | EscherichiaFAS_KS02_FASII | 57 | 420 | 1.9e-136 | Escherichia coli FAS | type II FAS | no subclass |
| | RUS77541.1 | EscherichiaFAS_KS02_FASII | 47 | 421 | 4.8e-103 | Escherichia coli FAS | type II FAS | no subclass |

**Table S9. Moorea sediment metagenomes analyzed with NaPDoS2.** KS domains from 20 marine sediment metagenomes[21] were assigned to 26 different subclasses. NaPDoS2 analyses were run with default settings (e-value cutoff 1e-8 and 200aa minimum alignment length). KSα and KSβ sequences are grouped together.

Abbreviations: MetazoaFASI= type I FAS Metazoan-type; ProtistFASI= type I FAS Protist-type; FASII= type II FAS no subclass; Betabranch= type II beta-branching cassettes; Polyene= type II polyenes; Ape= type II aryl polyenes; Aro= type II aromatic unclassified; Angl= type II aromatic angucycline-derived I & II; Tetcyc= type II aromatic tetracycline-derived; Anth= type II aromatic anthracycline-derived I & II; Isochrom= type II aromatic isochromanequinone-derived; Tetcen= type II aromatic tetracenomycin-derived; Polyphen= type II aromatic pentangular polyphenol-derived; SPKS= type II aromatic spore pigment; *cis*-AT= type I modular *cis*-AT; cisloading= type I modular *cis*-AT loading module; cisHybridKS= type I modular *cis*-AT hybrid KS; cisOLS= type I modular *cis*-AT olefin synthase; cistandemECH= type I modular *cis*-AT tandem ECH; iPKS= type I iterative *cis*-AT no subclass; iPKSPUFA= type I iterative *cis*-AT PUFA (polyunsaturated fatty acids); Ened= type I iterative *cis*-AT enediynes; iPKSaromatic= type I iterative *cis*-AT aromatic; PTM= type I iterative *cis*-AT PTM-type (polycyclic tetramate macrolactam); HR= type I iterative *cis*-AT highly reducing; *trans*-AT= type I *trans*-AT no subclass; *trans*-hybridKS= type I *trans*-AT hybrid KS; *trans*-hybridKS0= type I *trans*-AT hybrid KS0 (non-elongating KS).

# Table S9.

| | | | | | | | | Type II PKS | | | | | | | | | | | Type I PKS | | | | | | | | | | | | | |
|---|---|---|---|---|---|---|---|---|---|---|---|---|---|---|---|---|---|---|---|---|---|---|---|---|---|---|---|---|---|---|---|---|
| | | | | FAS I | | | | | | | Aromatic | | | | | | | | *cis*-AT modular | | | | | *cis*-AT iterative | | | | | | *trans*-AT | | |
| **Metagenome** | ***# contigs*** | ***N50*** | ***Total NP2*** | MetazoaFASI | ProtistFASI | FASII | MetazoaPKS | Betabranch | Polyene | Ape | Aro | Angl | Tetcyc | Anth | Isochrom | Tetcen | Polyphen | SPKS | *cis*-AT | cisloading | cisHybridKS | cisOLS | cistandemECH | iPKS | iPKSPUFA | Ened | iPKSaromatic | PTM | HR | *trans*-AT | *trans*-hybridKS | *trans*-hybridKS0 |
| MO18_007 | 152,894 | 5,918 | 145 | - | - | 61 | - | - | 5 | 3 | 1 | 5 | - | - | - | - | - | - | 19 | 3 | 5 | 1 | - | 1 | 34 | - | - | - | - | 7 | - | - |
| MO18_010 | 184,034 | 3,212 | 150 | - | - | 54 | - | 1 | 4 | 5 | 2 | 2 | - | 1 | 1 | 1 | - | - | 17 | 1 | 14 | - | - | 3 | 34 | 1 | - | - | - | 9 | - | - |
| MO18_039 | 336,958 | 1,769 | 58 | - | - | 13 | 4 | - | 1 | 1 | - | 1 | - | - | 1 | - | - | - | 8 | - | 17 | - | - | - | 3 | 1 | - | - | - | 8 | - | - |
| MO18_042 | 378,383 | 1,596 | 121 | - | - | 60 | - | 1 | 1 | 5 | 1 | 2 | - | - | - | - | - | - | 13 | 3 | 3 | 2 | - | 2 | 22 | - | - | 1 | - | 5 | - | - |
| MO18_071 | 37,150 | 2,093 | 87 | - | 1 | 23 | - | - | - | 2 | - | 1 | - | - | - | - | 1 | - | 24 | - | 9 | 3 | - | - | 5 | - | - | 1 | - | 16 | - | 1 |
| MO18_074 | 211,343 | 2,041 | 312 | - | - | 149 | 1 | - | 5 | 8 | 6 | 6 | - | - | - | 1 | 4 | 1 | 41 | 1 | 28 | 3 | - | 8 | 39 | 4 | - | - | 1 | 6 | - | - |
| MO18_103 | 186,468 | 1,851 | 269 | - | 1 | 114 | - | 1 | 6 | 13 | 3 | 1 | - | - | 1 | 1 | 6 | - | 34 | 3 | 15 | 3 | 1 | 4 | 46 | 3 | - | - | - | 13 | - | - |
| MO18_106 | 440,441 | 1,847 | 48 | - | - | 12 | - | - | - | - | - | - | - | - | - | - | - | - | 19 | 1 | - | 6 | - | - | 7 | - | - | - | - | 3 | - | - |
| MO18_135 | 230,831 | 2,120 | 270 | - | 1 | 91 | - | 4 | 1 | 5 | 4 | 5 | 1 | 1 | - | - | 2 | - | 34 | 2 | 35 | 6 | 3 | 2 | 32 | 6 | - | - | - | 35 | - | - |
| MO18_138 | 104 | 1,300 | 0 | - | - | - | - | - | - | - | - | - | - | - | - | - | - | - | - | - | - | - | - | - | - | - | - | - | - | - | - | - |
| MO18_167 | 311,846 | 2,402 | 706 | - | - | 272 | - | 7 | 5 | 21 | 12 | 13 | - | 4 | 1 | 1 | 8 | 1 | 83 | 11 | 48 | 18 | 3 | 8 | 129 | 18 | 2 | 1 | - | 39 | 1 | - |
| MO18_170 | 234,614 | 1,716 | 336 | - | - | 192 | - | 1 | 5 | 20 | 6 | 7 | - | 1 | 1 | 2 | 2 | - | 16 | 1 | 8 | 2 | - | 3 | 60 | 6 | - | 1 | - | 2 | - | - |
| MO18_180 | 182,647 | 1,599 | 227 | - | - | 125 | - | 1 | 5 | 12 | - | 4 | - | - | 1 | - | 3 | - | 13 | 1 | 10 | 4 | 1 | 5 | 29 | 3 | - | - | - | 10 | - | - |
| MO18_184 | 309,098 | 3,457 | 51 | - | - | 11 | - | 1 | - | 3 | 1 | 1 | - | - | - | - | 1 | - | 17 | 1 | - | 5 | - | - | 5 | - | - | - | - | 5 | - | - |
| MO18_188 | 259,773 | 2,959 | 505 | - | - | 188 | 1 | 5 | 5 | 9 | 4 | 10 | 1 | - | - | - | 7 | - | 106 | 4 | 41 | 20 | 3 | 7 | 53 | 8 | 1 | 1 | - | 30 | 1 | - |
| MO18_192 | 241,995 | 1,727 | 341 | - | - | 116 | 2 | 4 | 8 | 8 | 2 | 5 | - | 3 | - | - | 4 | - | 60 | 3 | 34 | 10 | 2 | 3 | 49 | 8 | - | - | - | 20 | - | - |
| MO18_199 | 229,239 | 2,042 | 200 | 3 | - | 77 | - | 4 | 2 | 3 | 1 | 2 | - | 1 | 1 | - | 2 | - | 46 | 3 | 2 | 1 | 4 | 3 | 31 | 2 | - | - | - | 12 | - | - |
| MO18_202 | 242,491 | 2,136 | 280 | - | 1 | 122 | - | 2 | 5 | 10 | 5 | 8 | - | - | - | 1 | 3 | - | 41 | 3 | 14 | 2 | 3 | 3 | 48 | 2 | - | - | - | 7 | - | - |
| MO18_231 | 110,677 | 2,357 | 91 | - | - | 30 | - | 1 | - | 5 | - | 2 | - | - | - | - | 1 | - | 22 | 1 | 2 | - | 1 | - | 20 | 1 | - | - | - | 5 | - | - |
| MO18_234 | 182,155 | 2,286 | 170 | - | 3 | 62 | - | 2 | - | - | 2 | 4 | - | - | - | - | - | - | 37 | - | 13 | 6 | 1 | 1 | 20 | 2 | - | - | - | 16 | 1 | - |

**Table S10. NaPDoS2 analysis of KS amplicon eSNaPD v2.0 data.** NaPDoS2 detected and classified all 381 sequences from a New Mexico desert soil KS amplicon library (NM_KS_ARRY_LIB01, Owen *et al.* 2013 PNAS[22]). Additionally, NaPDoS2 classified all but one group of the 756 uncharacterized "Novel Clusters 1-60" from the same eSNaPD v2.0 soil amplicon library. NaPDoS2 settings: e-value cutoff 1e-8 and 50aa minimum alignment length (due to the amplicon sequence length). The "% ID" column lists the percent of sequences that were classified by NaPDoS2 (i.e. "Total NP2"/"# seq"; color scale of light grey (low % ID) to dark grey (high % ID)).

Abbreviations: *cis*-AT= type I modular *cis*-AT; cisloading= type I modular *cis*-AT loading module; cisHybridKS= type I modular *cis*-AT hybrid KS; cisOLS= type I modular *cis*-AT olefin synthase; cistandemECH= type I modular *cis*-AT tandem ECH; iPKSaromatic= type I iterative *cis*-AT aromatic; PTM= type I iterative *cis*-AT PTM-type (polycyclic tetramate macrolactam); NR= type I iterative *cis*-AT non-reducing; HR= type I iterative *cis*-AT highly reducing; *trans*-AT= type I *trans*-AT no subclass; *trans*-hybridKS= type I *trans*-AT hybrid KS.

# Table S10.

| % ID | Sequence set | # seq | *Total NP2* | Type I PKS | | | | | | | | | | |
|---|---|---|---|---|---|---|---|---|---|---|---|---|---|---|
| | | | | *cis*-AT modular | | | | | *cis*-AT iterative | | | | *trans*-AT | |
| | | | | *cis*-AT | cisloading | cisHybridKS | cisOLS | cistandemECH | iPKSaromatic | PTM | NR | HR | *trans*-AT | *trans*-hybridKS |
| 102% | NM_KS_ARRY_LIB01 | 381 | 388 | 310 | 7 | 15 | 2 | - | 5 | 13 | - | - | 36 | - |
| 99% | NovClst1 | 192 | 191 | 2 | - | - | - | - | - | - | 189 | - | - | - |
| 100% | NovClst2 | 42 | 42 | 4 | 15 | - | 15 | - | - | 4 | - | 4 | - | - |
| 100% | NovClst3 | 38 | 38 | 3 | - | - | - | - | - | 35 | - | - | - | - |
| 94% | NovClst4 | 31 | 29 | - | - | - | - | - | - | - | - | - | 29 | - |
| 79% | NovClst5 | 29 | 23 | - | - | 22 | - | - | - | - | - | - | - | 1 |
| 108% | NovClst6 | 25 | 27 | - | - | - | - | - | - | - | 27 | - | - | - |
| 100% | NovClst7 | 22 | 22 | - | - | 22 | - | - | - | - | - | - | - | - |
| 112% | NovClst8 | 17 | 19 | - | - | 18 | - | - | - | - | - | - | - | 1 |
| 86% | NovClst9 | 14 | 12 | - | - | 11 | - | - | - | - | - | - | - | 1 |
| 92% | NovClst10 | 12 | 11 | 8 | - | - | - | - | - | - | - | - | 3 | - |
| 100% | NovClst11 | 12 | 12 | 6 | - | - | - | - | - | - | - | - | 6 | - |
| 100% | NovClst12 | 12 | 12 | - | - | - | - | - | - | 12 | - | - | - | - |
| 92% | NovClst13 | 12 | 11 | - | - | 11 | - | - | - | - | - | - | - | - |
| 110% | NovClst14 | 10 | 11 | 4 | - | - | 3 | - | - | - | - | - | 4 | - |
| 100% | NovClst15 | 10 | 10 | - | - | - | 10 | - | - | - | - | - | - | - |
| 100% | NovClst16 | 9 | 9 | - | - | 9 | - | - | - | - | - | - | - | - |
| 89% | NovClst17 | 9 | 8 | 7 | - | - | 1 | - | - | - | - | - | - | - |
| 111% | NovClst18 | 9 | 10 | 10 | - | - | - | - | - | - | - | - | - | - |
| 78% | NovClst19 | 9 | 7 | 5 | - | - | - | 1 | - | - | - | - | 1 | - |
| 89% | NovClst20 | 9 | 8 | - | - | 7 | - | - | - | - | - | - | - | 1 |
| 89% | NovClst21 | 9 | 8 | 8 | - | - | - | - | - | - | - | - | - | - |
| 100% | NovClst22 | 8 | 8 | - | - | - | - | - | - | - | 8 | - | - | - |
| 113% | NovClst23 | 8 | 9 | 9 | - | - | - | - | - | - | - | - | - | - |
| 113% | NovClst24 | 8 | 9 | 1 | - | - | - | - | - | - | - | 8 | - | - |
| 88% | NovClst25 | 8 | 7 | - | - | - | - | - | - | - | - | - | 7 | - |
| 100% | NovClst26 | 8 | 8 | - | - | 8 | - | - | - | - | - | - | - | - |
| 100% | NovClst27 | 8 | 8 | 8 | - | - | - | - | - | - | - | - | - | - |
| 114% | NovClst28 | 7 | 8 | - | - | - | - | - | - | - | 8 | - | - | - |
| 86% | NovClst29 | 7 | 6 | - | - | 6 | - | - | - | - | - | - | - | - |
| 100% | NovClst30 | 7 | 7 | - | - | 7 | - | - | - | - | - | - | - | - |
| 100% | NovClst31 | 7 | 7 | 4 | - | - | 3 | - | - | - | - | - | - | - |
| 100% | NovClst32 | 7 | 7 | - | - | 7 | - | - | - | - | - | - | - | - |
| 86% | NovClst33 | 7 | 6 | - | - | - | - | - | - | - | - | - | 6 | - |
| 100% | NovClst34 | 7 | 7 | - | - | 7 | - | - | - | - | - | - | - | - |
| 100% | NovClst35 | 6 | 6 | 1 | 1 | - | - | - | - | - | - | - | 4 | - |
| 67% | NovClst36 | 6 | 4 | - | - | - | - | - | - | - | - | - | 4 | - |
| 100% | NovClst37 | 6 | 6 | - | - | 6 | - | - | - | - | - | - | - | - |
| 83% | NovClst38 | 6 | 5 | - | - | 5 | - | - | - | - | - | - | - | - |
| 100% | NovClst39 | 6 | 6 | 6 | - | - | - | - | - | - | - | - | - | - |
| 67% | NovClst40 | 6 | 4 | - | - | 4 | - | - | - | - | - | - | - | - |
| 100% | NovClst41 | 6 | 6 | 6 | - | - | - | - | - | - | - | - | - | - |
| 0% | NovClst42 | 6 | 0 | - | - | - | - | - | - | - | - | - | - | - |
| 100% | NovClst43 | 6 | 6 | 1 | - | - | 5 | - | - | - | - | - | - | - |
| 100% | NovClst44 | 5 | 5 | - | - | - | - | - | - | - | - | - | 5 | - |
| 100% | NovClst45 | 5 | 5 | - | - | 5 | - | - | - | - | - | - | - | - |
| 100% | NovClst46 | 5 | 5 | 5 | - | - | - | - | - | - | - | - | - | - |
| 140% | NovClst47 | 5 | 7 | 2 | - | - | 4 | - | - | - | - | 1 | - | - |
| 60% | NovClst48 | 5 | 3 | - | - | - | - | - | - | - | - | - | 3 | - |
| 100% | NovClst49 | 4 | 4 | - | - | 4 | - | - | - | - | - | - | - | - |
| 100% | NovClst50 | 4 | 4 | - | - | 4 | - | - | - | - | - | - | - | - |
| 100% | NovClst51 | 4 | 4 | - | - | 4 | - | - | - | - | - | - | - | - |
| 100% | NovClst52 | 4 | 4 | - | - | - | - | - | - | - | - | - | 4 | - |
| 25% | NovClst53 | 4 | 1 | 1 | - | - | - | - | - | - | - | - | - | - |
| 100% | NovClst54 | 4 | 4 | 4 | - | - | - | - | - | - | - | - | - | - |
| 100% | NovClst55 | 4 | 4 | - | - | 4 | - | - | - | - | - | - | - | - |
| 100% | NovClst56 | 4 | 4 | - | - | - | - | - | - | - | - | - | 4 | - |
| 100% | NovClst57 | 4 | 4 | - | - | 4 | - | - | - | - | - | - | - | - |
| 75% | NovClst58 | 4 | 3 | 3 | - | - | - | - | - | - | - | - | - | - |
| 75% | NovClst59 | 4 | 3 | - | - | 3 | - | - | - | - | - | - | - | - |
| 175% | NovClst60 | 4 | 7 | - | - | - | - | - | 5 | - | 7 | - | 36 | - |

**Table S11**. **NaPDoS2 analysis of 12 type II KS amplicon datasets from Borsetto *et al.* 2019[23].** The total number of amplicon sequences not detected by NaPDoS2 represented 36-95% of the sequences in the amplicon libraries ("# seqs"). Of the KS sequences that were detected by NaPDoS2, 19-93% were classified as type II PKSs and could be assigned to a wide range of type II subclasses while the remainder were classified as type II fatty acid synthases (FASII). NaPDoS2 settings: e-value cutoff 1e-8 and 50aa minimum alignment length (due to the amplicon sequence length).

Abbreviations: FASII= type II FAS no subclass; Betabranch= type II beta-branching cassettes; Polyene-KSα= type II polyenes KSα; Ape-KSα= type II aryl polyenes KSα; Aro-KSα= type II aromatic unclassified KSα; Angl-I-KSα= type II aromatic angucycline-derived I KSα; Angl-II-KSα= type II aromatic angucycline-derived II KSα; Tetcyc-KSα= type II aromatic tetracycline-derived KSα; Anth-I-KSα= type II aromatic anthracycline-derived I KSα; Anth-I-KSβ= type II aromatic anthracycline-derived I KSβ; Anth-II-KSα= type II aromatic anthracycline-derived II KSα; Isochrom-KSα= type II aromatic isochromanequinone-derived KSα; Tetcen-KSα= type II aromatic tetracenomycin-derived KSα; Tetcen-KSβ= type II aromatic tetracenomycin-derived KSβ; Polyphen-KSα= type II aromatic pentangular polyphenol-derived KSα; Polyphen-KSβ= type II aromatic pentangular polyphenol-derived KSβ; SPKS-KSα= type II aromatic spore pigment KSα; *cis*-AT= type I modular *cis*-AT; cisHybridKS= type I modular *cis*-AT hybrid KS.

# Table S11.

| | | | | Type II PKS | | | | | | | | | | | | | | | | Type I PKS | |
|---|---|---|---|---|---|---|---|---|---|---|---|---|---|---|---|---|---|---|---|---|---|
| | | | | | | | Aromatic | | | | | | | | | | | | | *cis*-AT modular | |
| **Dataset** | **# seqs** | ***Total NP2*** | FASII | Betabranch | Polyene-KSα | Ape-KSα | Aro-KSα | Angl-I-KSα | Angl-II-KSα | Tetcyc-KSα | Anth-I-KSα | Anth-I-KSβ | Anth-II-KSα | Isochrom-KSα | Tetcen-KSα | Tetcen-KSβ | Polyphen-KSα | Polyphen-KSβ | SPKS-KSα | *cis*-AT | cisHybridKS |
| S31_Antarctica | 34,600 | 12,757 | 9,593 | 6 | 54 | 2 | 63 | 419 | 163 | 13 | 10 | 1 | 21 | 201 | 75 | 3 | 333 | 20 | 1,780 | - | - |
| S32_Antarctica | 31,118 | 7,905 | 6,414 | 4 | 50 | 12 | 52 | 369 | 51 | 13 | 42 | 1 | 11 | 134 | 47 | 1 | 343 | 16 | 344 | 1 | - |
| S33_Antarctica | 14,703 | 774 | 328 | - | 8 | - | 18 | 264 | 1 | 1 | 20 | - | - | 31 | 30 | 2 | 18 | 9 | 44 | - | - |
| S22_AlgeriaB3 | 166,814 | 9,345 | 1,172 | 1 | 177 | 15 | 1,253 | 1,003 | 37 | 4 | 2,573 | - | 76 | 366 | 461 | - | 1,370 | - | 836 | 1 | - |
| S23_AlgeriaB3 | 119,567 | 19,317 | 3,155 | 4 | 429 | 38 | 836 | 4,648 | 22 | 39 | 4,687 | - | 106 | 418 | 954 | - | 1,212 | - | 2,768 | 1 | - |
| S24_AlgeriaB3 | 126,771 | 21,643 | 3,072 | - | 373 | 26 | 1,354 | 4,149 | 27 | 4 | 6,824 | - | 148 | 414 | 1,075 | - | 2,097 | - | 2,080 | - | - |
| S28_CubaFir | 182,418 | 13,220 | 3,605 | - | 308 | 17 | 931 | 2,718 | 9 | 10 | 1,174 | - | 90 | 851 | 48 | - | 527 | - | 2,931 | 1 | - |
| S29_CubaFir | 194,450 | 18,079 | 2,394 | - | 146 | 12 | 1,279 | 7,113 | 8 | 3 | 3,331 | - | 18 | 1,174 | 18 | - | 896 | - | 1,687 | - | - |
| S30_CubaFir | 204,614 | 30,393 | 2,701 | - | 1,056 | 11 | 993 | 7,993 | 6 | 8 | 1,822 | - | 194 | 4,820 | 39 | - | 973 | - | 9,768 | 9 | - |
| S37_Warwick | 241,475 | 115,324 | 19,560 | 8 | 560 | 30 | 3,964 | 15,941 | 160 | 20 | 6,114 | - | 269 | 38,472 | 38 | - | 8,868 | 17 | 21,276 | 23 | 4 |
| S38_Warwick | 110,892 | 58,876 | 7,728 | 4 | 321 | 22 | 4,390 | 6,556 | 124 | 1 | 3,158 | - | 269 | 7,026 | 38 | - | 4,913 | 11 | 24,312 | 1 | 2 |
| S39_Warwick | 135,287 | 86,200 | 5,716 | 1 | 118 | 10 | 2,760 | 12,604 | 344 | 6 | 2,936 | - | 600 | 33,933 | 60 | - | 4,122 | 15 | 22,974 | - | 1 |

**Table S12**. **NaPDoS2 analysis of 5,000 randomly selected KS amplicon sequences from the Elfeki *et al.* 2018[15] "Chitin" type I** (SRR7206837_H054B_Chitin_KSdomain) **and type II** (SRR7206805_H054B_Chitin_KSalphadomain) **KS datasets run at varying minimum amino acid alignment lengths.** Decreasing the minimum amino acid alignment length increases the number of KSs detected but also increases the likelihood of false positives and misclassifications, as may be evidenced by the detection of type I KSs in the type II dataset. Notably, below an alignment length of 100aa, the number of KSs detected in the type I dataset exceeds the number of sequences analyzed, as can be expected when shorter domain fragment hits are identified from the same longer amplicon sequences.

**Table S12.**

| Type II KSα: Chitin | | | | |
|---|---|---|---|---|
| Alignment Length | Class | Subclass | NP2 Hits | *Total NP2 hits* |
| **200aa** | - | - | 0 | 0 |
| **150aa** | type II aromatic | pentangularpolyphenolKSa | 1 | 1 |
| **100aa** | type II aromatic | angucycline I KSa | 469 | 1,229 |
| | type II aromatic | isochromanequinone KSa | 225 | |
| | type II aromatic | pentangular polyphenol KSa | 200 | |
| | type II aromatic | unclassified KSa | 146 | |
| | type II aromatic | tetracenomycin KSa | 111 | |
| | type II aromatic | anthracycline I KSa | 70 | |
| | type I modular cis-AT | no subclass | 2 | |
| | type II aromatic | angucycline II KSa | 2 | |
| | type II aromatic | spore pigment KSa | 1 | |
| | type I trans-AT | hybrid KS | 1 | |
| | type I trans-AT | no subclass | 1 | |
| | type II aromatic | anthracycline II KSa | 1 | |
| **50aa** | type II aromatic | angucycline I KSa | 958 | 2,999 |
| | type II aromatic | pentangular polyphenol KSa | 673 | |
| | type II aromatic | isochromanequinone KSa | 570 | |
| | type II aromatic | unclassified KSa | 378 | |
| | type II aromatic | tetracenomycin KSa | 221 | |
| | type II aromatic | anthracycline I KSa | 132 | |
| | type II aromatic | spore pigment KSa | 31 | |
| | type I modular cis-AT | no subclass | 20 | |
| | type II aromatic | anthracycline II KSa | 4 | |
| | type II aromatic | tetracycline KSa | 4 | |
| | type II aromatic | angucycline II KSa | 4 | |
| | type I modular cis-AT | hybrid KS | 2 | |
| | type I trans-AT | hybrid KS | 1 | |
| | type I trans-AT | no subclass | 1 | |
| **30aa** | type II aromatic | angucycline I KSa | 994 | 3,099 |
| | type II aromatic | pentangular polyphenol KSa | 693 | |
| | type II aromatic | isochromanequinone KSa | 584 | |
| | type II aromatic | unclassified KSa | 381 | |
| | type II aromatic | tetracenomycin KSa | 224 | |
| | type II aromatic | anthracycline I KSa | 138 | |
| | type II aromatic | spore pigment KSa | 40 | |
| | type I modular cis-AT | no subclass | 22 | |
| | type II aromatic | tetracycline KSa | 9 | |
| | type II aromatic | anthracycline II KSa | 5 | |
| | type II aromatic | angucycline II KSa | 5 | |
| | type I modular cis-AT | hybrid KS | 2 | |
| | type I trans-AT | hybrid KS | 1 | |
| | type I trans-AT | no subclass | 1 | |

| Type I KS: Chitin | | | | |
|---|---|---|---|---|
| Alignment Length | Class | Subclass | NP2 Hits | *Total NP2 hits* |
| **200aa** | type I modular cis-AT | no subclass | 21 | 23 |
| | type I iterative cis-AT | aromatic | 2 | |
| **150aa** | type I modular cis-AT | no subclass | 385 | 423 |
| | type I iterative cis-AT | aromatic | 24 | |
| | type I trans-AT | no subclass | 8 | |
| | type I trans-AT | hybrid KS | 6 | |
| **100aa** | type I modular cis-AT | no subclass | 3,406 | 3,594 |
| | type I trans-AT | no subclass | 75 | |
| | type I iterative cis-AT | aromatic | 57 | |
| | type I modular cis-AT | hybrid KS | 39 | |
| | type I trans-AT | hybrid KS | 15 | |
| | type II aromatic | pentangular polyphenol KSa | 1 | |
| | type II aromatic | spore pigment KSa | 1 | |
| **50aa** | type I modular cis-AT | no subclass | 7,441 | 7,768 |
| | type I trans-AT | no subclass | 139 | |
| | type I modular cis-AT | hybrid KS | 85 | |
| | type I iterative cis-AT | aromatic | 72 | |
| | type I trans-AT | hybrid KS | 17 | |
| | type II aromatic | pentangular polyphenol KSa | 5 | |
| | type II aromatic | angucycline I KSa | 3 | |
| | type II aromatic | spore pigment KSa | 2 | |
| | type II aromatic | anthracycline I KSa | 1 | |
| | type II aromatic | isochromanequinone KSa | 1 | |
| | type II aromatic | tetracenomycin KSa | 1 | |
| | type II aromatic | unclassified KSa | 1 | |
| **30aa** | type I modular cis-AT | no subclass | 7,600 | 7,930 |
| | type I trans-AT | no subclass | 140 | |
| | type I modular cis-AT | hybrid KS | 86 | |
| | type I iterative cis-AT | aromatic | 72 | |
| | type I trans-AT | hybrid KS | 17 | |
| | type II aromatic | pentangular polyphenol KSa | 5 | |
| | type II aromatic | angucycline I KSa | 3 | |
| | type II aromatic | spore pigment KSa | 3 | |
| | type II aromatic | anthracycline I KSa | 1 | |
| | type II aromatic | isochromanequinone KSa | 1 | |
| | type II aromatic | tetracenomycin KSa | 1 | |
| | type II aromatic | unclassified KSa | 1 | |

## References


(1) Ziemert, N.; Podell, S.; Penn, K.; Badger, J. H.; Allen, E.; Jensen, P. R. The Natural Product Domain Seeker NaPDoS: A Phylogeny Based Bioinformatic Tool to Classify Secondary Metabolite Gene Diversity. *PLoS One* **2012**, *7* (3), e34064. https://doi.org/10.1371/journal.pone.0034064.

(2) Blin, K.; Shaw, S.; Kloosterman, A. M.; Charlop-Powers, Z.; van Wezel, G. P.; Medema, M. H.; Weber, T. AntiSMASH 6.0: Improving Cluster Detection and Comparison Capabilities. *Nucleic Acids Res.* **2021**, 1–7. https://doi.org/10.1093/nar/gkab335.

(3) Helfrich, E. J. N.; Ueoka, R.; Dolev, A.; Rust, M.; Meoded, R. A.; Califano, G.; Costa, R.; Gugger, M.; Steinbeck, C.; Moreno, P.; Piel, J. Automated Structure Prediction of Trans-Acyltransferase Polyketide Synthase Products. *Nat Chem Biol* **2019**, *15* (August). https://doi.org/10.1038/s41589-019-0313-7.

(4) Bachmann, B. O.; Ravel, J. *Chapter 8 Methods for In Silico Prediction of Microbial Polyketide and Nonribosomal Peptide Biosynthetic Pathways from DNA Sequence Data*, 1st ed.; Elsevier Inc., **2009**; Vol. 458. https://doi.org/10.1016/S0076-6879(09)04808-3.

(5) Kautsar, S. A.; Blin, K.; Shaw, S.; Navarro-Muñoz, J. C.; Terlouw, B. R.; van der Hooft, J. J. J.; van Santen, J. A.; Tracanna, V.; Suarez Duran, H. G.; Pascal Andreu, V.; Selem-Mojica, N.; Alanjary, M.; Robinson, S. L.; Lund, G.; Epstein, S. C.; Sisto, A. C.; Charkoudian, L. K.; Collemare, J.; Linington, R. G.; Weber, T.; Medema, M. H. MIBiG 2.0: A Repository for Biosynthetic Gene Clusters of Known Function. *Nucleic Acids Res.* **2020**, *48* (D1), D454–D458. https://doi.org/10.1093/nar/gkz882.

(6) Lu, S.; Wang, J.; Chitsaz, F.; Derbyshire, M. K.; Geer, R. C.; Gonzales, N. R.; Gwadz, M.; Hurwitz, D. I.; Marchler, G. H.; Song, J. S.; Thanki, N.; Yamashita, R. A.; Yang, M.; Zhang, D.; Zheng, C.; Lanczycki, C. J.; Marchler-Bauer, A. CDD/SPARCLE: The Conserved Domain Database in 2020. *Nucleic Acids Res.* **2020**, *48* (D1), D265–D268. https://doi.org/10.1093/nar/gkz991.

(7) Marchler-Bauer, A.; Anderson, J. B.; Derbyshire, M. K.; DeWeese-Scott, C.; Gonzales, N. R.; Gwadz, M.; Hao, L.; He, S.; Hurwitz, D. I.; Jackson, J. D.; Ke, Z.; Krylov, D.; Lanczycki, C. J.; Liebert, C. A.; Liu, C.; Lu, F.; Lu, S.; Marchler, G. H.; Mullokandov, M.; Song, J. S.; Thanki, N.; Yamashita, R. A.; Yin, J. J.; Zhang, D.; Bryant, S. H. CDD: A Conserved Domain Database for Interactive Domain Family Analysis. *Nucleic Acids Res.* **2007**, *35* (D1), 237–240. https://doi.org/10.1093/nar/gkl951.

(8) Letunic, I.; Bork, P. Interactive Tree Of Life (ITOL) v4: Recent Updates and New Developments. *Nucleic Acids Res.* **2019**, *47* (W1), W256–W259. https://doi.org/10.1093/nar/gkz239.

(9) Edgar, R. C. MUSCLE: Multiple Sequence Alignment with High Accuracy and High Throughput. *Nucleic Acids Res.* **2004**, *32* (5), 1792–1797. https://doi.org/10.1093/nar/gkh340.

(10) Price, M. N.; Dehal, P. S.; Arkin, A. P. FastTree 2 - Approximately Maximum-Likelihood Trees for Large Alignments. *PLoS One* **2010**, *5* (3). https://doi.org/10.1371/journal.pone.0009490.

(11) Miller, M. A.; Pfeiffer, W.; Schwartz, T. Creating the CIPRES Science Gateway for Inference of Large Phylogenetic Trees. In *2010 Gateway Computing Environments Workshop, GCE 2010*; New Orleans, LA, **2010**; pp 1–8. https://doi.org/10.1109/GCE.2010.5676129.

(12) Jiang, C.; Kim, S. Y.; Suh, D. Y. Divergent Evolution of the Thiolase Superfamily and Chalcone Synthase Family. *Mol. Phylogenet. Evol.* **2008**, *49* (3), 691–701. https://doi.org/10.1016/j.ympev.2008.09.002.

(13) Darriba, D.; Taboada, G. L.; Doallo, R.; Posada, D. ProtTest 3: Fast Selection of Best-Fit Models of Protein Evolution. *Bioinformatics* **2011**, *27* (8), 1164–1165. https://doi.org/10.1007/978-3-642-21878-1_22.

(14) Stamatakis, A. RAxML Version 8: A Tool for Phylogenetic Analysis and Post-Analysis of Large Phylogenies. *Bioinformatics* **2014**, *30* (9), 1312–1313. https://doi.org/10.1093/bioinformatics/btu033.

(15) Elfeki, M.; Alanjary, M.; Green, S. J.; Ziemert, N.; Murphy, B. T. Assessing the Efficiency of Cultivation Techniques to Recover Natural Product Biosynthetic Gene Populations from Sediment. *ACS Chem. Biol.* **2018**. https://doi.org/10.1021/acschembio.8b00254.

(16) Gumerov, V. M.; Zhulin, I. B. TREND: A Platform for Exploring Protein Function in Prokaryotes Based on Phylogenetic, Domain Architecture and Gene Neighborhood Analyses. *Nucleic Acids Res.* **2020**, *48* (1), W72–W76. https://doi.org/10.1093/NAR/GKAA243.

(17) Millán-Aguiñaga, N.; Chavarria, K. L.; Ugalde, J. A.; Letzel, A.-C.; Rouse, G. W.; Jensen, P. R. Phylogenomic Insight into *Salinispora* (Bacteria, Actinobacteria) Species Designations. *Sci. Rep.* **2017**, *7*, 3564. https://doi.org/10.1038/s41598-17-02845-3.

(18) Wawrik, B.; Kerkhof, L.; Zylstra, G. J.; Kukor, J. J. Identification of Unique Type II Polyketide Synthase Genes in Soil. *Appl. Environ. Microbiol.* **2005**, *71* (5), 2232–2238. https://doi.org/10.1128/AEM.71.5.2232-2238.2005.

(19) Cai, H.; Li, Q.; Fang, X.; Li, J.; Curtis, N. E.; Altenburger, A.; Shibata, T.; Feng, M.; Maeda, T.; Schwartz, J. A.; Shigenobu, S.; Lundholm, N.; Nishiyama, T.; Yang, H.; Hasebe, M.; Li, S.; Pierce, S. K.; Wang, J. Data Descriptor: A Draft Genome Assembly of the Solar-Powered Sea Slug Elysia Chlorotica. *Sci. Data* **2019**, *6*, 1–13. https://doi.org/10.1038/sdata.2019.22.

(20) Torres, J. P.; Lin, Z.; Winter, J. M.; Krug, P. J.; Schmidt, E. W. Animal Biosynthesis of Complex Polyketides in a Photosynthetic Partnership. *Nat. Commun.* **2020**, *11* (1), 1–12. https://doi.org/10.1038/s41467-020-16376-5.

(21) Schorn, M. A.; Verhoeven, S.; Ridder, L.; Huber, F.; Acharya, D. D.; Aksenov, A. A.; Aleti, G.; Moghaddam, J. A.; Aron, A. T.; Aziz, S.; Bauermeister, A.; Bauman, K. D.; Baunach, M.; Beemelmanns, C.; Beman, J. M.; Berlanga-Clavero, M. V.;

Blacutt, A. A.; Bode, H. B.; Boullie, A.; Brejnrod, A.; Bugni, T. S.; Calteau, A.; Cao, L.; Carrión, V. J.; Castelo-Branco, R.; Chanana, S.; Chase, A. B.; Chevrette, M. G.; Costa-Lotufo, L. V.; Crawford, J. M.; Currie, C. R.; Cuypers, B.; Dang, T.; de Rond, T.; Demko, A. M.; Dittmann, E.; Du, C.; Drozd, C.; Dujardin, J. C.; Dutton, R. J.; Edlund, A.; Fewer, D. P.; Garg, N.; Gauglitz, J. M.; Gentry, E. C.; Gerwick, L.; Glukhov, E.; Gross, H.; Gugger, M.; Guillén Matus, D. G.; Helfrich, E. J. N.; Hempel, B. F.; Hur, J. S.; Iorio, M.; Jensen, P. R.; Kang, K. Bin; Kaysser, L.; Kelleher, N. L.; Kim, C. S.; Kim, K. H.; Koester, I.; König, G. M.; Leao, T.; Lee, S. R.; Lee, Y. Y.; Li, X.; Little, J. C.; Maloney, K. N.; Männle, D.; Martin H, C.; McAvoy, A. C.; Metcalf, W. W.; Mohimani, H.; Molina-Santiago, C.; Moore, B. S.; Mullowney, M. W.; Muskat, M.; Nothias, L. F.; O'Neill, E. C.; Parkinson, E. I.; Petras, D.; Piel, J.; Pierce, E. C.; Pires, K.; Reher, R.; Romero, D.; Roper, M. C.; Rust, M.; Saad, H.; Saenz, C.; Sanchez, L. M.; Sørensen, S. J.; Sosio, M.; Süssmuth, R. D.; Sweeney, D.; Tahlan, K.; Thomson, R. J.; Tobias, N. J.; Trindade-Silva, A. E.; van Wezel, G. P.; Wang, M.; Weldon, K. C.; Zhang, F.; Ziemert, N.; Duncan, K. R.; Crüsemann, M.; Rogers, S.; Dorrestein, P. C.; Medema, M. H.; van der Hooft, J. J. J. A Community Resource for Paired Genomic and Metabolomic Data Mining. *Nature Chemical Biology*. **2021**, pp 363–368. https://doi.org/10.1038/s41589-020-00724-z.

(22) Owen, J. G.; Reddy, B. V. B.; Ternei, M. A.; Charlop-Powers, Z.; Calle, P. Y.; Kim, J. H.; Brady, S. F. Mapping Gene Clusters within Arrayed Metagenomic Libraries to Expand the Structural Diversity of Biomedically Relevant Natural Products. *Proc. Natl. Acad. Sci. U. S. A.* **2013**, *110* (29), 11797–11802. https://doi.org/10.1073/pnas.1222159110.

(23) Borsetto, C.; Amos, G. C. A.; Nunes da Rocha, U.; Mitchell, A. L.; Finn, R. D.; Laidi, R. F.; Vallin, C.; Pearce, D. A.; Newsham, K. K.; Wellington, E. M. H. Microbial Community Drivers of PK / NRP Gene Diversity in Selected Global Soils. *Microbiome* **2019**, 1–11.
